# How error correction affects polymerase chain reaction deduplication: A survey based on unique molecular identifier datasets of short reads

**DOI:** 10.1002/qub2.99

**Published:** 2025-03-23

**Authors:** Pengyao Ping, Tian Lan, Shuquan Su, Wei Liu, Jinyan Li

**Affiliations:** ^1^ School of Computer Science Faculty of Engineering and Information Technology University of Technology Sydney Ultimo New South Wales Australia; ^2^ Data Science Institute University of Technology Sydney Ultimo New South Wales Australia; ^3^ School of Computer Science and Control Engineering Shenzhen Institute of Advanced Technology Chinese Academy of Sciences Shenzhen China

**Keywords:** error correction, next generation sequencing (NGS), PCR‐deduplication, polymerase chain reaction (PCR) duplicates, unique molecular identifier (UMI)

## Abstract

Next‐generation sequencing data are widely utilised for various downstream applications in bioinformatics and numerous techniques have been developed for PCR‐deduplication and error‐correction to eliminate bias and errors introduced during the sequencing. This study first‐time provides a joint overview of recent advances in PCR‐deduplication and error‐correction on short reads. In particular, we utilise UMI‐based PCR‐deduplication strategies and sequencing data to assess the performance of the solely‐computational PCR‐deduplication approaches and investigate how error correction affects the performance of PCR‐deduplication. Our survey and comparative analysis reveal that the deduplicated reads generated by the solely‐computational PCR‐deduplication and error‐correction methods exhibit substantial differences and divergence from the sets of reads obtained by the UMI‐based deduplication methods. The existing solely‐computational PCR‐deduplication and error‐correction tools can eliminate some errors but still leave hundreds of thousands of erroneous reads uncorrected. All the error‐correction approaches raise thousands or more new sequences after correction which do not have any benefit to the PCR‐deduplication process. Based on our findings, we discuss future research directions and make suggestions for improving existing computational approaches to enhance the quality of short‐read sequencing data.

## 1 INTRODUCTION

Next‐generation sequencing (NGS) has revolutionized genomics research, enabling extensive acquisition of genome‐wide data with unprecedented speed, precision and cost‐effectiveness and making significant progress in the field of DNA sequencing (DNA‐Seq) [1] and RNA sequencing (RNA‐seq) [2]. NGS data play a vital role in various downstream analyses, including estimation of microbial diversity [3], variant calling [4], immune cell responses [5, 6], RNA quantification [7], cancer mutation detection [8], de novo genome assembly [9], de novo transcriptome assembly [10] and nonclinical genotoxicity and carcinogenicity testing [11], detecting allele‐specific expression [12], isomiR identification [13] and genome base editing [14]. One of the key steps in NGS is the polymerase chain reaction (PCR) process which is purposely used in the library construction and cluster amplification to increase the number of DNA/RNA molecule fragments. This amplified library or flowcell containing multiple copies of each original molecule fragment is helpful for a reliable sequencing of these molecule fragments into digital reads. Such identical reads from the replicates of one molecule fragment are referred to as the PCR duplicates of the read of the original molecule fragment [15, 16].

However, PCR has biases or preferences in the amplification of certain types of molecule fragments, meaning that the duplicate number of a molecule fragment can be quite different from others with a varying amplification rate. Sometimes, it can generate hybrid molecules. Errors occurring in the early cycles of PCR are often inherited in subsequent cycles, resulting in a complex error distribution [17]. These uneven amplification rates and intricate error patterns complicate many downstream NGS applications such as estimation of microbial diversity and composition [3], variant calling [4], immune cell heterogeneity understanding [5, 6], RNA quantification [7] and cancer mutation detection [8]. To this end, numerous biological and computational methods for PCR‐deduplication have been proposed over the past years. PCR‐deduplication identifies and removes duplicated reads in a fasta/fastq file, leaving only unique reads as true biological sequences for downstream analysis. The deduplication process involves sequence comparisons of expensive complexity to find exact and unprecise duplicates for retaining only the unique copies of the reads [18, 19, 20, 21].

In addition to these PCR biases and errors, NGS also makes errors during other steps such as at the sample handling step or the base calling step (due to fluorophore crosstalk) [22, 23]. The sequencing errors stemming from these sources add more confusion to the accuracy of downstream data analysis, especially in de novo genome assembly [9], de novo transcriptome assembly [10] and nonclinical genotoxicity and carcinogenicity testing [11]. Therefore, it is crucial to overcome all of these PCR and other steps’ sequencing biases and errors whenever NGS data are involved to ensure the reliability and accuracy of downstream analyses [5, 24].

Error‐correction NGS technology is defined as the method that identifies erroneous bases no matter caused by PCR or by fluorophore crosstalk as many as possible in a fasta/fastq dataset and then turns these erroneous bases into their correct formats.

**FIGURE 1:**
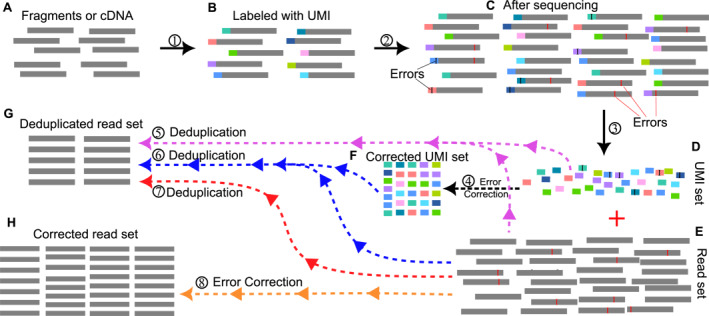
Schematic overview of molecular tracking, PCR‐deduplication and error‐correction processes. (A–C) Illustrate the utilization of unique molecular identifiers (UMIs) for precise molecule tracking, accounting for errors introduced during sequencing, where errors may manifest in both UMI sequences and read sequences. (D–G) Describe the PCR‐deduplication process: ⑤ signifies the removal of duplicated reads using UMI tags, while ④ ‐F‐ ⑥ depicts the elimination of duplicates with corrected UMI tags. ⑦ represents duplicate read removal using only computational methods. (E− ⑧ ‐H) elucidate the computational error‐correction process aimed at eliminating all the errors introduced during sequencing. It is important to note that the deduplicated read sets vary depending on the PCR‐deduplication process applied, and excessive correction by error‐correction methods may unintentionally remove genuine original reads.

PCR‐deduplication makes the size of the original sequencing data (fasta or fastq files) much smaller as it aims to maintain only the unique genuine biological reads, while error‐correction never changes the number of reads in the original sequencing data—it just identifies erroneous reads in the dataset and makes corrections on them. Although PCR‐deduplication and error‐correction methods employ different strategies to achieve different goals, they have a common technical component to eliminate sequencing uncertainty such as biases and errors. Recent PCR‐deduplication algorithms [25, 26] also consider fluorophore crosstalk errors during the deduplication process. Some deduplicated results for example, by Calib [27] and DAUMI [26] can be restored to their original data using the original sequencing IDs stored in the Calib description section or using the read abundance levels stored in the DAUMI description section of the deduplicated read set files. Merging tasks of PCR‐deduplication and error‐correction is relatively straightforward. The more significant challenge is distinguishing whether the sequencing errors originate from PCR amplification or sequencing instruments. Figure 1 is a schematic diagram depicting an overview of molecular tracking, PCR‐deduplication and error‐correction processes. It is important to recognise that some PCR‐deduplication tools incorporate error correction processes. Therefore, in this study, the term “error correction” encompasses the correction procedures employed in both deduplication and error correction methods. Meanwhile, “error‐correction” specifically denotes methods solely focused on error‐correction techniques for sequencing data, which identify erroneous reads in the dataset and make corrections to them.

To our knowledge, there is no joint investigation of PCR‐deduplication and error‐correction, nor research work assessing the impact of error correction on PCR‐deduplication so far. Instead, most of the methods are only for read duplicate removal or separately for error correction assessment on simulated sequencing datasets. In fact, evaluating the performance of these algorithms is challenging due to the absence of ground truth in the sequencing fragments. Utilising unique molecular identifiers (UMIs) provides one of the most effective strategies for recording and tracking sequencing errors during the PCR sequencing process [5, 24]. In particular, error‐correction methods have been extensively evaluated in the literature using constructed UMI‐based ground truth datasets [28].

This study briefly reviews current methodologies for PCR‐deduplication and error‐correction and performs a comparative analysis from novel perspectives using UMI short‐read sequencing datasets. An ideal algorithm for alleviating uncertainties from the PCR and fluorophore crosstalk biases and errors should achieve two key objectives: (1) identifying and preserving the authentic biological sequences and (2) maintaining precise read abundance levels without introducing new errors or sequences; therefore, this study

- compares the differences of deduplicated sets of reads generated by different UMI‐based PCR‐deduplication methods;
- utilises UMI‐based PCR‐deduplication methods to assess the performance of solely‐computational PCR‐deduplication tools;
- examines the percentage of corrected reads and the number of wrongly introduced new sequences after the error‐correction process;
- investigates the impact of error correction procedures of PCR‐deduplication and error‐correction methods on deduplication for short reads.

## 2 PCR‐DEDUPLICATION FOR SHORT READS: A BRIEF REVIEW

Existing PCR‐deduplication methods can be categorised into two categories. The main idea of the first category is to utilise biochemical techniques such as UMIs to accurately count and track precise and imprecise molecule fragments. While the second category takes only computational steps for the PCR‐deduplication. Both the UMI and the solely‐computational methods can be further classified according to whether they use reference genome sequences. These methods are summarised in Table 1 with more details.

**TABLE 1:** Summary of the PCR‐deduplication approaches with and without use of Unique Molecular Identifiers (UMIs) data.

UMI‐based	Methods	Alignment free	UMI errors correction	Read correction/mismatches allowed	Library layout	Year	Times cited^a^	Reference
Yes	Je	No	No	No	SE/PE	2016	112	[30]
	UMI‐Reducer	No	No	No	SE/PE	2017	/	[31]
	UMI‐tools	No	Yes	Yes	SE/PE	2017	1126	[25]
	AmpUMI	Yes	Yes	Yes	SE/PE	2018	28	[36]
	zUMIs	No	Yes	No	SE/PE	2018	212	[33]
	Umitools	No	Yes	No	PE	2018	110	[37]
	Calib	Yes	Yes	Yes	PE	2019	17	[27]
	UMICollapse	No	Yes	Yes	SE/PE	2019	9	[34]
	UMIc	Yes	Yes	Yes	SE/PE	2021	10	[35]
	DAUMI	Yes	Yes	Yes	SE/PE	2023	4	[26]
Yes/No	Gencore	No	No	Yes	PE	2019	42	[32]
	markdup(SAMtools)	No	No	No	SE/PE	2009	14853	[38]
No	CD‐HIT‐DUP	Yes	NA	Yes	SE/PE	2010	2048	[45]
	SEED	Yes	NA	Yes	SE/PE	2011	55	[48]
	FastUniq	Yes	NA	No	PE	2012	246	[73]
	Fulcrum	Yes	NA	Yes	SE/PE	2012	29	[19]
	Rainbow	Yes	NA	Yes	SE/PE	2012	100	[46]
	MarkDuplicates(Picard)	No	NA	No	No	2014	/	[29]
	pRESTO	Yes	NA	Yes	SE/PE	2014	323	[47]
	ParDRe	Yes	NA	Yes	SE/PE	2016	28	[43]
	MarDRe	Yes	NA	Yes	SE/PE	2017	11	[44]
	PCRduplicates	No	NA	No	SE/PE	2017	23	[40]
	NGSReadsTreatment	Yes	NA	No	SE/PE	2019	6	[41]
	Nubeam‐dudup	Yes	NA	No	SE/PE	2019	6	[42]
	BioSeqZip	Yes	NA	No	SE/PE	2020	5	[72]
	AmpliCI	Yes	NA	Yes for PE	SE/PE	2021	11	[75]
	Minirmd	Yes	NA	Yes	SE/PE	2020	13	[18]
	Fastp	Yes	No	Yes for PE	SE/PE	2021	11625	[71]

^a^ The citation count for each method refers to its total times cited across all databases in the Web of Science as of 15 September 2024. The symbol “/” indicates the corresponding method was not formally published and/or indexed by the Web of Science. Citation counts reflect the overall usage of the tools across various applications, not limited to PCR deduplication alone. A lower citation frequency does not imply that the method lacks utility or effectiveness.

### 2.1 PCR‐deduplication methods working on UMI datasets

During the library preparation step in NGS, UMIs are added into the library to attach to a specific location for every DNA fragment before PCR amplification. In ideal cases, each unique molecular fragment corresponds to a UMI. Therefore, unprecise duplicates of a molecular fragment after PCR amplification can be recognised through their UMI sequences when there exist no PCR errors in the UMI sequences.

Alignment‐based PCR‐deduplication methods distinguish duplicated reads by grouping the reads using mapping positions and identical UMIs under the assumption that there exist no PCR errors in the UMI sequences. The typical process of these strategies clusters the reads with mapping positions first and then regroups reads in the same cluster based on UMIs. For instance, Girardot et al. developed a suite of tools named Je—an extension of the popular PCR‐deduplication tool Picard’s MarkDuplicates [29] by supporting more accurate duplicate removal. Je identifies duplicates based on mapping positions to the reference and keeps the reads with distinct UMIs for downstream processing [30]. Similarly, UMI‐Reducer identifies and collapses PCR duplicates using identical UMI sequences and the mapping location of the read. It also treats these reads that have the same mapping positions but different UMIs as unique reads [31]. Gencore groups the mapped sequences and generates consensus sequences by merging these reads within each cluster. This process can clear PCR biases and errors during the generation of consensus reads. When working with sequencing data that include UMI tags, Gencore could cluster reads based on the UMIs for the identification of reads originating from the same fragments [32].

Unlike those tools which did not consider the tiny amount of UMI errors made by the PCR amplification for PCR‐deduplication, Smith et al. reported that mistaken bases in UMIs are common after PCR [25] and then they developed a network‐based method, UMI‐tools, to correct UMI errors before PCR‐deduplication. In the correction process, UMI‐tools constructs a UMI network where two UMIs are linked if they have a one‐base difference; then, the method uses three techniques to determine the count of distinct molecules at a particular genomic position. The main goal of these techniques is to simplify the network by identifying a representative UMI that represents the entire network rather than identifying the exact sequence of the original UMIs [25]. Additionally, zUMIs, a method designed to process multiplexed RNA‐seq data that contain known and random BCs and UMIs, employs the strategy proposed in UMI‐tools to handle UMI errors when necessary. The pipeline removes reads with low‐quality BCs and UMIs and then it maps the remaining reads to a reference genome [33].

Not relying on a reference genome, alignment‐free methods do not map reads to a reference during PCR‐deduplication. This approach avoids issues such as clustering reads that have multiple mapping locations and it still works when no references are available for mapping. Alignment‐free methods typically retain the reads with the highest UMI counts and quality scores but sometimes they vary these parameters [34, 35]. For example, Calib conducts duplicate removal by constructing a graph based on UMI and sequence similarity through hashing and MinHashing techniques. In the constructed graph, each connected subgraph is identified as a cluster. One limitation is that Calib can only handle clustering paired‐end reads with UMIs produced from Illumina platforms [27]. UMIc rectifies base errors in UMI sequences by leveraging base frequency and quality information. The correction process is initially implemented for reads sharing identical UMI tags. Subsequently, UMIc merges UMIs based on both UMI‐to‐UMI distances and sequence‐to‐sequence distances. Finally, it addresses errors in sequences belonging to the same group of merged UMIs [35]. AmpUMI performs sequencing error correction during the PCR‐deduplication process. In essence, this method involves clustering sequences based on UMIs and retaining the most frequently occurring sequences as biological reads. The remaining sequences are then discarded as they are considered to be attributable to sequencing errors [36].

Moreover, Fu et al. introduced experimental protocols and computing approaches by integrating UMIs into standard RNA‐seq procedures to determine PCR copies in RNA‐seq and small‐RNA sequencing data. Their study demonstrated a superior accuracy of duplicate removal when employing UMI sequence data for comparison with those without UMIs. Notably, without the use of UMIs, numerous biologically meaningful reads are mistakenly flagged as duplicates during the removal process [37]. Recently, a probabilistic algorithm, DAUMI [26], was designed for amplicon sequencing to identify actual biological sequences and estimate the read abundance by eliminating PCR and fluorophore crosstalk biases and errors in the UMIs and reads. One advantage of DAUMI is that it can deal with UMI collisions, even on highly similar sequences.

### 2.2 PCR‐deduplication approaches without use of UMI data

Without using UMI tags, most of the solely‐computational PCR‐deduplication methods aim to reduce time complexity and computational memory usage for removing precise duplicate or unprecise, nearly‐duplicate reads. Their key step is to develop parallel computing strategies and advanced data structures for enhancing PCR‐deduplication efficiency.

These computational methods, including markdup in SAMtools [38, 39], MarkDuplicates in picard [29], PCRduplicates [40] and Gencore [32], also depend on the alignment data of the reads to reference genomes helping the removal of the duplicate reads. These methods have experienced a loss of accuracy when reads have multiple mapping locations and are rendered ineffective in scenarios where no references are available for mapping.

Alignment‐free methods employ advanced data structures to recognise duplicated reads by comparing the differences in a time‐ and memory‐efficient manner. For instance, NGSReadsTreatment [41] was designed to remove redundancies from the raw reads with the Cuckoo Filter, an enhancement of the Bloom Filter, using cuckoo hashing to prevent collisions. During the redundancy removal, NGSReadsTreatment keeps a read if its fingerprint is not found in the cuckoo hash table; otherwise, discard. Nubeam‐dudup [42] employs unique numbers to represent reads via matrix product calculation by representing nucleotides as matrices and then using the unique numbers instead of reads themselves for pairwise comparison to identify duplicated reads. These methods only keep existing identical reads without considering PCR bias and error elimination for PCR‐deduplication. In addition, most existing methods split the whole data set into chunks and perform pairwise comparisons in each cluster to reduce the time complexity of the pairwise comparison of all the reads. For instance, Fulcrum [19], ParDRe [43] and MarDRe [44] have employed a prefix‐clustering technique, while still exploiting a greedy incremental clustering algorithm. Minirmd [18] clusters reads using minimiser for multiple rounds to remove duplicates and near‐duplicates.

Moreover, some tools allow mismatches and perform read correction in the awareness of PCR bias and error removal. For instance, CD‐HIT‐DUP [45], Rainbow [46], pRESTO [47], ParDRe [43], MarDRe [44] and Minirmd [18] allow a user‐defined number of mismatches while SEED [48] and Fulcrum [19] allow up to three mismatches during removing duplicated reads. Eliminating PCR biases and errors while identifying duplicated reads is more complex than the pure PCR‐deduplication. These approaches with tolerance to small amounts of mismatches can correct some PCR errors and remove near‐duplicate reads. However, these methods may only discover some expected base errors since they conduct an approximate pairwise comparison across the whole reads.

## 3 ERROR‐CORRECTION METHODS: A BRIEF REVIEW

Error‐correction algorithms use various statistical/mathematical ideas to detect and correct errors to improve the quality of sequencing data. To our knowledge, existing error‐correction methods are all computational approaches without using UMI information. These error‐correction methods in Table 2 can be classified into *k*‐mer‐based, multiple sequence alignment (MSA) based algorithms and de Bruijn graph (DBG) based methods. In addition to these general‐purpose methods, several other error‐correction methods were designed for specific sequencing tasks, which we classified as scenario‐based error‐correction.

**TABLE 2:** Summary of the computational error‐correction methods.

Methods type	Methods	Year	Times cited^a^	Reference
*K*‐mer‐based	SGA‐EC	2012	619	[55]
	Musket	2012	459	[53]
	RACER	2013	71	[54]
	Lighter	2014	172	[49]
	Blue	2014	60	[50]
	BFC	2015	139	[56]
	Pollux	2015	38	[57]
	BLESS	2014	101	[51]
	BLESS 2	2016	27	[52]
	RECKONER	2017	24	[58]
	Athena	2019	8	[59]
	Lerna	2022	5	[60]
MLA‐based	Coral	2011	140	[61]
	ECHO	2011	89	[62]
	Fiona	2014	51	[63]
	Karect	2015	76	[64]
	CARE	2021	10	[65]
	CARE 2.0	2022	7	[66]
DBG‐based	BrownieCorrector	2019	23	[67]
	Bcool	2020	20	[68]
Scenario‐based	InsEC	2021	2	[69]
	miREC	2021	4	[70]

^a^ The citation count for each method refers to its total times cited across all databases in the Web of Science as of 15 September 2024. The symbol “/” indicates the corresponding method was not formally published and/or indexed by the Web of Science. Citation counts reflect the overall usage of the tools across various applications, not limited to PCR deduplication alone. A lower citation frequency does not imply that the method lacks utility or effectiveness.

### 3.1 *K*‐mer‐based methods

*K*‐mer is a sub‐string of a sequence; given a frequency threshold, *k*‐mer‐based algorithms aim to determinate high‐frequency *k*‐mers, namely solid *k*‐mers, otherwise marked as weak *k*‐mers. Then, these algorithms replace weak *k*‐mers with matching solid *k*‐mers. Some of them were specifically designed to correct substitution errors. For instance, Lighter [49] and Blue [50] employ a *k*‐mer‐based strategy and quality scores to target substitution error correction. BLESS [51] and its successor, BLESS 2 [52], concentrate on rectifying substitution errors in sequencing reads through *k*‐mer analysis‐based Bloom filters. BLESS 2, in particular, demonstrates enhanced performance in both speed and accuracy, especially when handling larger datasets, compared to the original BLESS. Musket [53] and RACER [54] primarily address substitution errors. In contrast, SGA‐EC [55], BFC [56], Pollux [57] and RECKONER [58] were designed to tackle various types of errors, including substitutions, insertions and deletions.

In addition, Athena [59] and Lerna [60] were proposed using deep learning frameworks such as recurrent neural network and attention‐based transformers to automatically find the optimal *k*‐mer size to improve the performance of the *k*‐mer based methods. However, these two tools’ source codes or software are not publicly available.

### 3.2 MSA‐based methods

MSA‐based methods utilise the MSA information, including base coverage and erroneous positions, to group the reads with shared knowledge and generate a contig as an error‐free reference to correct false bases. Earlier methods were represented by Coral [61], ECHO [62], Fiona [63] and Karect [64]. MSA‐based approaches also incorporate other strategies rather than not solely utilising the MLA technique. For instance, Karect treats each read as a reference to construct an initial partial‐order graph and then conducts multiple alignments for an optimized group of sequences similar to the reference read to update the partial‐order graph. Next, it utilises the accumulated partial alignment results to rectify the reference read.

In addition, CARE [65] implemented in 2021 aims to produce fewer false‐positive corrections while achieving more true positives by searching similarity within extensive read collections based on mini hashing. Then, CARE 2.0 [66], an extension of CARE, was developed using the machine learning method of random forests to increase the correction precision and sensitivity further. One common drawback of these MSA‐based algorithms is that constructing optimal MSAs is of high computational complexity and time‐consumption.

Moreover, the performance of some methods, such as those by Fiona [63] and RACER [54], may be influenced by the preset parameters (e.g. the estimated genome size) because it is tricky for users or it is not suitable to estimate the genome size used for non‐whole‐genome sequencing data (e.g. synthetic sequencing).

### 3.3 DBG‐based methods

BrownieCorrector [67] and Bcool [68] rely on constructing DBG to detect and rectify errors in reads. Both of them utilised *k*‐mer‐based techniques for constructing reference DBG. Then erroneous nodes are detected and cleaned from the reference DBG based on coverage and graph topology. Finally, the error reads are corrected to their normal state by aligning them to the corrected DBG. These two methods adopt the same data structures but differ in implementation details and BrownieCorrector is applicable to only paired‐end sequencing datasets.

### 3.4 Scenario‐based error‐correction

The non‐uniform sequencing depths restrict the above methods’ effectiveness for error removal in specific sequencing scenarios. For example, when analysing single nucleotide polymorphism, particular genes or pathways involved in the disease mechanism or one specific genome segment would be more important than the other loci in the genome.

General‐purpose correction methods may under‐correct or over‐correct the sequencing errors in short reads of disease‐associated genes when targeted gene sequencing is applied. An instance‐based algorithm, InsEC [69], was proposed to rectify the errors in the reads of a disease‐related gene using local sequence characteristics and statistics directly correlated to these genes. Focusing on correcting miRNA sequencing errors, an error rectification method, miREC [70], was proposed using a lattice structure combining *k*‐mers, (*k*‐1)‐mers and (*k*+1)‐mers to maintain the frequency differences of the *k*‐mers. Notably, InsEC and miREC have the same advantage of not introducing new errors when performing error corrections. In addition, there are other scenarios for correcting errors in short reads. For example, to eliminate the UMI errors introduced during PCR and nucleotide miscalling and indels during sequencing, the process of eliminating UMI errors was introduced by UMI‐tools [25] and DAUMI [26], respectively, during PCR‐deduplication.

## 4 HOW ERROR CORRECTION AFFECTS PCR DEDUPLICATION: PERFORMANCE COMPARISON ON UMI DATASETS

We took 11 UMI‐based single‐end sequencing datasets of the accession IDs SRR1543964‐SRR1543971, SRR28313972, SRR28313990 and SRR28314008 together with a pair‐end sequencing dataset of the accession ID SRR11207257 for the comparative analysis. These datasets SRR1543964‐SRR1543971 and SRR11207257 were previously employed in the benchmark study conducted by Mitchell et al. [28]. For those datasets, the first twelve bases of each sequence represent the sequence of the UMI, while the subsequent twelve bases serve as an index for multiplexing. We extracted the sequences of these UMIs and stored them in the description section of each record of the sequencing data. The multiplexing tags were removed using the Fastp tool [71]. The group of datasets SRR28313972, SRR28313990 and SRR28314008 were recently sequenced datasets that were published in 2024 and the UMI sequence with length of 11 is stored in the description section.

### 4.1 Performance by PCR‐deduplication methods and their comparison

**FIGURE 2:**
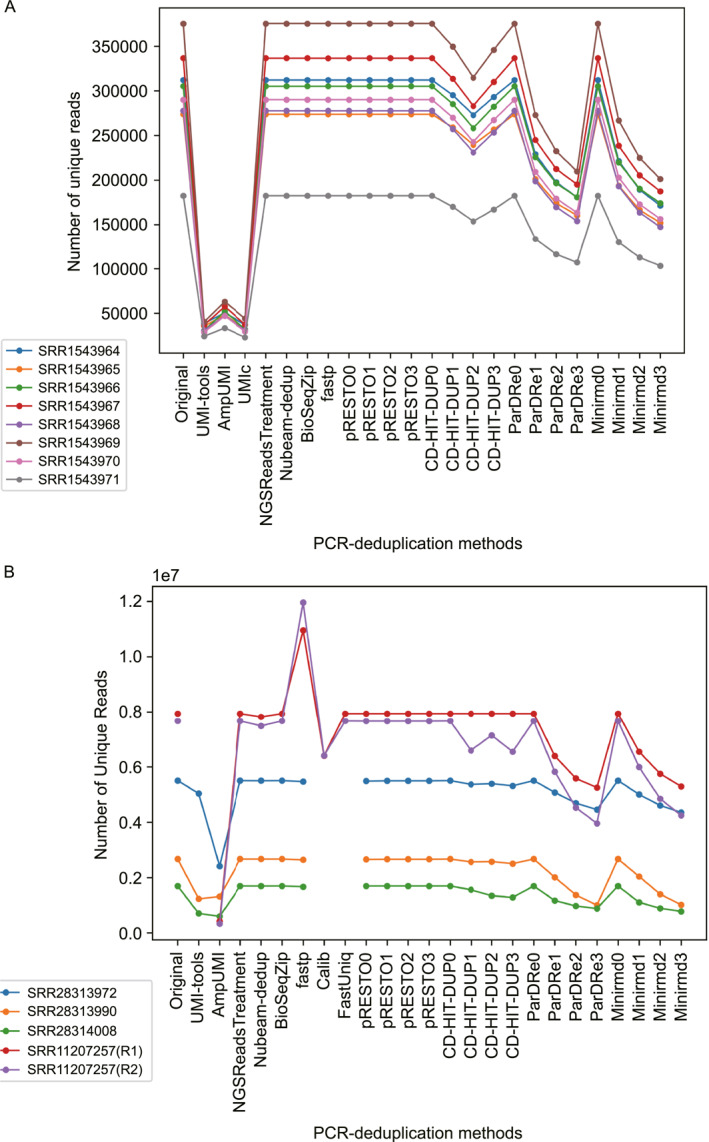
Line charts compare the number of unique reads after PCR‐deduplication using the methods UMI‐tools [25], AmpUMI [36], Calib [27], UMIc [35], NGSReadsTreatment [41], Nubeam‐dedup [42], BioSeqZip [72], Fastp [71], FastUniq [73], pRESTO [47], CD‐HIT‐DUP [45], ParDRe [43] and Minirmd [18]. The break in the line chart indicates that the method does not support single or paired‐end datasets for PCR‐deduplication or cannot produce valid results on the corresponding datasets.

We focused on four UMI‐based PCR‐deduplication methods, UMI‐tools [25], AmpUMI [36], Calib [27] and UMIc [35] and 9 solely‐computational methods NGSReadsTreatment [41], Nubeam‐dedup [42], BioSeqZip [72], Fastp [71], FastUniq [73], pRESTO [47], CD‐HIT‐DUP [45], ParDRe [43] and Minirmd [18] for our comparative analysis. The number of unique reads remaining after PCR‐deduplication by the different algorithms is shown in Figure 2, with the exact read counts provided in Table S1. Table 3 lists the number of new sequences introduced by each algorithm after PCR‐deduplication.

**TABLE 3:** The number of introduced new sequences after PCR‐deduplication by the methods of UMI‐tools [25], AmpUMI [36], Calib [27], UMIc [35], NGSReadsTreatment [41], Nubeam‐dedup [42], BioSeqZip [72], Fastp [71], FastUniq [73], pRESTO [47], CD‐HIT‐DUP [45], ParDRe [43] and Minirmd [18] on the data sets SRR1543964‐SRR1543971, SRR28313972, SRR28313990, SRR28314008 and SRR11207257, respectively.

Dataset	UMI‐tools	AmpUMI	Calib	UMIc	NGSReadsTreatment	Nubeam‐dedup	BioSeqZip	Fastp	FastUniq	pRESTO^a^	CD‐HIT‐DUP^a^	ParDRe^a^	Minirmd^a^
SRR1543964	38147	0	/	6562	0	0	0	0	/	{0}	{0}	{0}	{0}
SRR1543965	33993	2	/	3491	0	0	0	0	/	{0}	{0}	{0}	{0}
SRR1543966	30189	1	/	2634	0	0	0	0	/	{0}	{0}	{0}	{0}
SRR1543967	35759	0	/	3220	0	0	0	0	/	{0}	{0}	{0}	{0}
SRR1543968	30209	0	/	2647	0	0	0	0	/	{0}	{0}	{0}	{0}
SRR1543969	38918	1	/	8898	0	0	0	0	/	{0}	{0}	{0}	{0}
SRR1543970	28151	0	/	2535	0	0	0	0	/	{0}	{0}	{0}	{0}
SRR1543971	22909	1	/	2639	0	0	0	0	/	{0}	{0}	{0}	{0}
SRR28313972	2311229	3802	/	/	0	0	0	0	/	{0}	{0}	{0}	{0}
SRR28313990	143702	3993	/	/	0	0	0	0	/	{0}	{0}	{0}	{0}
SRR28314008	369470	0	/	/	0	0	0	0	/	{0}	{0}	{0}	{0}
SRR11207257	/	421653	6401766	/	0	0	0	10952692	0	{0}	{0}	{0}	{0}
	/	331566	6410184	/	0	0	0	11960912	0	{0}	{0}	{0}	{0}

*Note*: The symbol "/" signifies that this method does not support a single/pair‐end data set for PCR‐deduplication, or that it cannot produce results when executed on the corresponding data set.

^a^ These methods permit mismatches during PCR‐deduplication, and instances where their values are {0} indicate that these methods never introduce new sequences, achieved by setting mismatch numbers to {0, 1, 2, 3}.

On the 11 single‐end datasets, four solely‐computational methods NGSReadsTreatment, Nubeam‐dedup, BioSeqZip and Fastp have made the same or a quite similar unique‐read set as those obtained directly from the original data without applying any noise removal ideas (see Figure 2). Moreover, none of these methods introduced new sequences after PCR‐deduplication as shown in Table 3. These results indicate that these methods identified all the unique reads in the sequencing data as genuine biological sequences.

On the datasets SRR1543964‐SRR1543971, three UMI‐based PCR‐deduplication algorithms (UMI‐tools, AmpUMI and UMIc) resulted in an average decrease (by 88.61%, 82.97% and 88.69%, respectively) in the number of unique reads (see Figure 2A and Table S1). Note that UMI‐tools and UMIc introduced numerous new sequences that did not exist in the original datasets. Introducing new sequences by UMI‐tools is attributed to the reason that it performed the PCR‐deduplication and correction of sequencing data based on a reference genome. In detail, approximately 8.24%–20.27% of the unique sequences after the deduplication were found as new sequences, indicating that UMIc overcorrected PCR biases and errors across the eight datasets.

On the datasets SRR28313972, SRR28313990 and SRR28314008, the number of unique reads decreased by an average of 40.3% and 57.18% after deduplication with UMI‐tools and AmpUMI, respectively. Following deduplication, 11.7%–52.5% of the unique reads obtained with UMI‐tools were identified as new sequences. In contrast, only 0.15% and 0.3% of the unique reads were identified as new sequences by AmpUMI in datasets SRR28313972 and SRR28313990, respectively. UMIc was terminated or failed to yield valid deduplication results on these three datasets after running for an extended period of 1 month.

Furthermore, the solely‐computational tools ParDRe and Minirmd allowing mismatches exhibited a gradual decrease in the number of unique reads after PCR‐deduplication when the mismatch threshold increased from 0 to 3 (see Figure 2) and these methods did not generate any new sequences after PCR‐deduplication (see Table 3). The number of unique reads produced by CD‐HIT‐DUP fluctuates in response to changes in mismatches, but it does not exhibit consistent patterns across all datasets. These performances demonstrate that the mismatch‐allowed strategy by these methods can effectively eliminate some PCR biases and errors. Nevertheless, the number of unique reads is still relatively higher than the UMI‐based algorithms. Regarding pRESTO, the results as shown in Figure 2 and Table 3 suggest that it has failed to identify base errors through the mismatch strategy after PCR‐deduplication.

On the pair‐end dataset SRR11207257, UMI‐tools and UMIc encountered computing memory issues and could not complete the PCR‐deduplication process. AmpUMI and Calib, on the other hand, yielded unique reads that consisted entirely of new sequences not present in the original dataset (refer to Figure 2B and Table 3). The reason is that these methods utilized both read 1 (R1) and read 2 (R2) from opposite ends of a DNA fragment to reconstruct the complete fragment sequence.

All the solely computational PCR‐deduplication methods except Fastp did not result in any new sequences on the pair‐end dataset SRR11207257 during the PCR‐deduplication process. Instead, these methods identified and removed duplicate sequences, resulting in unique reads already present in the original dataset. In contrast, Fastp produced a substantial number of new sequences as shown in Table 3, which exceeded the count of unique reads in the original data set as indicated in Figure 2B. Notably, the new sequence is derived from altering the original sequence during deduplication. The number in Figure 2 and Table S1 represents the count of non‐repeating sequences rather than the total number. Put differently, following deduplication by Fastp, two or more sequences with distinct IDs but identical sequences have been transformed into different sequences. The result suggests that when reconstructing the entire fragment using both read 1 (R1) and read 2 (R2) of a DNA fragment, Fastp may tend to overcorrect the sequencing reads.

### 4.2 Comparison on the numbers of unique reads yielded by UMI‐based methods

**FIGURE 3:**
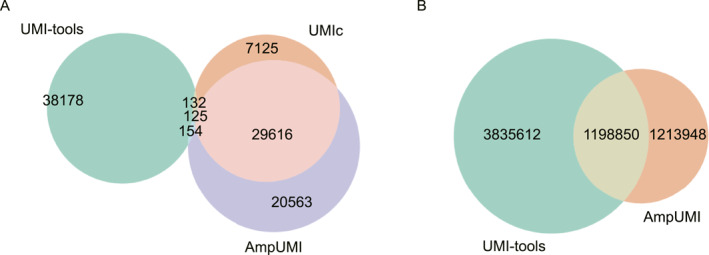
The performance comparison regarding the overlap and difference between unique read sets after PCR‐deduplication by UMI‐based methods. (A) Venn diagram illustrates the result between UMI‐tools [25], AmpUMI [36] and UMIc [35] on the dataset of SRR1543964. (B) Venn diagram illustrates the result between UMI‐tools [25] and AmpUMI [36] on the dataset of SRR28313972.

To compare the deduplicated sets of unique reads yielded by the three UMI‐based PCR‐deduplication methods AmpUMI, UMI‐tools and UMIc, we utilised Venn diagrams to visualise the overlap and differences among the unique read sets after PCR‐deduplication. These Venn diagrams are presented in Figures 3 and S1 for other datasets.

The Venn diagrams illustrate a significant overlap between UMIc‐ and AmpUMI‐deduplicated reads on the datasets SRR1543964‐SRR1543971. For instance, Figure 3A presents the Venn diagram when these three methods were applied to SRR1543964, where the areas of overlap between the circles represent the number of common sequences of the read sets. On the dataset SRR1543964, 80.05% of the unique reads identified by UMIc were also confirmed by AmpUMI. However, only 58.69% of the unique reads identified by AmpUMI were found by UMIc (Figure 3A). Across the eight datasets, UMIc and AmpUMI exhibit a mutual confirmation of 86.4% and 57.25% unique reads on average, as depicted in Figures 3A and S1. It is worth noting that UMIc introduced many new sequences after PCR‐deduplication, indicating a potential overcorrection of PCR biases and errors.

The unique reads identified by UMI‐tools and AmpUMI show significant overlap across datasets such as SRR28313972, SRR28313990 and SRR28314008 (see Figures 3B and S1). For example, in the dataset SRR28313972, 23.8% of the unique reads identified by UMI‐tools were also confirmed by AmpUMI, while AmpUMI found 49.7% of the unique reads identified by UMI‐tools (Figure 3B).

The intersections between UMI‐tools and UMIc and those between UMI‐tools and AmpUMI were extremely small on the datasets SRR1543964‐SRR1543971 and the intersections between UMI‐tools and AmpUMI varied considerably across different groups of datasets. These findings stem from UMI‐tools introducing numerous new sequences via alignment to a reference during its PCR‐deduplication process. Its performance is likely influenced by factors such as the sequencing samples and the reference genome.

We did not perform overlap and difference analysis on the paired‐end dataset SRR11207257. The reason is that the deduplicated reads consist entirely of new sequences and exhibit significant differences in counts, making comparative analysis meaningless from the perspective proposed in this article.

### 4.3 UMI‐based approaches in comparison to solely‐computational PCR‐deduplication methods

We benchmark the 9 solely‐computational methods against the UMI‐based ones on the 11 single‐end datasets. We calculated the numbers of unique reads in the intersections between the sets of unique sequences produced by UMI‐tools [25], AmpUMI [36] and UMIc [35] and those by the nine solely‐computational methods NGSReadsTreatment [41], Nubeam‐dedup [42], BioSeqZip [72], Fastp [71], FastUniq [73], pRESTO [47], CD‐HIT‐DUP [45], ParDRe [43] and Minirmd [18].

**FIGURE 4:**
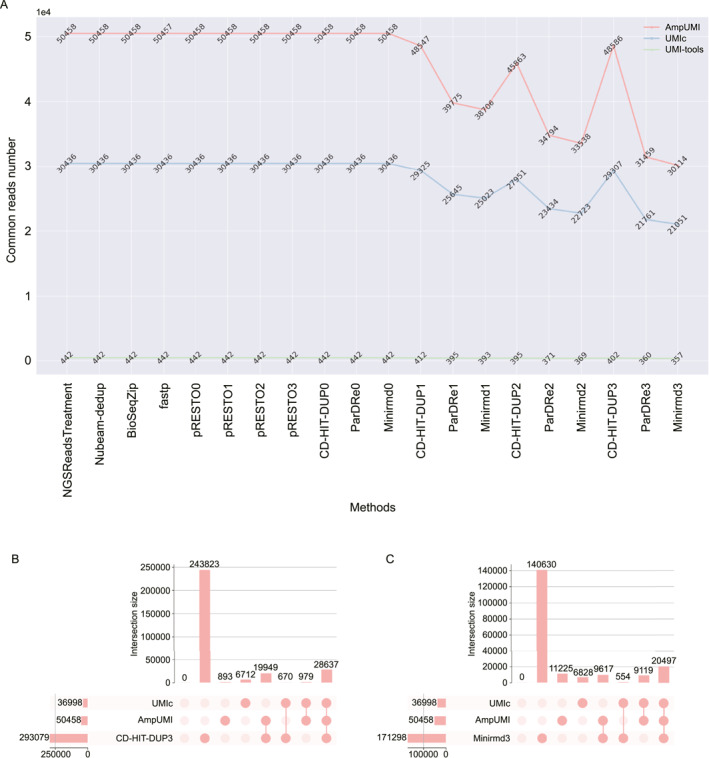
Overlap and differences of deduplicated unique reads between the computational tools and UMI‐based approaches on the data set SRR1543964. (A) Line chart comparing overlapped read numbers between each of the computational methods of NGSReadsTreatment [41], Nubeam‐dedup [42], BioSeqZip [72], Fastp [71], pRESTO [47], CD‐HIT‐DUP [45], ParDRe [43] and Minirmd [18] with each of the UMI‐based methods of UMI‐tools [25], AmpUMI [36] and UMIc [35] on the data set SRR1543964. (B–C) Upset plots for comparing the overlap and differences of deduplicated unique reads between the computational methods of CD‐HIT‐DUP [45] and Minirmd [18] and UMI‐based methods of AmpUMI [36] and UMIc [35] on the data set SRR1543964. The number immediately following a method indicates the mismatch values allowed by that method.

**FIGURE 5:**
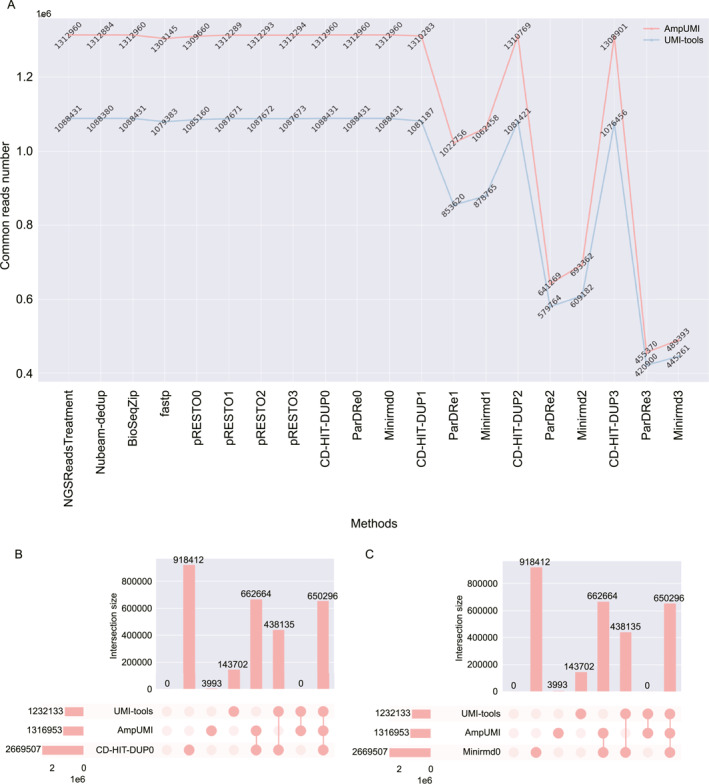
Overlap and differences of deduplicated unique reads between the computational tools and UMI‐based approaches on the data set SRR28313990. (A) Line chart comparing overlapped read numbers between each of the computational methods of NGSReadsTreatment [41], Nubeam‐dedup [42], BioSeqZip [72], Fastp [71], pRESTO [47], CD‐HIT‐DUP [45], ParDRe [43] and Minirmd [18] with each of the UMI‐based methods of UMI‐tools [25] and AmpUMI [36] on the data set SRR28313990. (B–C) Upset plots for comparing the overlap and differences of deduplicated unique reads between the computational methods of CD‐HIT‐DUP [45] and Minirmd [18] and UMI‐based methods of UMI‐tools [25] and AmpUMI [36] on the data set SRR28313990. The number immediately following a method indicates the mismatch values allowed by that method.

The line charts presented in Figures 4A and 5A compares the number of overlapped reads between the computational methods and UMI‐based methods on the datasets SRR1543964 and SRR28313990, respectively. Figures S2–S10 present the performance of the other datasets. The results illustrate that the unique reads obtained using AmpUMI are a subset of those obtained by the computational methods NGSReadsTreatment, Nubeam‐dedup, BioSeqZip, Fastp, FastUniq and pRESTO, as well as CD‐HIT‐DUP, ParDRe and Minirmd (without allowing any mismatches). All the sequences, except for those newly generated sequences after deduplication, are also present in the deduplicated set by the above computational methods. These observations are as expected because these solely computational approaches do not introduce new sequences while not correcting the PCR biases and errors. However, when mismatches (0–3) were allowed, the common sequences comparing UMI‐based tools with the methods CD‐HIT‐DUP, ParDRe, or Minirmd decreased, which suggests that CD‐HIT‐DUP, ParDRe and Minirmd turned some actual biological reads into wrong states due to over error correction in the PCR‐deduplication process. It is worth noting that the computational methods had a smaller amount of overlapping reads with UMI‐tools on the datasets SRR1543964‐SRR1543971 since UMI‐tools introduced many new sequences in the step of alignment to the reference genome.

We have utilised UpSet plots to visualize the intersections between the deduplicated set of the unique reads obtained from every computational method and that from the UMI‐based method AmpUMI and UMIc or UMI‐tools. The rows in these UpSet plots stand for the read sets produced by PCR‐deduplication methods and the columns stand for the intersections between these unique read sets. The highlighted cell or multiple cells connected with a line indicate the reading direction of an intersection. The size of the intersections is shown aligned with the rows, also as bar charts. The UpSet plots in Figures 4B,C and 5B,C depict the deduplicated intersections between CD‐HIT‐DUP or minirmd and the methods AmpUMI and UMIc or UMI‐tools on datasets SRR1543964 and SRR28313990, respectively. In the UpSet plots, where the column index begins at 0, superior performance in accurately identifying genuine reads is indicated by lower values in columns 1, 2 and 6 and higher values in columns 4, 5 and 7. Additionally, the proximity of the third column to the number of new sequences introduced by UMIc or UMI‐tools implies better performance.

**FIGURE 6:**
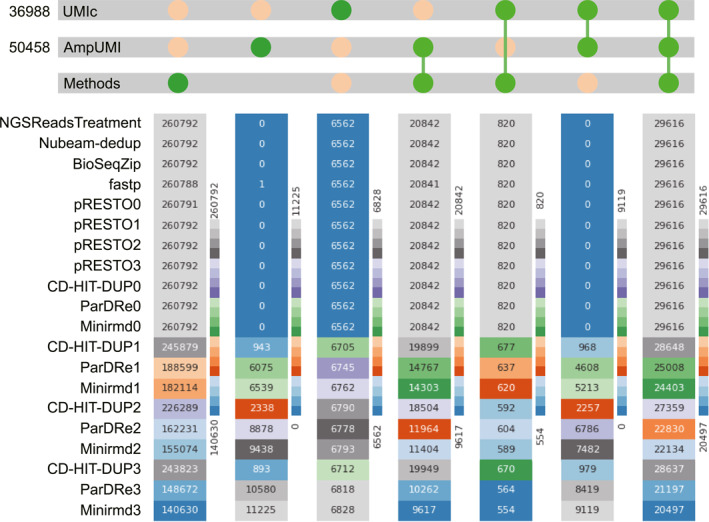
Heatmap for comparing overlapped reads number among each of the computational methods of NGSReadsTreatment [41], Nubeam‐dedup [42], BioSeqZip [72], Fastp [71], pRESTO [47], CD‐HIT‐DUP [45], ParDRe [43] and Minirmd [18] with the UMI‐based methods of AmpUMI [36] and UMIc [35] on the data set SRR1543964. The number immediately following a method indicates the mismatch values allowed by that method.

To facilitate the comparison of the performance of different methods, we use the heat map to compare the size of the seven intersections (columns 1–7 of the standard UpSet plot) of different methods and the results are shown in Figure 6 and Figures S11–S20 depict the results on the data sets SRR1543965‐SRR1543971, SRR28313972, SRR28313990 and SRR28314008. The results (Figure 6) in the first eleven rows of the heatmap indicate that these computational methods kept all the unique reads from the original datasets without correcting the PCR bias errors, except the methods Fastp lost one unique read. CD‐HIT‐DUP, ParDRe and Minirmd can eliminate some PCR biases and errors by allowing mismatches when PCR‐deduplication was processed (see the last nine rows of the heatmap in Figure 6). However, there are hundreds of thousands of distinct reads unique to these methods after PCR‐deduplication (first column of the heatmap), suggesting that these methods left hundreds of thousands of erroneous reads uncorrected. The numbers in the fourth, fifth and seventh columns decreased and the numbers in the second, third and sixth columns increased, demonstrating that these methods wrongly identified some biological reads as PCR errors.

### 4.4 Runtime and memory consumption

We compared the CPU time and the peak memory usage consumed by UMI‐tools [25], AmpUMI [36], UMIc [35], NGSReadsTreatment [41], Nubeam‐dedup [42], BioSeqZip [72], Fastp [71], pRESTO [47], CD‐HIT‐DUP [45], ParDRe [43] and Minirmd [18] on the datasets SRR1543964‐SRR1543971 (see Table 4). AmpUMI achieved the fastest speed among the UMI‐based methods and it was able to complete the deduplication in 1 minute with a memory usage of less than 550 megabytes (MB). The computational methods except for pRESTO, NGSReadsTreatment or ParDRe ran fast to complete in 1 minute with a peak memory ranging from 17 to 13329 MB. ParDRe was the slowest method that took up to more than 1 h to complete on the dataset SRR1543969.

**TABLE 4:** Runtime(T) and peak Memory(M) consumption by PCR‐deduplication methods of UMI‐tools [25], AmpUMI [36], UMIc [35], NGSReadsTreatment [41], Nubeam‐dedup [42], BioSeqZip [72], Fastp [71], pRESTO [47], CD‐HIT‐DUP [45], ParDRe [43] and Minirmd [18] on the datasets SRR1543964–SRR1543971.

Dataset	UMI‐tools		AmpUMI		UMIc		NGSReadsTreatment		Nubeam‐dedup		BioSeqZip		Fastp		pRESTO		CD‐HIT‐DUP		ParDRe		Minirmd	
	T	M	T	M	T	M	T	M	T	M	T	M	T	M	T	M	T	M	T	M	T	M
SRR1543964	81.6	3.2	0.4	476.4	670.3	5998.6	20.2	834.2	0.2	30.2	0.1	13166.2	0.1	4146.8	5.8	4207.3	0.1	870.1	53.9	2.7	1.0	1229.5
SRR1543965	81.2	3.3	0.2	402.2	619.5	4703.7	16.9	825.3	0.2	22.9	0.1	13089.7	0.1	4144.9	5.3	3669.5	0.1	755.5	38.5	2.7	0.5	1101.5
SRR1543966	81.8	3.3	0.2	479.5	657.5	5747.5	19.5	834.0	0.2	31.4	0.1	13226.6	0.1	4146.2	4.4	4645.8	0.1	933.4	51.3	2.7	1.0	1342.8
SRR1543967	82.8	3.2	0.3	509.0	794.5	6213.7	20.4	919.6	0.2	32.2	0.2	13329.1	0.1	4146.4	4.4	5346.1	0.1	1059.4	61.5	2.6	0.9	1495.2
SRR1543968	82.7	3.2	0.2	403.0	596.0	5586.8	13.2	833.1	0.2	28.6	0.1	13159.7	0.2	4146.4	3.6	4304.2	0.1	860.1	44.2	2.7	0.8	1256.3
SRR1543969	81.9	3.2	0.3	542.6	875.1	6245.7	23.8	203.0	0.2	33.6	0.2	13290.8	0.2	4144.7	4.3	5114.5	0.1	1048.2	63.9	2.6	1.0	1442.3
SRR1543970	81.6	3.2	0.2	452.3	599.9	5754.3	16.0	205.9	0.2	27.9	0.1	13222.8	0.1	4143.9	3.9	4625.3	0.1	919.0	48.6	2.4	0.9	1339.3
SRR1543971	80.3	3.1	0.2	307.4	415.7	4214.7	9.8	823.2	0.1	17.2	0.1	12941.6	0.1	4143.1	2.4	2897.5	0.1	567.7	21.3	2.4	0.4	907.8

*Note*: The CPU model of Intel(R) Xeon(R) Gold 6238R CPU@2.20 GHz was used by all the methods. Parallel‐capable methods use 64 threads or processes for parallelization. The runtime is given in minutes and memory consumption is given in MB.

In summary, the comparative analyses have revealed that: (1) AmpUMI is the best tool among different UMI‐based PCR‐deduplication methods as it runs fast and introduces the smallest number or almost does not introduce any new sequences after PCR‐deduplication; (2) the computational PCR‐deduplication methods, such as NGSReadsTreatment, Nubeam‐dedup, BioSeqZip, Fastp and pRESTO, always produce the same deduplicated results. However, these methods do not consider any PCR biases and errors and the results are the unique reads of the original data set. (3) The mismatch‐allowed computational PCR‐deduplication can eliminate some PCR biases and errors but could mistakenly identify genuine reads as near‐duplicates to be removed and still leave hundreds of thousands of erroneous reads uncorrected. The best mismatch‐allowed computational PCR‐deduplication approach is CD‐HIT‐DUP as it keeps more original reads after PCR‐deduplication.

### 4.5 Performance comparisons for different error‐correction methods

To compare the performance of the error‐correction methods, we investigated the number of changes of unique reads, the number of corrected reads with the corresponding percentage out of the total reads and the number of wrongly introduced new sequences after the correction. Table 5 presents the performance comparisons for the methods BFC [56], Bcool [68], Care [65, 66], Coral [61], Fiona [63], Lighter [49], Pollux [57] and RACER [54] on SRR1543964 and SRR28313972. The results on the other datasets are shown in Tables S2–S3.

**TABLE 5:** Summary of changes in unique reads, corrected reads, and erroneously introduced new reads after error correction using the error‐correction methods of BFC [56], Bcool [68], Care [65, 66], Coral [61], Fiona [63], Lighter [49], Pollux [57] and RACER [54]on the datasets SRR1543964 and SRR28313972, respectively.

Datasets	Methods	The number of unique reads			Corrected	Total	Percentage	New reads number
		Before correction	After correction	Decreased by	Reads number			
SRR1543964	Bcool	312070	264507	15.24%	76717	1100818	6.97%	20814
	Care		205233	34.23%	121111		11.00%	10149
	Coral		287856	7.76%	32177		2.92%	3111
	Fiona		201652	35.38%	392318		35.64%	127961
	Lighter		196024	37.19%	413016		37.52%	123780
	Pollux		269102	13.77%	154506		14.04%	27145
	RACER		262518	15.88%	971178		88.22%	250729
	BFC		310798	0.41%	116459		10.58%	105443
SRR28313972	Bcool	5504307	5172638	6.03%	476377	8894257	5.36%	46157
	Care		5237545	4.85%	661952		7.44%	364689
	Coral		5259309	4.45%	517190		5.81%	259121
	Fiona		4671112	15.14%	5385791		60.55%	3924897
	Lighter		5113556	7.10%	1447691		16.28%	872566
	Pollux		4718199	14.28%	2094747		23.55%	1088558
	RACER		5322682	3.30%	2840506		31.94%	2158500
	BFC		5501333	0.05%	900474		10.12%	815728

We observed a decrease in the number of unique reads for all of the methods after the error correction except for the methods RACER and BFC on the datasets SRR1543971 and SRR28313990. There is no relevant pattern in the percentage changes of unique reads in different data sets between different methods except for method BFC as it has the smallest change (e.g. only 0.41% on SRR1543964% and 0.05% on SRR28313972) in unique reads in each data set by comparing with other methods. For the other methods, Lighter made the largest reduction (a percentage decrease of 37.19%) on dataset SRR1543964, in contrast, Fiona made the largest reduction on dataset SRR28313972 (Table 5). On the other hand, some methods corrected a high percentage of reads. For example, RACER corrected a high percentage of reads (88.22%) on SRR1543964 while Fiona corrected 60.55% of reads on SRR28313972, suggesting that a significant proportion of 88.22% and 60.55% of the reads may have been wrongly identified as erroneous. Moreover, all of these methods generated tens to hundreds of thousands of non‐existing reads after error correction. This result implies that these methods introduced numerous new errors during the correction process. Error correction results on multiple data sets show that no method is the best and from the perspective of generating new sequences, Coral demonstrated the best performance on the datasets SRR1543964‐SRR1543971, while Bcool demonstrated the best performance on the group of datasets SRR28313972, SRR28313990 and SRR28314008, as they introduced the smallest number of new reads.

Furthermore, we analysed the overlaps and differences between the sets of unique reads obtained by different error‐correction algorithms using UpSet plots. Comparative results can be found in Figures S21–S31 for the datasets SRR1543964‐SRR1543971, SRR28313972, SRR28313990 and SRR28314008. We observed that these algorithms produced many sequences unique to each algorithm, ranging from 1636 to 1681595 sequences. In contrast, all the algorithms’ intersection sizes on the eight data sets ranged from 1527 to 2811 on datasets SRR1543964‐SRR1543971 and from 53451 to 602074 on the group of datasets SRR28313972, SRR28313990 and SRR28314008. The algorithm with the fewest unique sequences on SRR1543964‐SRR1543971 was Coral and on the group of datasets SRR28313972, SRR28313990 and SRR28314008 was Bcool. This observation aligns with their respective percentages of corrected reads and the numbers of newly generated sequences, as indicated in Table 5 and Tables S2–S3. The smaller size of the common intersections between the algorithms suggests that the results obtained from these error‐correction algorithms exhibit significant variation. In other words, employing various error‐correction methods will result in divergent conclusions during subsequent downstream analyses.

### 4.6 Runtime and memory consumption by different error‐correction methods

We also compared the CPU time and the peak memory usage required by the methods BFC [56], Bcool [68], Care [65, 66], Coral [61], Fiona [63], Lighter [49], Pollux [57] and RACER [54] on the datasets SRR1543964‐SRR1543971, as illustrated in Table 6. Lighter, BFC, Care and RACER were able to correct errors in a minute with less memory, while Coral could complete in less than 10 min, but consumed much more memory up to 128.6 gigabytes (GB). Pollux ran slowest with time up to 26.4 h. One of the reasons is that Pollux cannot run in parallel.

### 4.7 How error‐correction methods affect PCR‐deduplication

We conducted duplicate removal using solely‐computational PCR‐deduplication methods on the error‐corrected datasets from SRR1543964‐SRR1543971, SRR28313972, SRR28313990 and SRR28314008 to investigate how error‐correction methods affect PCR‐deduplication. In detail, each of these datasets was firstly corrected by BFC [56], Bcool [68], Care [65, 66], Coral [61], Fiona [63], Lighter [49], Pollux [57] and RACER [54]. Then we removed duplicates using the three computational methods CD‐HIT‐DUP [45], ParDRe [43] and Minirmd [18] when the mismatch threshold was set differently.

The line charts in Figure 7, Figure 8 and Figures S32–S62 illustrate the intersection size of the sets of the deduplicated reads after error correction in comparison with the corresponding sets directly obtained through UMI‐based PCR‐deduplication methods. Three solid lines, representing CD‐HIT‐DUP, ParDRe and Minirmd (with a permissible mismatch of not bigger than 3 bases), are contrasted with a dashed line depicting CD‐HIT‐DUP with no mismatch allowance.

**FIGURE 7:**
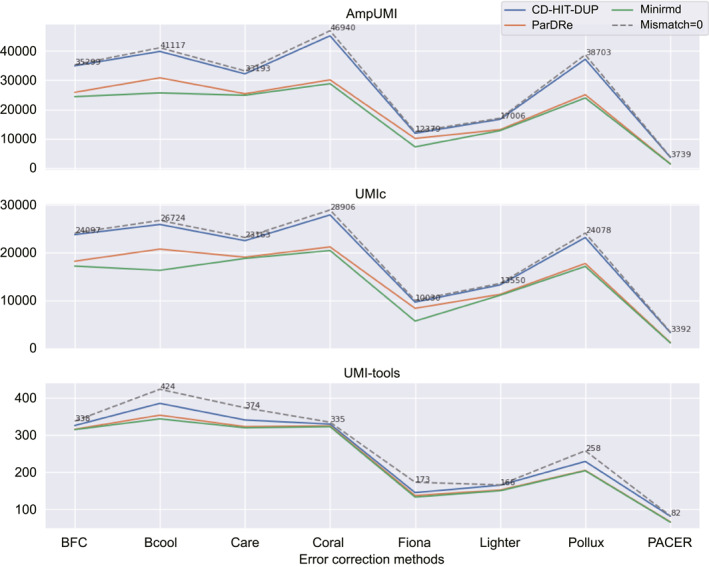
Line charts comparing the overlapped read numbers in the deduplicated read set by the PCR‐deduplication methods of CD‐HIT‐DUP [45], ParDRe [43] and Minirmd [18] on error‐corrected dataset SRR1543964, with each UMI‐based PCR‐deduplication methods of UMI‐tools [25], AmpUMI [36] and UMIc [35] on dataset SRR1543964. Error correction was performed using error‐correction methods BFC [56], Bcool [68], Care [65, 66], Coral [61], Fiona [63], Lighter [49], Pollux [57] and RACER [54], respectively. CD‐HIT‐DUP, ParDRe, and Minirmd employed a mismatched number set to 3. The dashed line labelled ‘Mismatch = 0’ represents results obtained by CD‐HIT‐DUP with a mismatch setting of 0.

**FIGURE 8:**
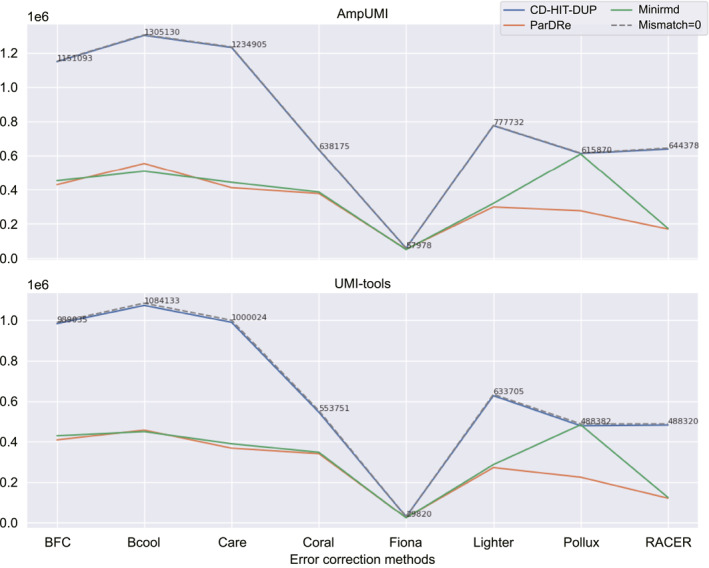
Line charts comparing the overlapped read numbers in the deduplicated read set by the PCR‐deduplication methods of CD‐HIT‐DUP [45], ParDRe [43] and Minirmd [18] on error‐corrected dataset SRR28313990, with each UMI‐based PCR‐deduplication methods of AmpUMI [36] and UMI‐tools [25] on dataset SRR28313990. Error correction was performed using error‐correction methods of BFC [56], Bcool [68], Care [65, 66], Coral [61], Fiona [63], Lighter [49], Pollux [57] and RACER [54], respectively. CD‐HIT‐DUP, ParDRe, and Minirmd employed a mismatched number set to 3. The dashed line labelled ‘Mismatch = 0’ represents results obtained by CD‐HIT‐DUP with a mismatch setting of 0.

Comparing the intersection size of unique reads after the PCR‐deduplication process applied to error‐corrected reads (Figures 7 and 8) with those of the directly deduplicated reads (Figures 4A and 5A), decrease in the number is observed on different group of datasets. This decrease suggests that the error corrections erroneously identified some authentic biological sequences as PCR errors. Similar outcomes observed across other datasets, as depicted in Figures S32–S62, indicate that the current error‐correction methods are not advantageous for PCR‐deduplication. In other words, the employment of the existing error‐correction methods appears to be an unnecessary step before the PCR‐deduplication process.

Moreover, most importantly, the size of both the directly deduplicated read set by computational methods and the deduplicated read set after error correction is significantly larger than that of the set of the deduplicated reads generated by UMI‐based PCR‐deduplication methods. For instance, in the dataset SRR1543964, the directly deduplicated read set by CD‐HIT‐DUP (with 2 mismatches) consists of 272,744 unique reads (Table S1), while the Coral error‐corrected read set comprises 287,856 unique reads (Table 5). However, the AmpUMI‐deduplicated read set only contains 50,458 unique reads (Table S1). Similar trends are observed in the other datasets for these PCR‐deduplication and error‐correction methods. This analysis demonstrates that both existing PCR‐deduplication and error‐correction methods leave a considerable number of erroneous reads untouched while generating a considerable number of new sequences by error‐correction approaches.

**TABLE 6:** Runtime(T) and peak Memory(M) consumption by error‐correction methods of BFC [56], Bcool [68], Care [65, 66], Coral [61], Fiona [63], Lighter [49], Pollux [57] and RACER [54] on the datasets SRR1543964‐SRR1543971.

Dataset	BFC		Bcool		Care		Coral		Fiona		Lighter		Pollux		RACER	
	T	M	T	M	T	M	T	M	T	M	T	M	T	M	T	M
SRR1543964	0.3	3.2	30.3	14.8	0.4	815.7	7.0	124136.4	84.1	2117.7	0.1	647.7	1263.2	1053.1	3.9	144.8
SRR1543965	0.3	3.1	20.8	14.4	0.4	803.2	5.9	128940.7	67.4	1889.3	0.1	647.7	1106.8	1053.2	2.8	122.5
SRR1543966	0.3	3.0	25.3	14.5	0.4	924.7	7.1	126143.5	96.1	2200.6	0.1	645.2	1329.0	1053.1	3.0	156.0
SRR1543967	0.3	3.2	24.8	14.9	0.4	1030.8	9.1	108028.4	101.2	2155.0	0.1	649.4	1570.3	1053.2	4.1	178.2
SRR1543968	0.3	3.2	17.8	14.4	0.4	840.3	6.4	123999.8	86.5	2119.7	0.1	649.5	1276.9	1039.6	2.9	149.3
SRR1543969	0.3	3.1	35.4	13.1	0.5	991.9	8.9	124624.7	106.1	2264.0	0.1	647.7	1583.1	1119.8	4.0	172.3
SRR1543970	0.3	3.1	19.6	13.6	0.4	895.7	7.7	122601.4	91.3	2191.1	0.1	649.6	1306.5	1053.1	3.1	156.7
SRR1543971	0.2	3.2	14.3	12.9	0.3	606.3	5.1	128633.8	60.3	1710.5	0.1	649.5	782.8	585.1	2.2	100.1

*Note*: The CPU model of Intel(R) Xeon(R) Gold 6238R CPU @ 2.20 GHz was used by all the methods. Parallel‐capable methods use 64 threads or processes for parallelization. The runtime is given in minutes and memory consumption is given in MB.

Furthermore, while error‐correction techniques offer only marginal benefits to PCR‐deduplication, the effectiveness varies among different methods. In the datasets SRR1543964‐SRR1543971, the Coral method yielded the highest number of overlapped reads with both the AmpUMI and UMIc methods (refer to Figures 7 and S32–S54); in contrast, RACER resulted in the smallest size of the corresponding intersections. On the datasets SRR28313972, SRR28313990 and SRR28314008, Bcool yielded the highest number of overlapped reads with both of the AmpUMI and UMIc methods (refer to Figure 8 and S55–S62) while Fiona resulted in the smallest size of the corresponding intersections.

In summary, these comparative studies have revealed that: (1) All error‐correction methods introduce many non‐existent sequences after error correction and leave many erroneous reads untouched. (2) No error‐correction method works best across all evaluation datasets, which aligns with the conclusion reported by the benchmarking study [28] that method performance varies significantly across different dataset types, with no single method consistently outperforming others. Coral and Bcool introduce the fewest new sequences and exhibit the largest overlap of unique reads with the UMI‐based deduplicated read set on the two different groups of datasets.

## 5 DISCUSSION

This study conducted a comprehensive investigation on both PCR‐deduplication and error‐correction, exploring the impact of error correction on PCR‐deduplication as well as comparing the performance of these state‐of‐the‐art methods from novel perspectives based on UMI datasets of short reads. We have uncovered notable limitations within existing algorithms working without UMI sequence data. The reads deduplicated solely through computational PCR‐deduplication and error‐correction methods display significant disparities when compared to those generated by UMI‐based techniques. This observation aligns with the established conclusion in the literature [37] that PCR duplicate removal without UMI sequence data tends to be overly aggressive.

While incorporating UMIs proves to be an effective strategy for mitigating data uncertainty brought up by PCR biases and errors, it is crucial to acknowledge significant practical challenges associated with their use. Firstly, PCR biases and errors may still occur within UMIs, posing a challenge for existing methods to differentiate between PCR duplicates and genuine biological sequences, particularly when PCR errors arise during the early‐cycle PCR process. Similarly, the identification and resolution of errors in the sequence during the initial PCR cycles present a complex task for existing error‐correction methods. One previous experimental analysis presented in the literature [37] has demonstrated that the prevalence of PCR duplicates is primarily determined by the quantity of initial materials and sequencing depth and there is no additional impact attributed to the cycles of PCR amplification. Secondly, UMI collisions may occur during library preparation, especially in large‐scale sequencing when short‐length UMIs are used. Most existing PCR‐deduplication methods are not equipped to handle UMI collisions and a recent method called DAUMI [26] has been developed to handle UMI collisions. Thirdly, UMI‐based strategies are characterized by their time‐consuming and expensive nature, rendering them less practical for large‐scale applications.

In addition, alignment‐based PCR‐deduplication methods may unintentionally remove genuine biological sequences, as they depend on reference genomes or transcriptomes to correct PCR errors. However, these references are based on implicit assumptions. For example, substantial evidence reveals the presence of errors, noise and biases in de novo transcriptome assemblies [10]. As alignment with the reference genome is a widely used strategy in bioinformatics, this survey study does not cover some in‐depth benchmarking analysis regarding alignment‐based PCR deduplication, which can be investigated as future work. For example, alignment with the reference genome and self‐alignment, which have severe impacts on deleting actual biological sequences, still need investigation. Future work can also include quantitative analysis on the proportion of real sequences mistakenly deleted by alignment‐based PCR deduplication methods and the number of variant bases compared to the reference genome.

Moreover, even though NGS platforms (e.g. Illumina instruments) share some standard techniques, their specific sequencing methods differ from each other, leading to reads with different error profiles [23, 74]. These error profiles may also vary depending on the sequencing task. For instance, the sequencing error profiles of miRNA sequencing data may differ from whole‐genome sequencing data or synthetic sequencing data. Therefore, further and more investigation and analysis are necessary to uncover and understand the underlying causes of PCR bias and sequencing errors. Furthermore, it remains unclear whether there exists a means to discern whether errors in a sequence stem from PCR or fluorophore crosstalk in the PCR‐deduplication and error‐correction processes. Future studies could delve into these issues more comprehensively, as their resolution holds significant implications for sequencing library preparation and sequencing technology.

Addressing these challenges can enhance the accuracy and reliability of downstream analyses and enable a more robust interpretation of NGS data. An ideal sequencing error removal algorithm should accurately restore erroneous reads to their correct states without introducing new errors or sequences. Here, we make suggestions and outline future research directions to improve existing computational methods aimed at reducing PCR biases and sequencing errors in short‐read data.

Firstly, current PCR‐deduplication and error‐correction methods can be improved by incorporating iterative and screening mechanisms to prevent the generation of non‐existent sequences. Existing mismatch‐allowed PCR‐deduplication methods remove near‐duplicates to eliminate PCR biases and errors. However, experimental evidence shows that these methods mistakenly identify some genuine identical reads as near‐duplicates to be removed. Enhancing their performance may be achieved by introducing additional information and parameters, such as sequence frequency, to filter out true near‐duplicates accurately. As an example, the method miREC [70] specifically designed to address errors in miRNA sequencing data has successfully prevented the introduction of new sequences after error correction by introducing an additional checking step.

Secondly, future research could explore the integration and enhancement of PCR‐deduplication tools and error‐correction methods. PCR‐deduplication techniques might be advanced to record sequencing IDs or track accumulated counts for each unique read while removing duplicates, thus preserving both read accuracy and abundance. Conversely, error‐correction methods could be refined to produce unique reads post‐correction, facilitating their incorporation into PCR‐deduplication workflows. Notably, methods like Calib [27] and DAUMI [26] have demonstrated success in recording sequencing IDs and tracking read abundance post‐deduplication.

Thirdly, many current computational methods for PCR‐deduplication and error‐correction strategies rely on *k*‐mer‐based, MSA‐based, DBG, hashing or minimiser techniques to establish relationships among generated sequences. However, these approaches often fail to address the fundamental causes of PCR and sequencing biases and errors. To overcome this limitation, the introduction of new mechanisms is crucial. Understanding the intricacies of PCR and fluorophore crosstalk error mechanisms, coupled with the incorporation of machine learning and deep learning techniques, may provide more effective solutions. An illustration of this need is evident in a study [16] where the predominant factor influencing PCR bias was identified as the relationship between library complexity and sequencing depth. Amplification noise, along with its correlation with the number of PCR cycles, was found to play a secondary role in contributing to this artifact. Recognizing and incorporating these insights can be instrumental in identifying inherent error patterns specific to each sequencing platform used for sequencing particular biomolecular samples.

## 6 CONCLUSION

NGS data have become increasingly prevalent in various bioinformatics applications in recent years. However, addressing the PCR and base calling errors is a long‐standing problem and is crucial for any downstream analysis whenever NGS short read is involved. PCR‐deduplication and error‐correction tools utilise computational techniques to address biases and errors introduced during short‐read sequencing. We have provided a comprehensive review of the existing PCR‐deduplication and error‐correction methods by assessing the performance of these algorithms from novel perspectives.

This study identified the limitations and deficiencies of existing PCR‐deduplication and error‐correction methods and proposed potential solutions from a broader perspective. The comparative analyses have revealed that the existing computational‐based PCR‐deduplication and error‐correction methods can eliminate some PCR and sequencing errors but still leave hundreds of thousands of erroneous reads uncorrected. Most solely‐computational PCR‐deduplication methods do not address bias and error removal and mismatch‐allowed approaches can remove some PCR errors but also delete some genuine identical reads as near‐duplicates or convert erroneous reads into different error states instead of their correct or normal states. All error‐correction methods introduce tens of thousands of new sequences after error restoration and the unique read sets obtained from different error‐correction methods show limited overlap, as these methods treat and correct just varying percentages of erroneous reads. Employing the existing error‐correction methods appears to be unnecessary as a preprocessing step for the PCR‐deduplication process.

## AUTHOR CONTRIBUTIONS

**Pengyao Ping**: Conceptualization; data curation; formal analysis; investigation; resources; validation; visualization; writing—original draft preparation; writing—review and editing. **Tian Lan**: Investigation; writing—review and editing. **Shuquan Su**: Investigation; writing—review and editing. **Wei Liu**: Supervision; resources; writing—review and editing. **Jinyan Li**: Conceptualization; project administration; funding acquisition; resources; supervision; writing—review and editing.

## CONFLICT OF INTEREST STATEMENT

The authors declare that they have no conflict of interest.

## ETHICS STATEMENT

This review article does not involve any research related to human or animal subjects.

## Supporting information

Supporting Information S1

## Data Availability

The 11 UMI‐based single‐end sequencing datasets of the accession IDs SRR1543964‐SRR1543971, SRR28313972, SRR28313990 and SRR28314008 together with a pair‐end sequencing dataset of the accession ID SRR11207257 were downloaded from SRA archive. The Python scripts for comparative analysis in this study are open source and publicly available at the GitHub repository Deduplication_ErrorCorrection.
