# Supplementary material for: How error correction affects polymerase chain reaction deduplication: A survey based on unique molecular identifier datasets of short reads
Source: Quant Biol. 2025 Mar 23;13(3):e99. doi: 10.1002/qub2.99 (PMC12806096; doi:10.1002/qub2.99)
Supplement: Supplementary file 1 — Supporting Information S1 [file QUB2-13-e99-s001.pdf]

Supplementary information, including Figures S1-S62 and Tables S1-S3, are extended data supporting the analysis in this review.

1 Supplementary Figures

- Figure S1 compares the overlap and differences between unique read sets after deduplication by UMI-based methods of UMI-tools, AmpUMI and UMIc using Venn diagrams on the data sets of SRR1543965-SRR1543971, SRR28313990 and SRR28314008.

- Figures S2-S10 are line charts for comparing overlapped reads numbers between each of the computational methods of NGSReadsTreatment, Nubeam-dedup, BioSeqZip, fastp, FastUniq, pRESTO, CD-HIT-DUP, ParDRe and Minirmd with each of the UMI-based methods of UMI-tools, AmpUMI and UMIc on the datasets of SRR1543965-SRR1543971, SRR28313990 and SRR28314008.

- Figures S11-S20 are heatmaps for comparing overlapped reads numbers among each of the computational methods of NGSReadsTreatment, Nubeam-dedup, BioSeqZip, fastp, FastUniq, pRESTO, CD-HIT-DUP, ParDRe and Minirmd with the UMI-based methods of AmpUMI and UMIc on the data sets of SRR1543965-SRR1543971, SRR28313972, SRR28313990 and SRR28314008. The number immediately following a method indicates the mismatch values allowed by that method.

- Figures S21-S31 compare the overlaps and differences between the unique read sets obtained by different error correction algorithms using UpSet plots on data sets SRR1543964-SRR1543971, SRR28313972, SRR28313990 and SRR28314008. These figures are long-scale pictures and their high-resolution versions are presented in the attachment separately in the form of png.

- Figures S32-S62 are line charts for comparing the number of overlapped reads between deduplicated read set by CD-HIT-DUP, ParDRe and Minirmd with mismatches ranging from 1 to 3 on error-corrected dataset SRR1543964-SRR1543971, SRR28313972, SRR28313990 and SRR28314008 with deduplicated read set by each of the UMI-based methods of AmpUMI, UMI-tools or UMIc. BFC, Bcool, Care, Coral, Fiona, Lighter, Pollux and RACER were used for error correction, respectively. The dashed line labelled "Mismatch=0" was obtained by CD-HIT-DUP with setting mismatch as 0.

2 Supplementary Tables

Table S1 summarises the comparative analysis regarding the number of the unique reads after PCR-deduplication by the algorithms UMI-tools, AmpUMI, Calib, UMIc, NGSReadsTreatment, Nubeam-dedup, BioSeqZip, fastp, FastUniq, pRESTO, CD-HIT-DUP, ParDRe and Minirmd on datasets SRR1543964-SRR1543971, SRR28313990 and SRR28314008. Tables S2-S3 illustrate Summary of changes in unique reads, corrected reads, and erroneously introduced new reads after error correction using the error-correction methods of BFC, Bcool, Care, Coral, Fiona, Lighter, Pollux and RACER on the data sets of SRR1543965-SRR1543971, SRR28313990 and SRR28314008.

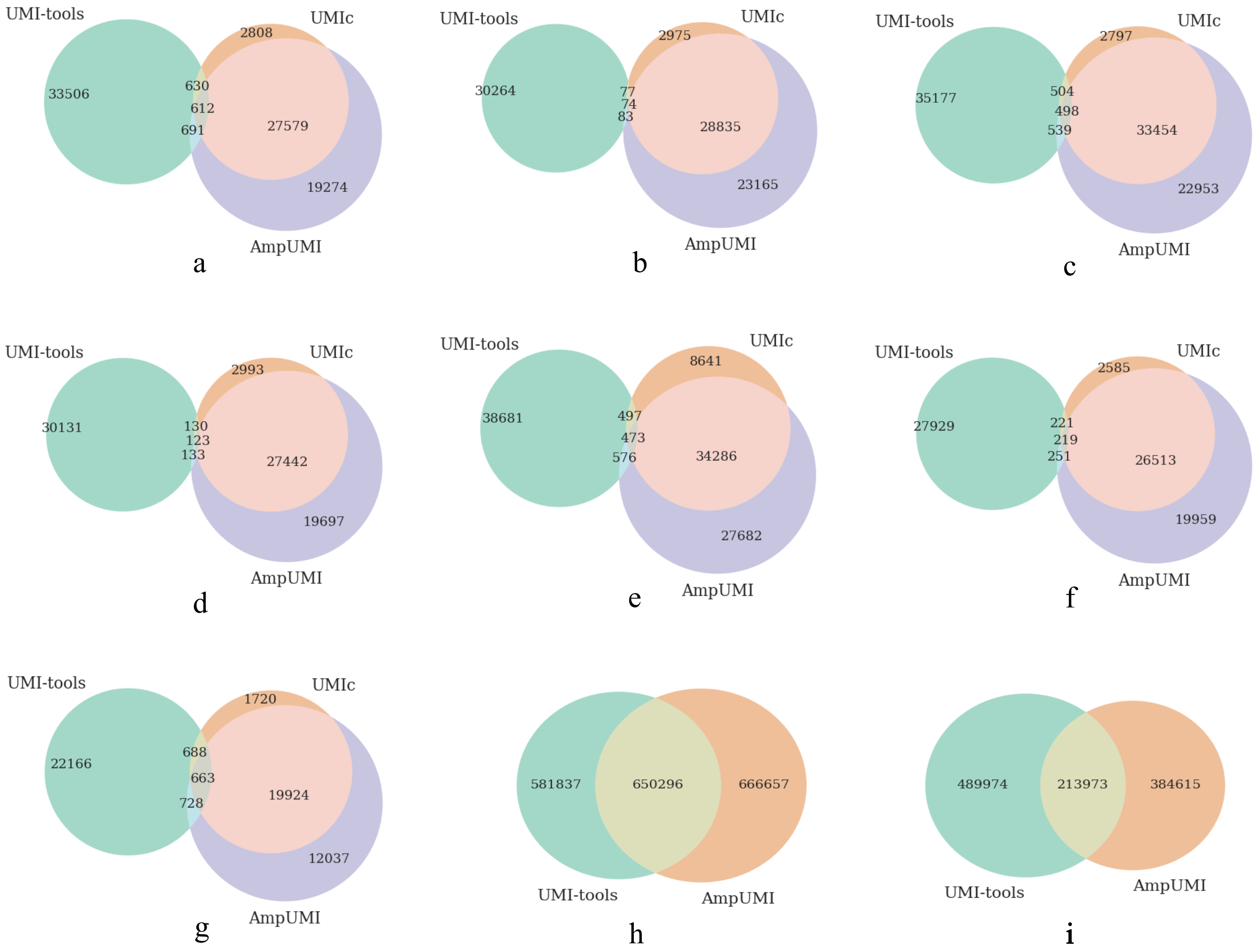

Figure S1: The performance comparison of the overlap and differences between unique read sets after deduplication by UMI-based methods of UMI-tools, AmpUMI and UMIc using Venn diagrams on the data sets of SRR1543965-SRR1543971, SRR28313990 and SRR28314008.

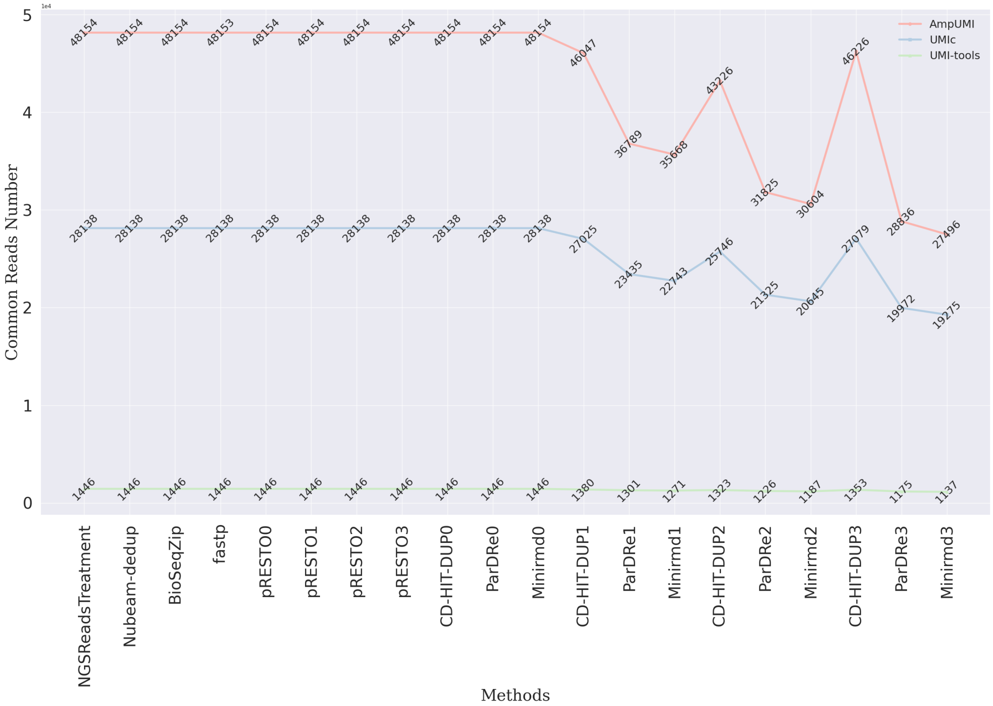

Figure S2: Line chart for comparing overlapped reads number between each of the computational methods of NGSReadsTreatment, Nubeam-dedup, BioSeqZip, fastp, FastUniq, pRESTO, CD-HIT-DUP, ParDRe and Minirmd with each of the UMI-based methods of UMI-tools, AmpUMI and UMIc on the data set SRR1543965.

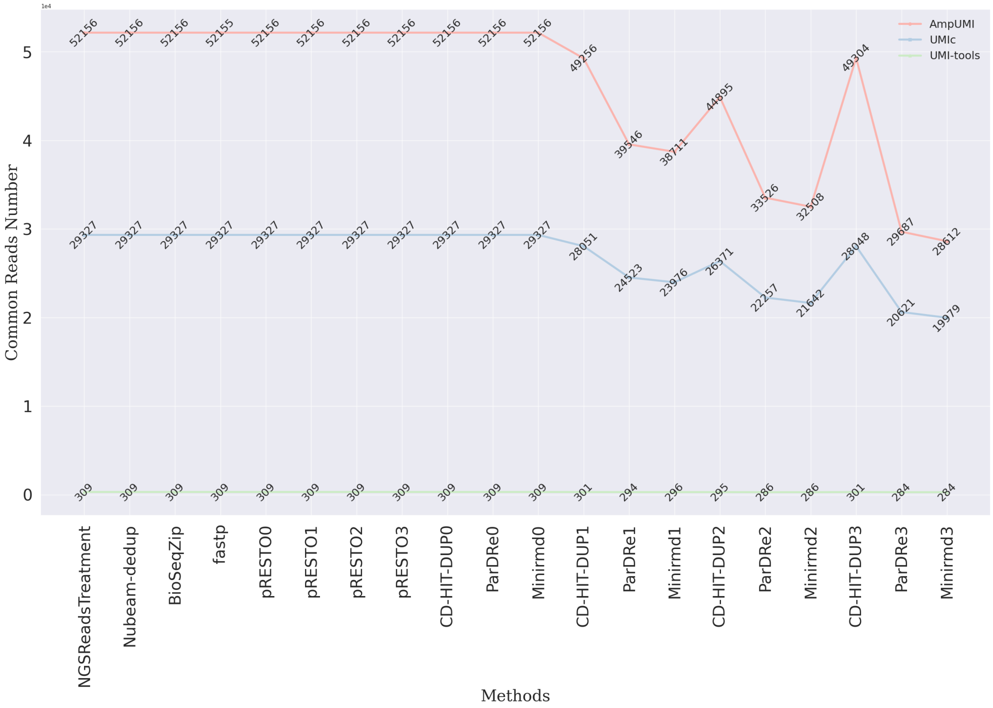

Figure S3: Line chart for comparing overlapped reads number between each of the computational methods of NGSReadsTreatment, Nubeam-dedup, BioSeqZip, fastp, FastUniq, pRESTO, CD-HIT-DUP, ParDRe and Minirmd with each of the UMI-based methods of UMI-tools, AmpUMI and UMIc on the data set SRR1543966.

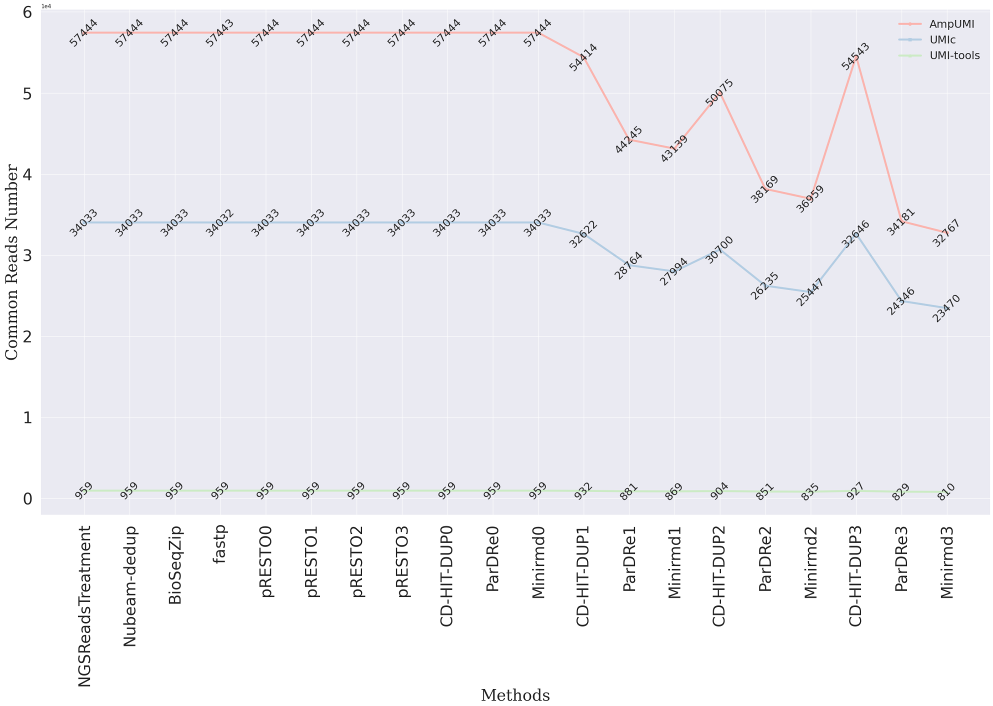

Figure S4: Line chart for comparing overlapped reads number between each of the computational methods of NGSReadsTreatment, Nubeam-dedup, BioSeqZip, fastp, FastUniq, pRESTO, CD-HIT-DUP, ParDRe and Minirmd with each of the UMI-based methods of UMI-tools, AmpUMI and UMIc on the data set SRR1543967.

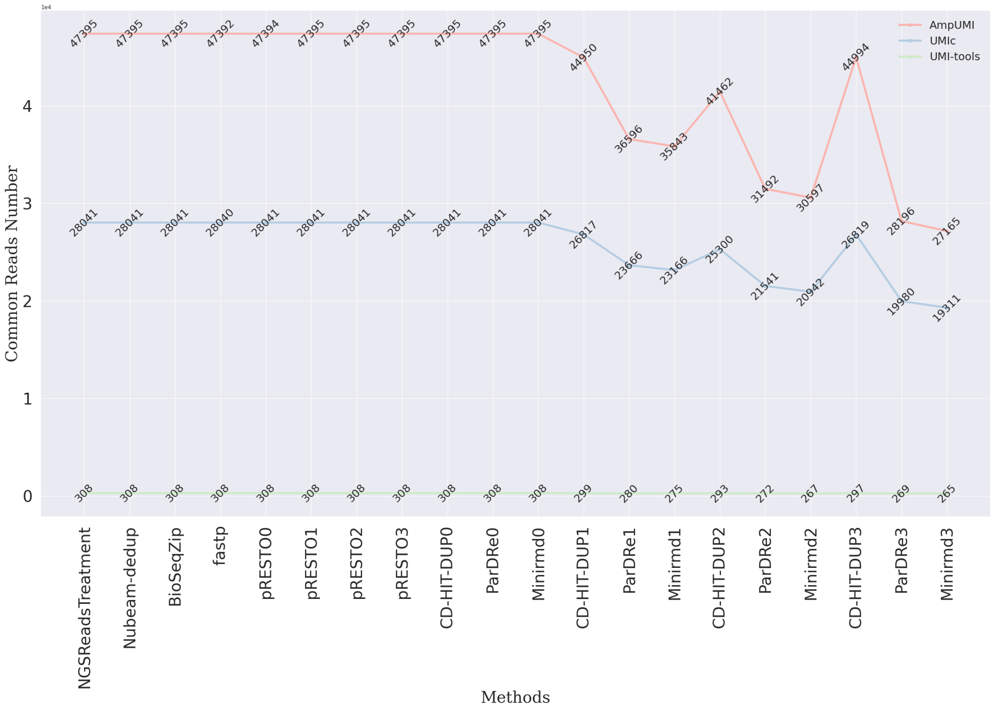

Figure S5: Line chart for comparing overlapped reads number between each of the computational methods of NGSReadsTreatment, Nubeam-dedup, BioSeqZip, fastp, FastUniq, pRESTO, CD-HIT-DUP, ParDRe and Minirmd with each of the UMI-based methods of UMI-tools, AmpUMI and UMIc on the data set SRR1543968.

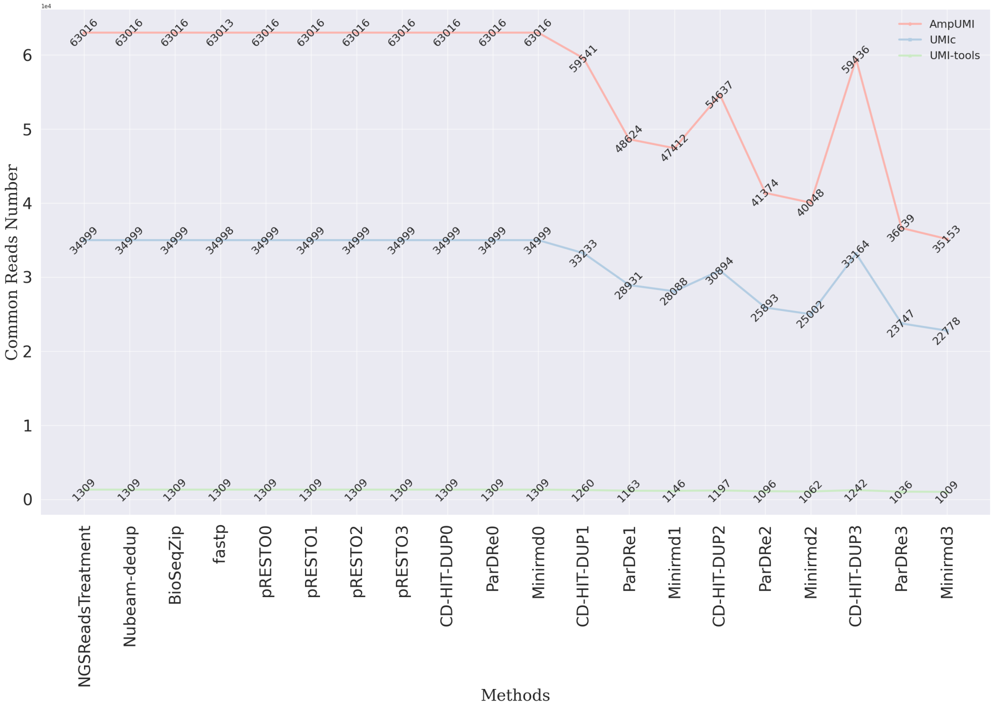

Figure S6: Line chart for comparing overlapped reads number between each of the computational methods of NGSReadsTreatment, Nubeam-dedup, BioSeqZip, fastp, FastUniq, pRESTO, CD-HIT-DUP, ParDRe and Minirmd with each of the UMI-based methods of UMI-tools, AmpUMI and UMIc on the data set SRR1543969.

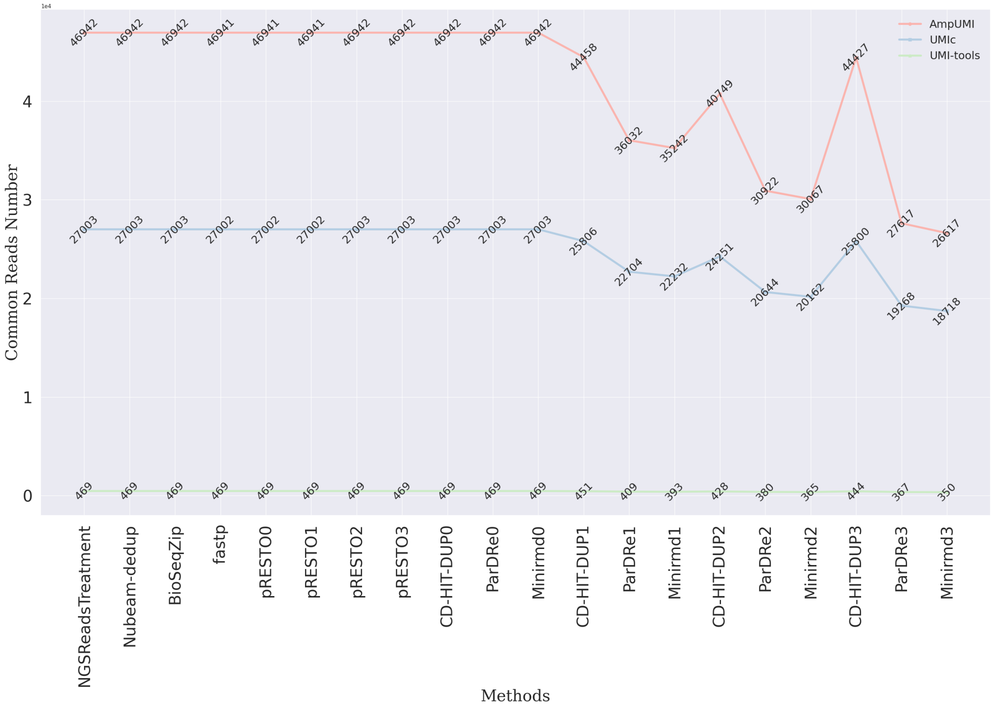

Figure S7: Line chart for comparing overlapped reads number between each of the computational methods of NGSReadsTreatment, Nubeam-dedup, BioSeqZip, fastp, FastUniq, pRESTO, CD-HIT-DUP, ParDRe and Minirmd with each of the UMI-based methods of UMI-tools, AmpUMI and UMIc on the data set SRR1543970.

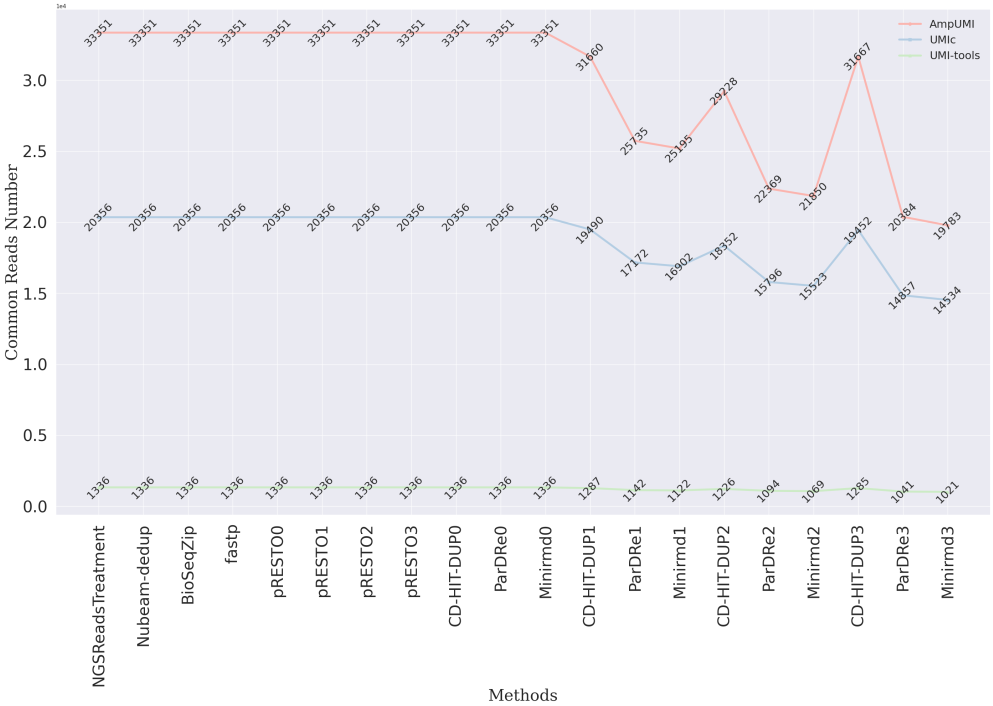

Figure S8: Line chart for comparing overlapped reads number between each of the computational methods of NGSReadsTreatment, Nubeam-dedup, BioSeqZip, fastp, FastUniq, pRESTO, CD-HIT-DUP, ParDRe and Minirmd with each of the UMI-based methods of UMI-tools, AmpUMI and UMIc on the data set SRR1543971.

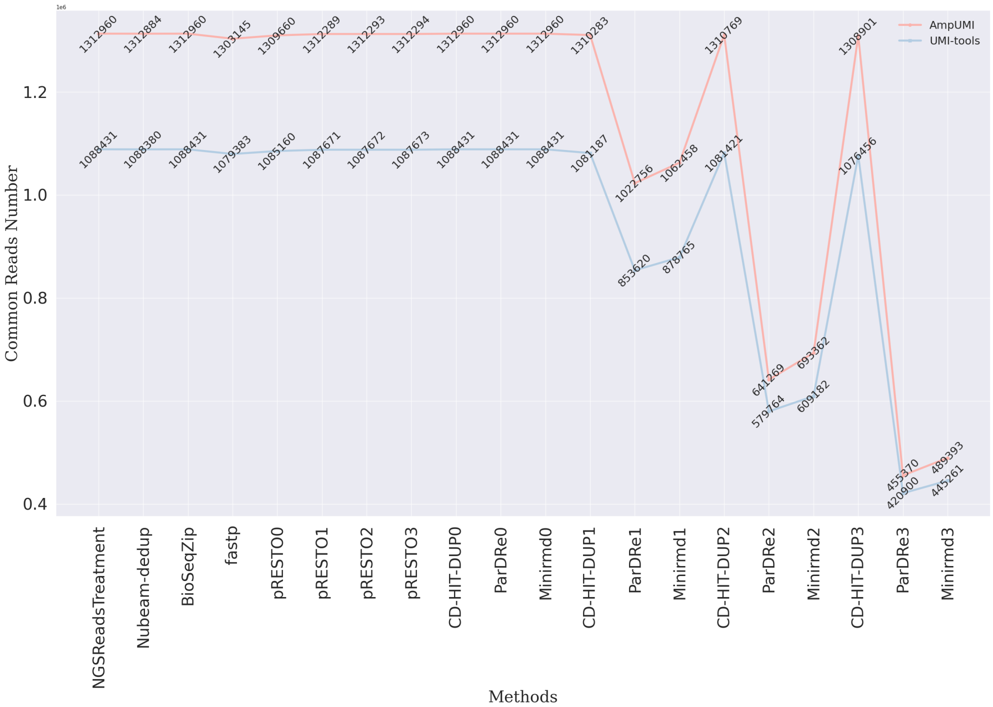

Figure S9: Line chart for comparing overlapped reads number between each of the computational methods of NGSReadsTreatment, Nubeam-dedup, BioSeqZip, fastp, FastUniq, pRESTO, CD-HIT-DUP, ParDRe and Minirmd with each of the UMI-based methods of UMI-tools and AmpUMI on the dataset SRR28313990.

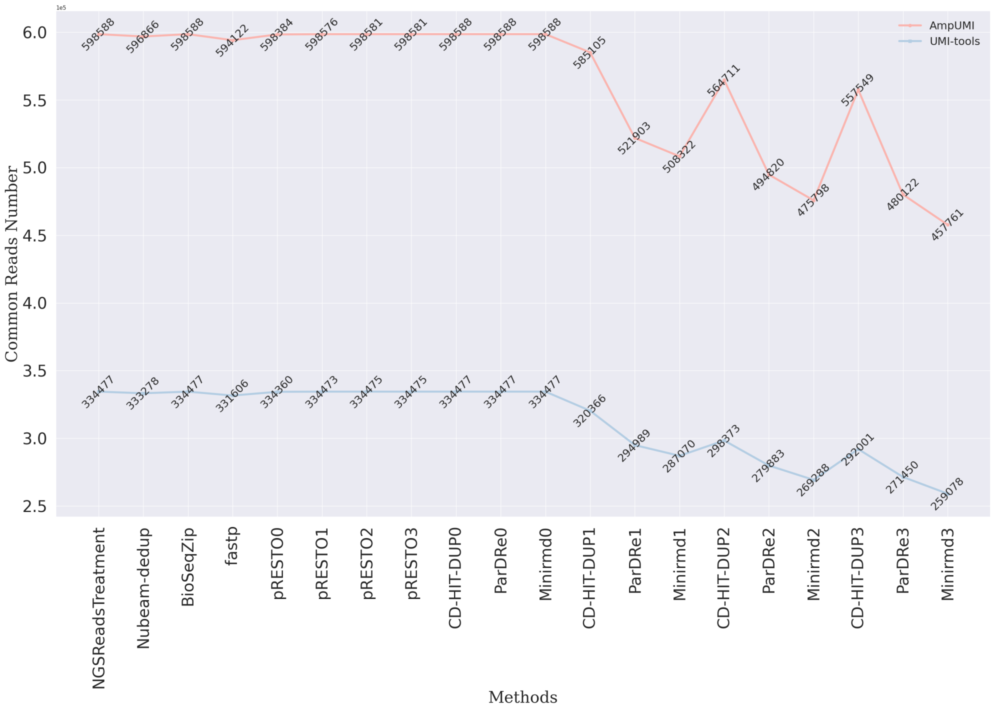

Figure S10: Line chart for comparing overlapped reads number between each of the computational methods of NGSReadsTreatment, Nubeam-dedup, BioSeqZip, fastp, FastUniq, pRESTO, CD-HIT-DUP, ParDRe and Minirmd with each of the UMI-based methods of UMI-tools and AmpUMI on the dataset SRR28314008.

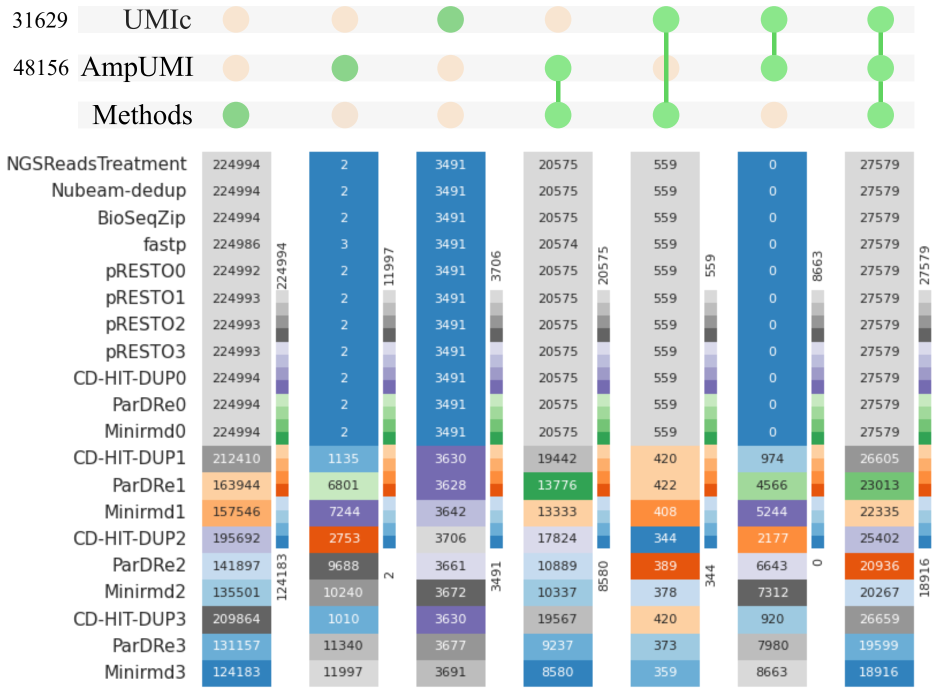

Figure S11: Heatmap for comparing overlapped reads number by each of the computational methods of NGSReadsTreatment, Nubeam-dedup, BioSeqZip, fastp, FastUniq, pRESTO, CD-HIT-DUP, ParDRe and Minirmd with the UMI-based methods of AmpUMI and UMIc on the data set SRR1543965.

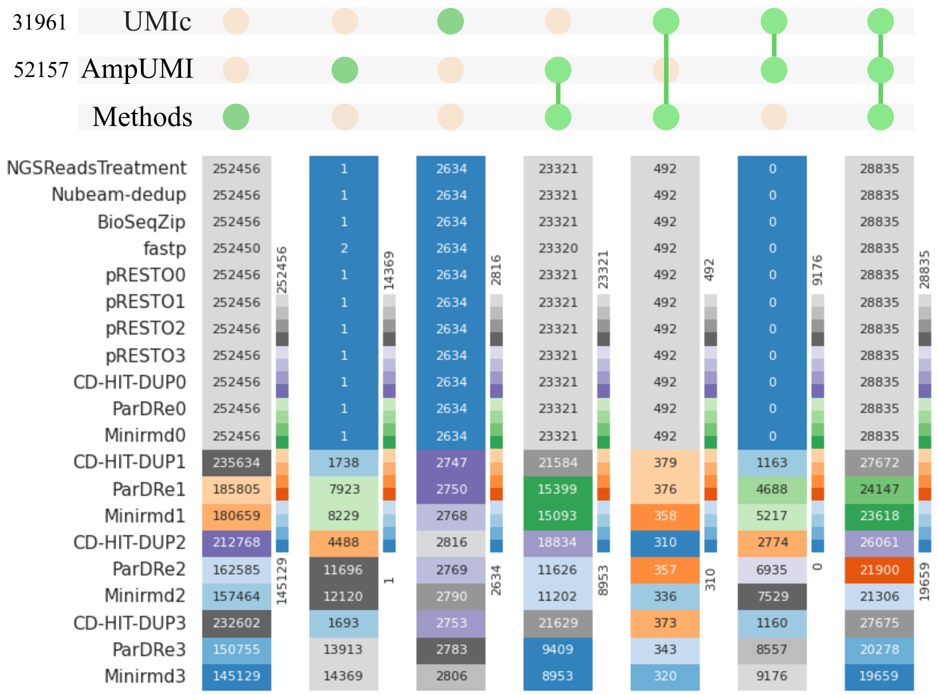

Figure S12: Heatmap for comparing overlapped reads number by each of the computational methods of NGSReadsTreatment, Nubeam-dedup, BioSeqZip, fastp, FastUniq, pRESTO, CD-HIT-DUP, ParDRe and Minirmd with the UMI-based methods of AmpUMI and UMIc on the data set SRR1543966.

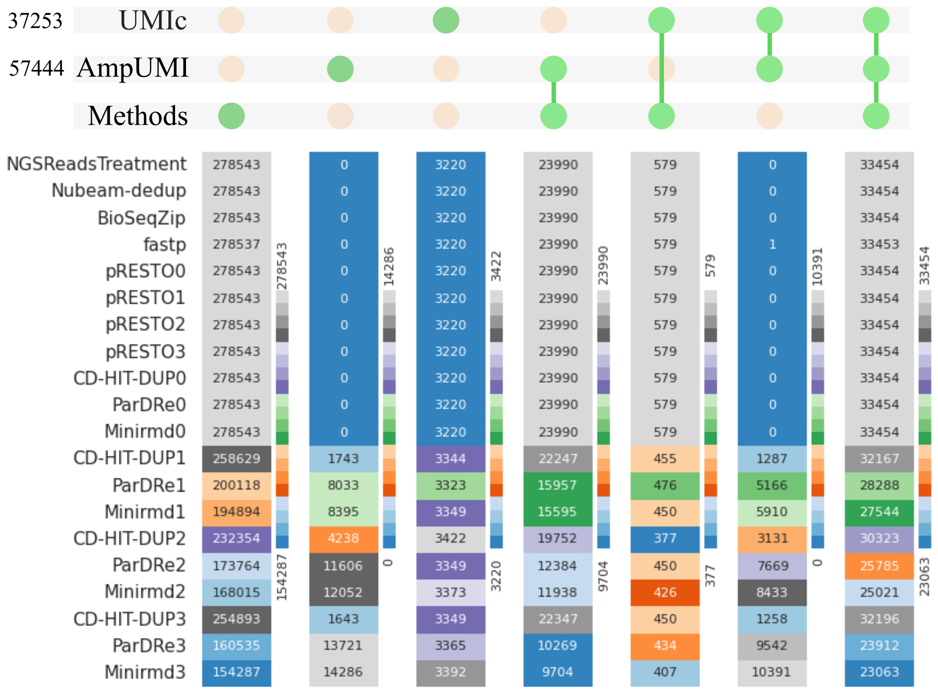

Figure S13: Heatmap for comparing overlapped reads number by each of the computational methods of NGSReadsTreatment, Nubeam-dedup, BioSeqZip, fastp, FastUniq, pRESTO, CD-HIT-DUP, ParDRe and Minirmd with the UMI-based methods of AmpUMI and UMIc on the data set SRR1543967.

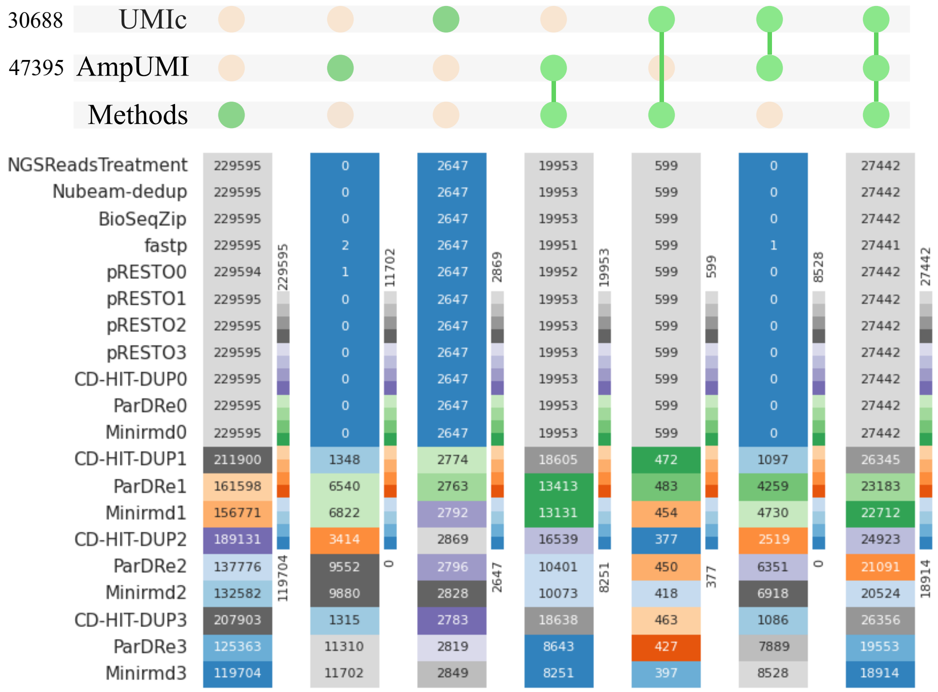

Figure S14: Heatmap for comparing overlapped reads number by each of the computational methods of NGSReadsTreatment, Nubeam-dedup, BioSeqZip, fastp, FastUniq, pRESTO, CD-HIT-DUP, ParDRe and Minirmd with the UMI-based methods of AmpUMI and UMIc on the data set SRR1543968.

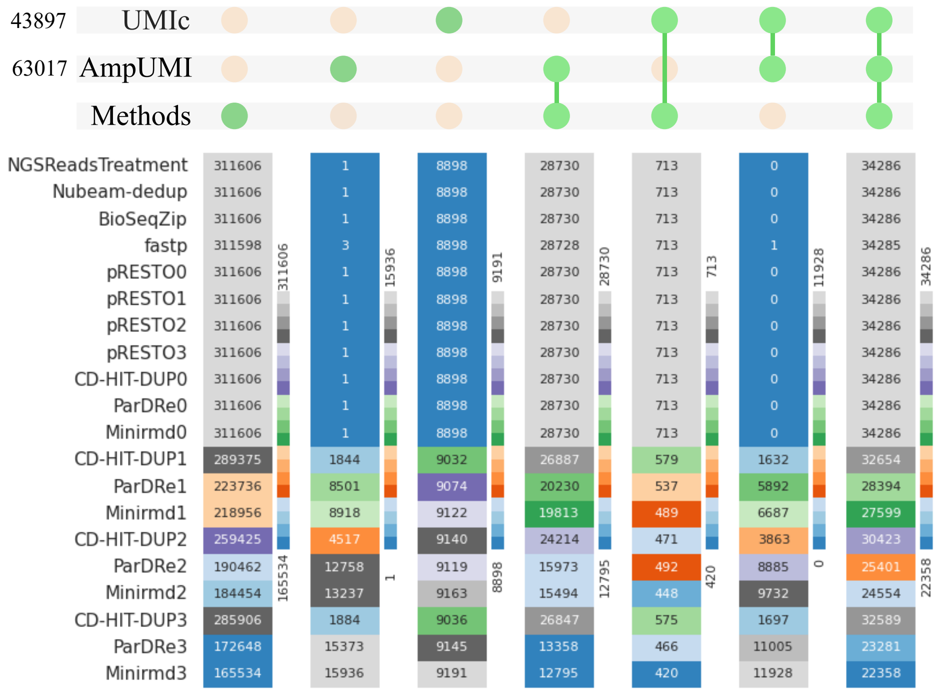

Figure S15: Heatmap for comparing overlapped reads number by each of the computational methods of NGSReadsTreatment, Nubeam-dedup, BioSeqZip, fastp, FastUniq, pRESTO, CD-HIT-DUP, ParDRe and Minirmd with the UMI-based methods of AmpUMI and UMIc on the data set SRR1543969.

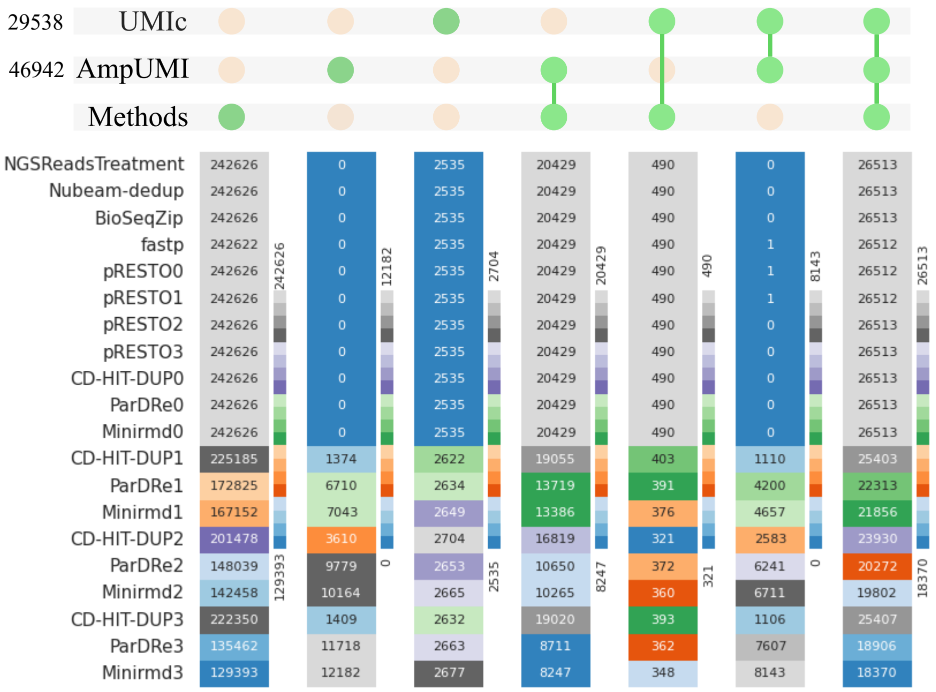

Figure S16: Heatmap for comparing overlapped reads number by each of the computational methods of NGSReadsTreatment, Nubeam-dedup, BioSeqZip, fastp, FastUniq, pRESTO, CD-HIT-DUP, ParDRe and Minirmd with the UMI-based methods of AmpUMI and UMIc on the data set SRR1543970.

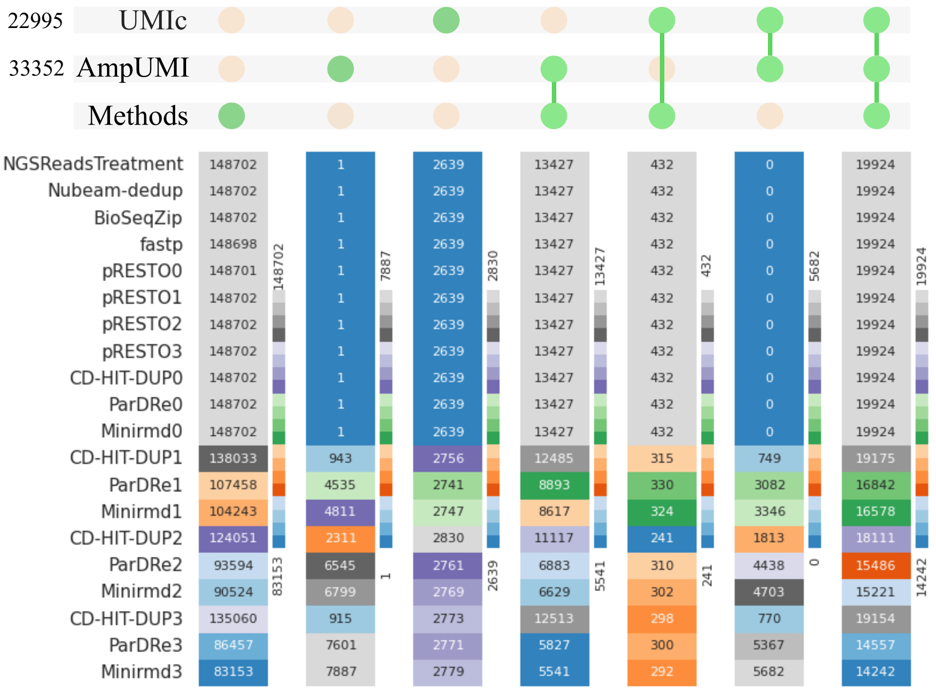

Figure S17: Heatmap for comparing overlapped reads number by each of the computational methods of NGSReadsTreatment, Nubeam-dedup, BioSeqZip, fastp, FastUniq, pRESTO, CD-HIT-DUP, ParDRe and Minirmd with the UMI-based methods of AmpUMI and UMIc on the data set SRR1543971.

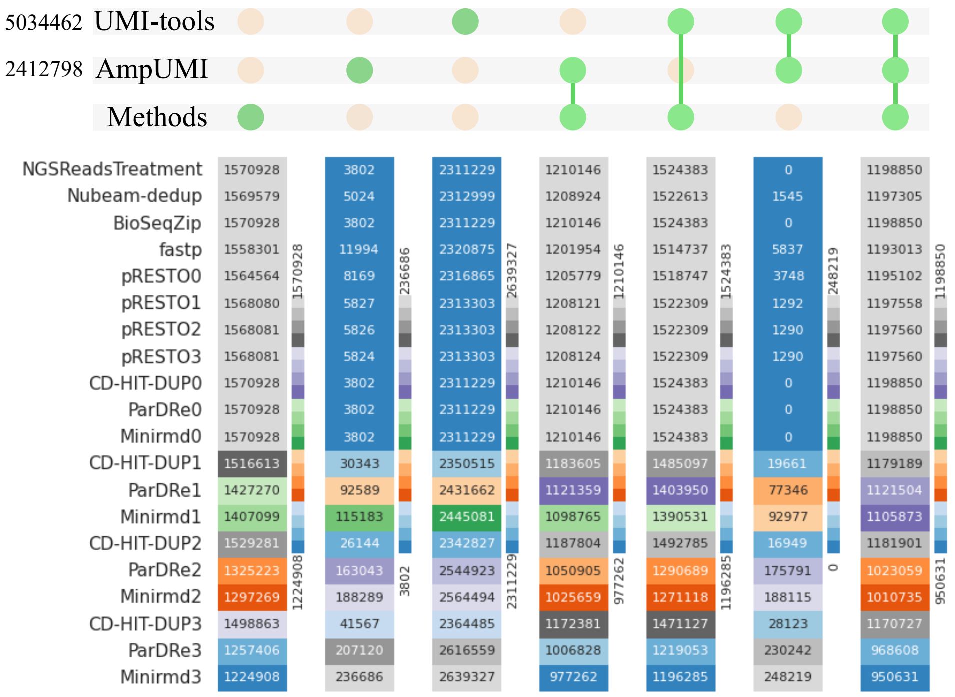

Figure S18: Heatmap for comparing overlapped reads number by each of the computational methods of NGSReadsTreatment, Nubeam-dedup, BioSeqZip, fastp, FastUniq, pRESTO, CD-HIT-DUP, ParDRe and Minirmd with the UMI-based methods of AmpUMI and UMI-tools on the data set SRR28313972.

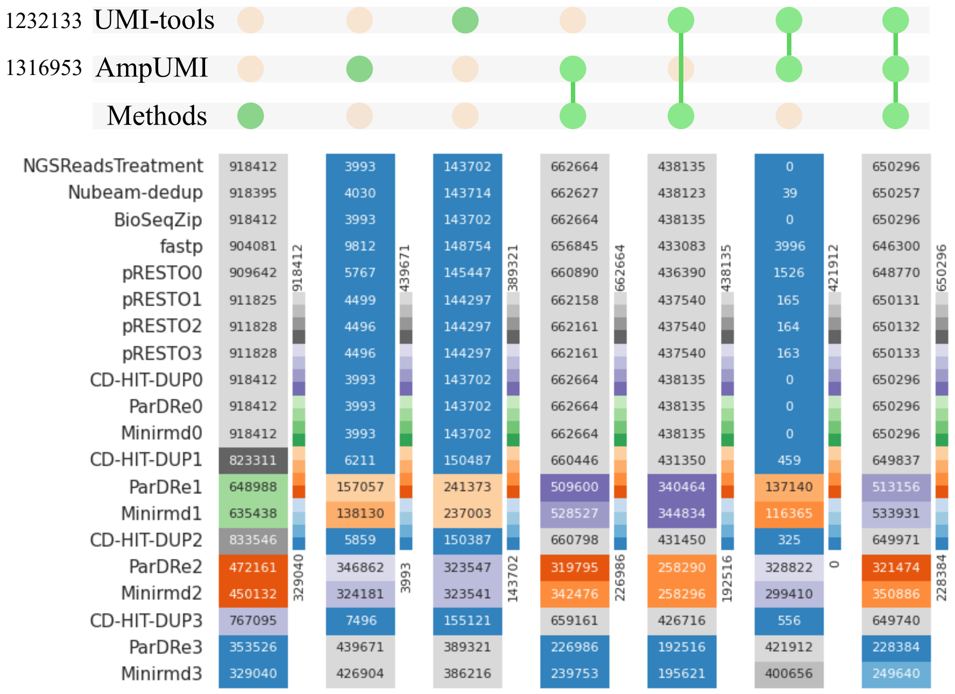

Figure S19: Heatmap for comparing overlapped reads number by each of the computational methods of NGSReadsTreatment, Nubeam-dedup, BioSeqZip, fastp, FastUniq, pRESTO, CD-HIT-DUP, ParDRe and Minirmd with the UMI-based methods of AmpUMI and UMI-tools on the data set SRR28313990.

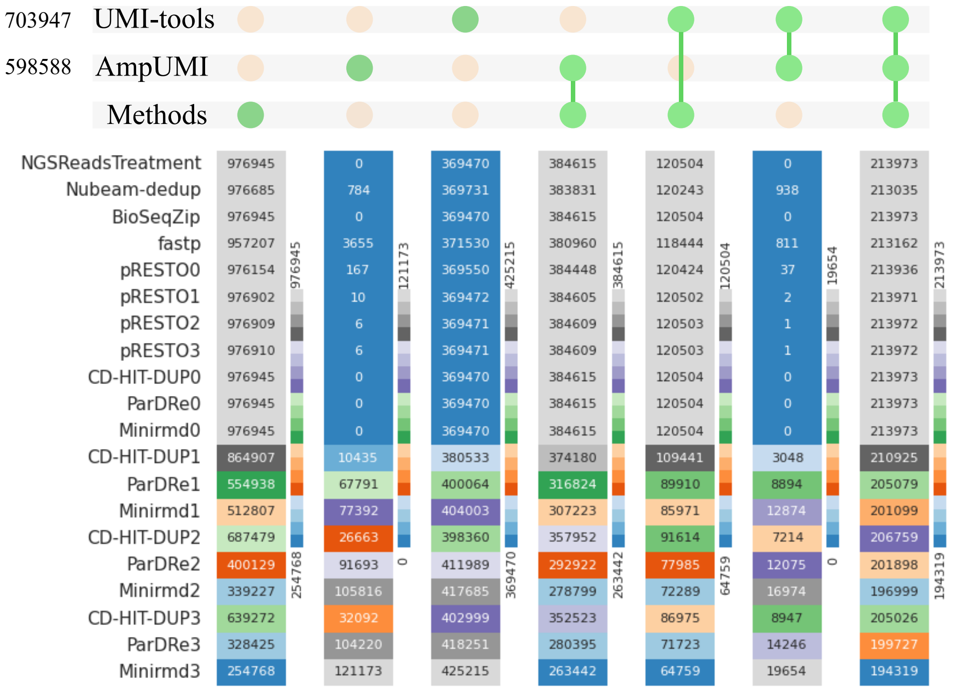

Figure S20: Heatmap for comparing overlapped reads number by each of the computational methods of NGSReadsTreatment, Nubeam-dedup, BioSeqZip, fastp, FastUniq, pRESTO, CD-HIT-DUP, ParDRe and Minirmd with the UMI-based methods of AmpUMI and UMI-tools on the data set SRR28314008.

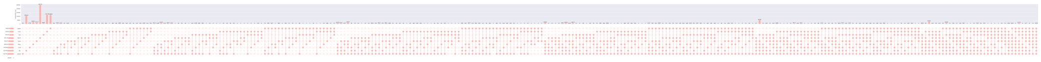

Figure S21: The overlaps and differences comparison between the unique read sets obtained by different error correction algorithms using UpSet plots on data sets SRR1543964. The original high-resolution figure is a long-scale picture presented in the attachment separately in png format.

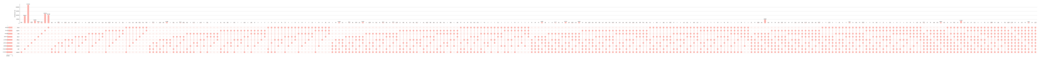

Figure S22: The overlaps and differences comparison between the unique read sets obtained by different error correction algorithms using UpSet plots on data sets SRR1543965. The original high-resolution figure is a long-scale picture presented in the attachment separately in png format.

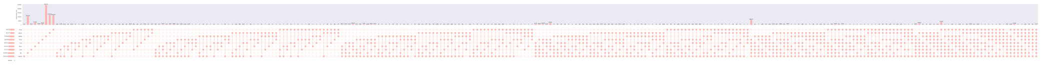

Figure S23: The overlaps and differences comparison between the unique read sets obtained by different error correction algorithms using UpSet plots on data sets SRR1543966. The original high-resolution figure is a long-scale picture presented in the attachment separately in png format.

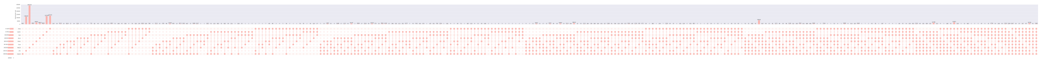

Figure S24: The overlaps and differences comparison between the unique read sets obtained by different error correction algorithms using UpSet plots on data sets SRR1543967. The original high-resolution figure is a long-scale picture presented in the attachment separately in png format.

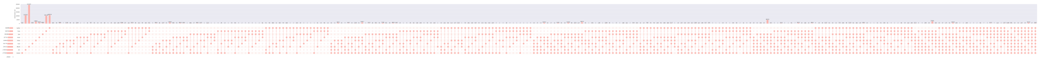

Figure S25: The overlaps and differences comparison between the unique read sets obtained by different error correction algorithms using UpSet plots on data sets SRR1543968. The original high-resolution figure is a long-scale picture presented in the attachment separately in png format.

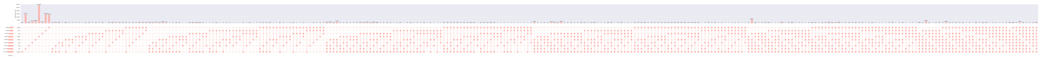

Figure S26: The overlaps and differences comparison between the unique read sets obtained by different error correction algorithms using UpSet plots on data sets SRR1543969. The original high-resolution figure is a long-scale picture presented in the attachment separately in png format.

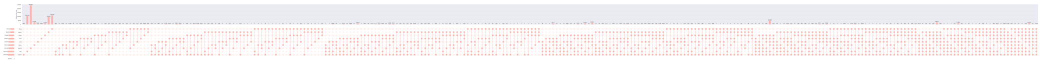

Figure S27: The overlaps and differences comparison between the unique read sets obtained by different error correction algorithms using UpSet plots on data sets SRR1543970. The original high-resolution figure is a long-scale picture presented in the attachment separately in png format.

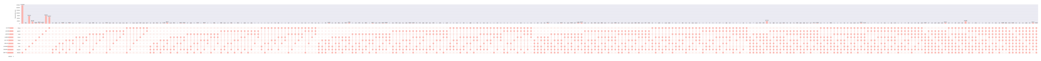

Figure S28: The overlaps and differences comparison between the unique read sets obtained by different error correction algorithms using UpSet plots on data sets SRR1543971. The original figure is a long-scale picture presented in the attachment separately in png format.

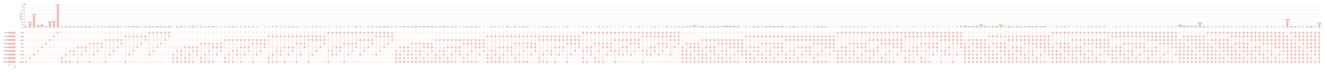

Figure S29: The overlaps and differences comparison between the unique read sets obtained by different error correction algorithms using UpSet plots on data sets SRR28313972. The original figure is a long-scale picture presented in the attachment separately in png format.

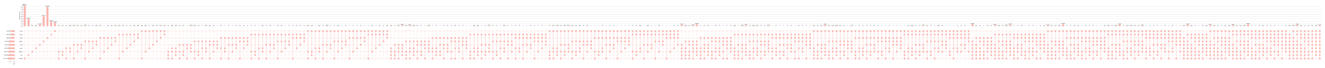

Figure S30: The overlaps and differences comparison between the unique read sets obtained by different error correction algorithms using UpSet plots on data sets SRR28313990. The original figure is a long-scale picture presented in the attachment separately in png format.

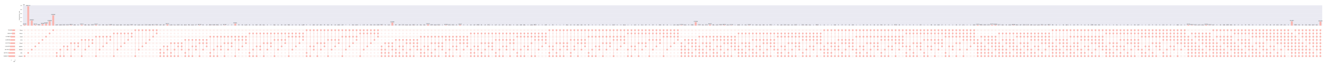

Figure S31: The overlaps and differences comparison between the unique read sets obtained by different error correction algorithms using UpSet plots on data sets SRR28314008. The original figure is a long-scale picture presented in the attachment separately in png format.

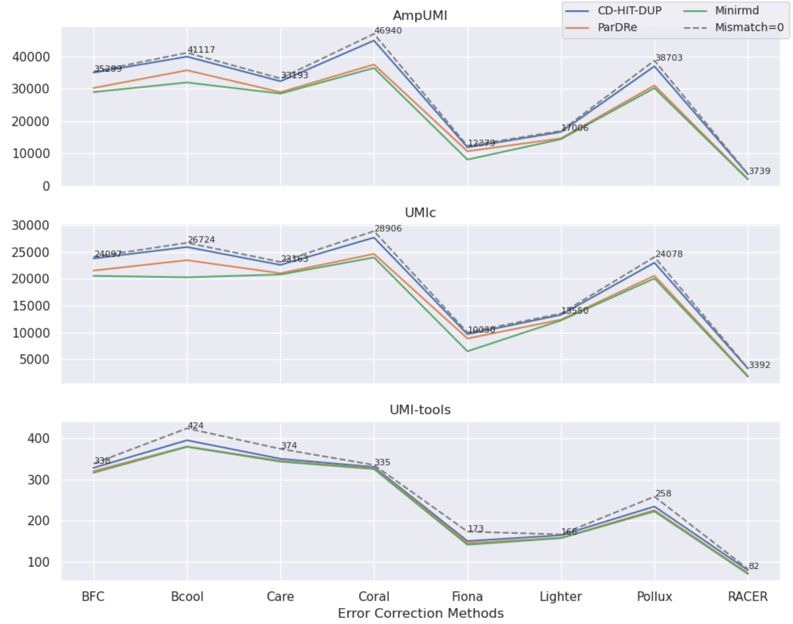

Figure S32: Line charts comparing the overlapped read numbers in the deduplicated read set by the PCR-deduplication methods of CD-HIT-DUP, ParDRe, and Minirmd on the error-corrected dataset SRR1543964, with each UMI-based PCR-deduplication methods of UMI-tools, AmpUMI, and UMIc on dataset SRR1543964. Error correction was performed using error-correction methods of BFC, Bcool, Care, Coral, Fiona, Lighter, Pollux, and RACER, respectively. CD-HIT-DUP, ParDRe, and Minirmd employed a mismatched number set to 1. The dashed line labelled 'Mismatch=0' represents results obtained by CD-HIT-DUP with a mismatch setting of 0.

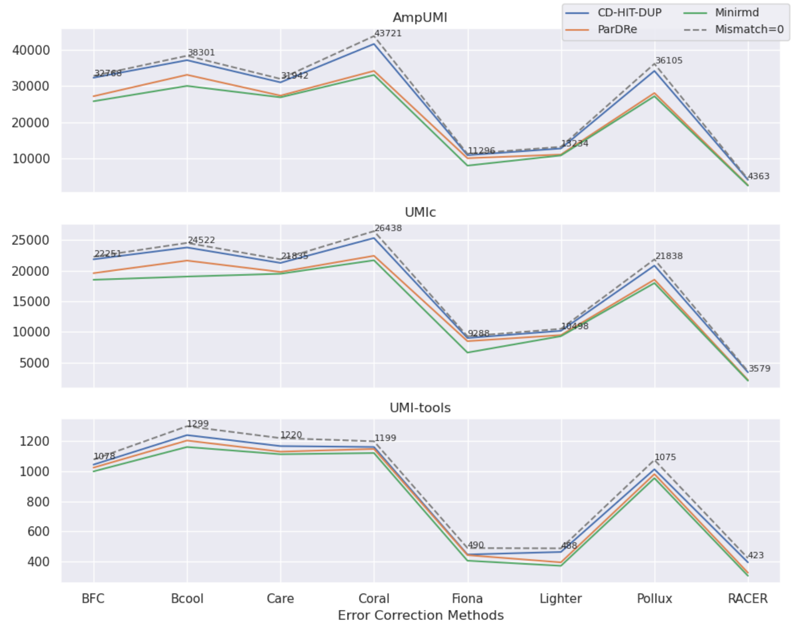

Figure S33: Line charts comparing the overlapped read numbers in the deduplicated read set by the PCR-deduplication methods of CD-HIT-DUP, ParDRe, and Minirmd on the error-corrected dataset SRR1543965, with each UMI-based PCR-deduplication methods of UMI-tools, AmpUMI, and UMIc on dataset SRR1543965. Error correction was performed using error-correction methods of BFC, Bcool, Care, Coral, Fiona, Lighter, Pollux, and RACER, respectively. CD-HIT-DUP, ParDRe, and Minirmd employed a mismatched number set to 1. The dashed line labelled 'Mismatch=0' represents results obtained by CD-HIT-DUP with a mismatch setting of 0.

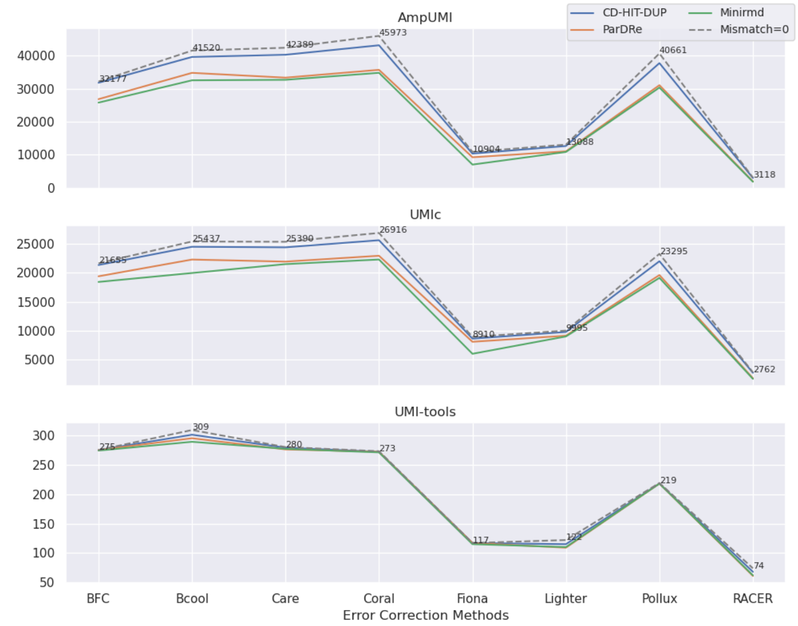

Figure S34: Line charts comparing the overlapped read numbers in the deduplicated read set by the PCR-deduplication methods of CD-HIT-DUP, ParDRe, and Minirmd on the error-corrected dataset SRR1543966, with each UMI-based PCR-deduplication methods of UMI-tools, AmpUMI, and UMIc on dataset SRR1543966. Error correction was performed using error-correction methods of BFC, Bcool, Care, Coral, Fiona, Lighter, Pollux, and RACER, respectively. CD-HIT-DUP, ParDRe, and Minirmd employed a mismatched number set to 1. The dashed line labelled 'Mismatch=0' represents results obtained by CD-HIT-DUP with a mismatch setting of 0.

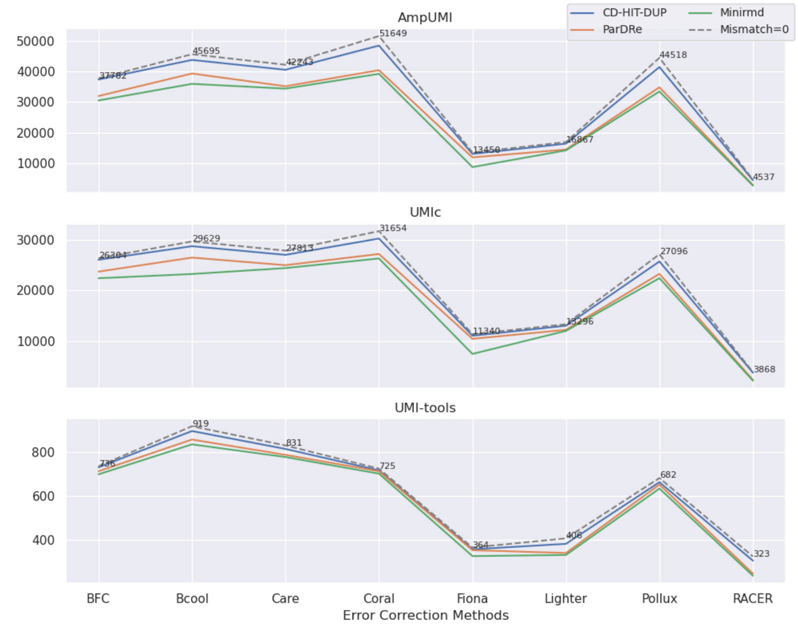

Figure S35: Line charts comparing the overlapped read numbers in the deduplicated read set by the PCR-deduplication methods of CD-HIT-DUP, ParDRe, and Minirmd on the error-corrected dataset SRR1543967, with each UMI-based PCR-deduplication methods of UMI-tools, AmpUMI, and UMIc on dataset SRR1543967. Error correction was performed using error-correction methods of BFC, Bcool, Care, Coral, Fiona, Lighter, Pollux, and RACER, respectively. CD-HIT-DUP, ParDRe, and Minirmd employed a mismatched number set to 1. The dashed line labelled 'Mismatch=0' represents results obtained by CD-HIT-DUP with a mismatch setting of 0.

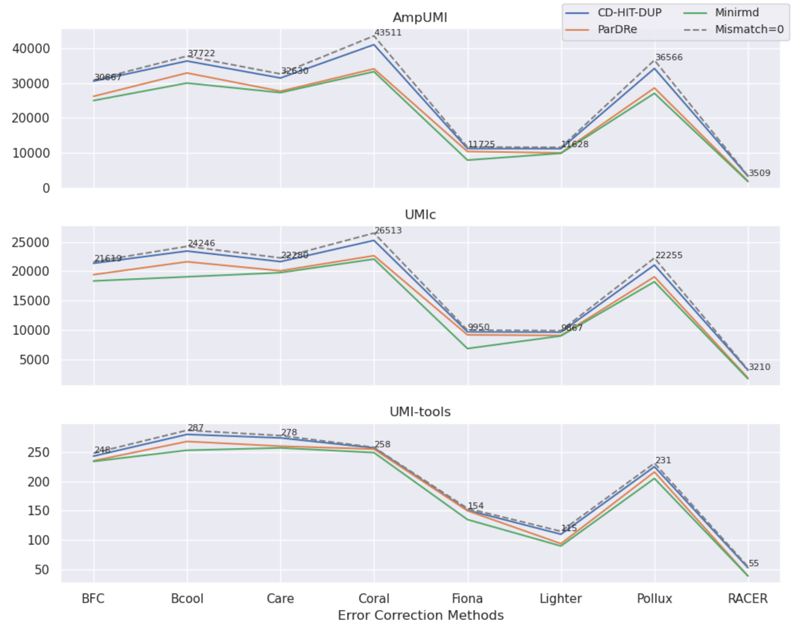

Figure S36: Line charts comparing the overlapped read numbers in the deduplicated read set by the PCR-deduplication methods of CD-HIT-DUP, ParDRe, and Minirmd on the error-corrected dataset SRR1543968, with each UMI-based PCR-deduplication methods of UMI-tools, AmpUMI, and UMIc on dataset SRR1543968. Error correction was performed using error-correction methods of BFC, Bcool, Care, Coral, Fiona, Lighter, Pollux, and RACER, respectively. CD-HIT-DUP, ParDRe, and Minirmd employed a mismatched number set to 1. The dashed line labelled 'Mismatch=0' represents results obtained by CD-HIT-DUP with a mismatch setting of 0.

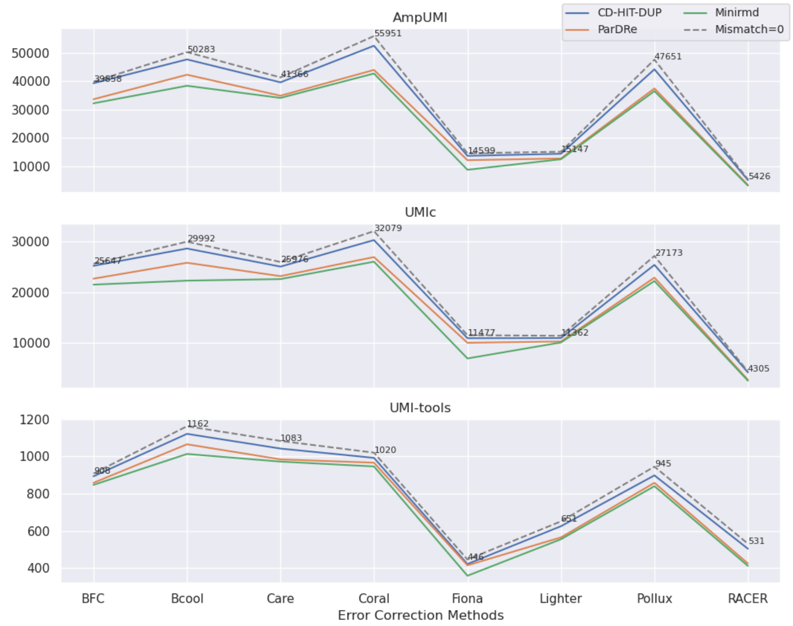

Figure S37: Line charts comparing the overlapped read numbers in the deduplicated read set by the PCR-deduplication methods of CD-HIT-DUP, ParDRe, and Minirmd on the error-corrected dataset SRR1543969, with each UMI-based PCR-deduplication methods of UMI-tools, AmpUMI, and UMIc on dataset SRR1543969. Error correction was performed using error-correction methods of BFC, Bcool, Care, Coral, Fiona, Lighter, Pollux, and RACER, respectively. CD-HIT-DUP, ParDRe, and Minirmd employed a mismatched number set to 1. The dashed line labelled 'Mismatch=0' represents results obtained by CD-HIT-DUP with a mismatch setting of 0.

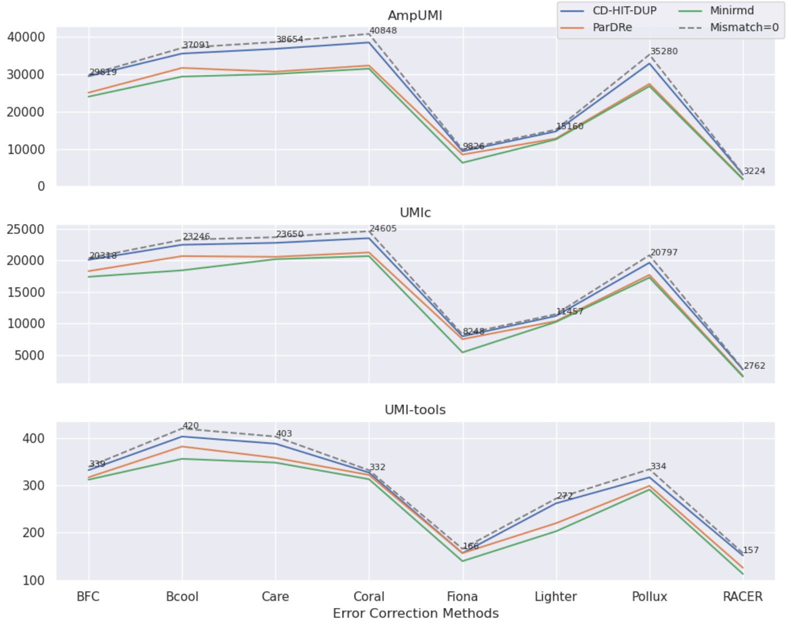

Figure S38: Line charts comparing the overlapped read numbers in the deduplicated read set by the PCR-deduplication methods of CD-HIT-DUP, ParDRe, and Minirmd on the error-corrected dataset SRR1543970, with each UMI-based PCR-deduplication methods of UMI-tools, AmpUMI, and UMIc on dataset SRR1543970. Error correction was performed using error-correction methods of BFC, Bcool, Care, Coral, Fiona, Lighter, Pollux, and RACER, respectively. CD-HIT-DUP, ParDRe, and Minirmd employed a mismatched number set to 1. The dashed line labelled ‘Mismatch=0’ represents results obtained by CD-HIT-DUP with a mismatch setting of 0.

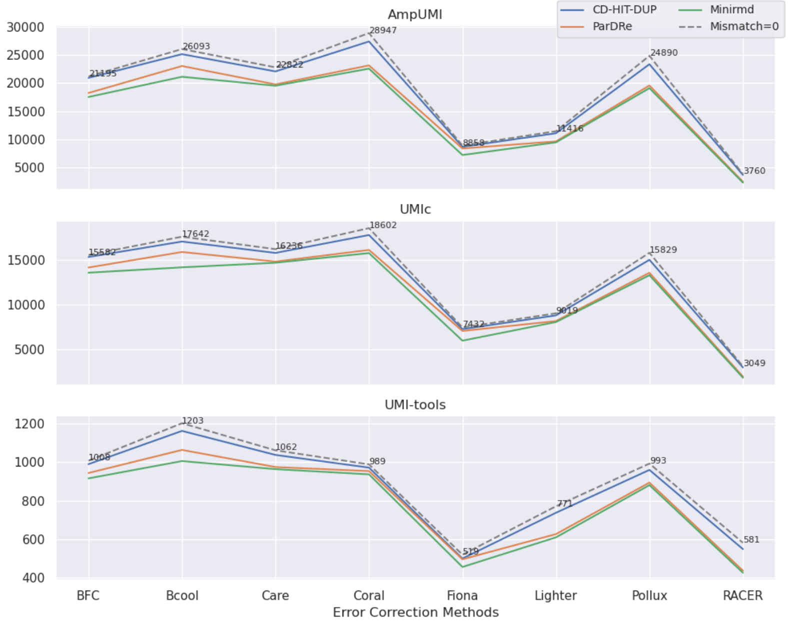

Figure S39: Line charts comparing the overlapped read numbers in the deduplicated read set by the PCR-deduplication methods of CD-HIT-DUP, ParDRe, and Minirmd on the error-corrected dataset SRR1543971, with each UMI-based PCR-deduplication methods of UMI-tools, AmpUMI, and UMIc on dataset SRR1543971. Error correction was performed using error-correction methods of BFC, Bcool, Care, Coral, Fiona, Lighter, Pollux, and RACER, respectively. CD-HIT-DUP, ParDRe, and Minirmd employed a mismatched number set to 1. The dashed line labelled ‘Mismatch=0’ represents results obtained by CD-HIT-DUP with a mismatch setting of 0.

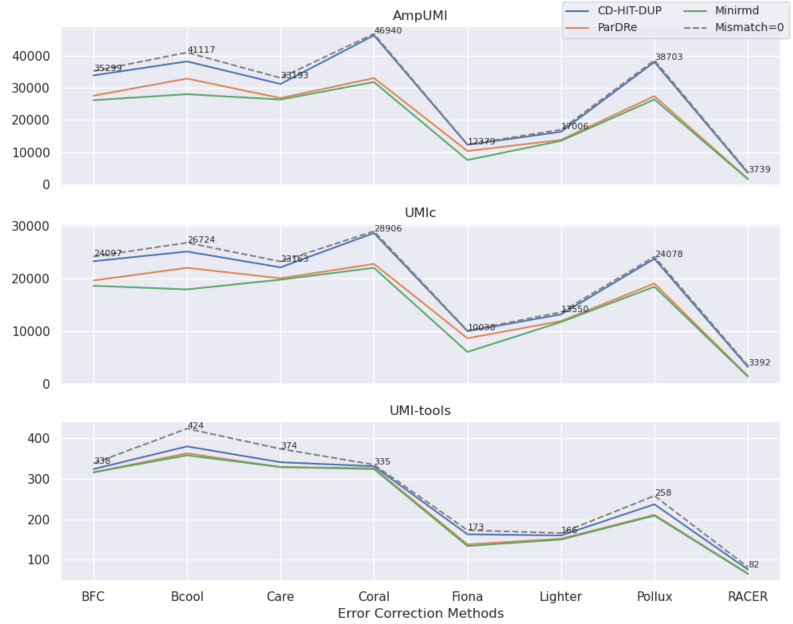

Figure S40: Line charts comparing the overlapped read numbers in the deduplicated read set by the PCR-deduplication methods of CD-HIT-DUP, ParDRe, and Minirmd on the error-corrected dataset SRR1543964, with each UMI-based PCR-deduplication methods of UMI-tools, AmpUMI, and UMIc on dataset SRR1543964. Error correction was performed using error-correction methods of BFC, Bcool, Care, Coral, Fiona, Lighter, Pollux, and RACER, respectively. CD-HIT-DUP, ParDRe, and Minirmd employed a mismatched number set to 2. The dashed line labelled 'Mismatch=0' represents results obtained by CD-HIT-DUP with a mismatch setting of 0.

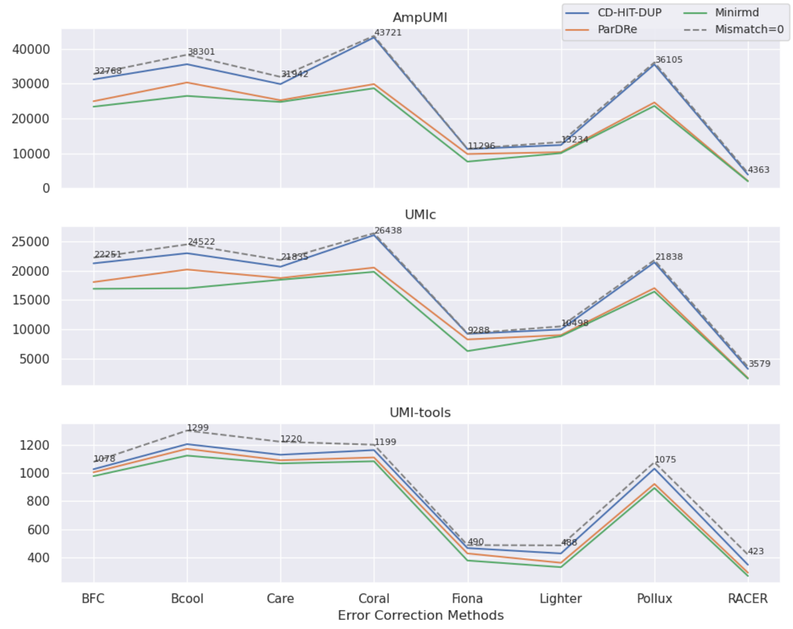

Figure S41: Line charts comparing the overlapped read numbers in the deduplicated read set by the PCR-deduplication methods of CD-HIT-DUP, ParDRe, and Minirmd on the error-corrected dataset SRR1543965, with each UMI-based PCR-deduplication methods of UMI-tools, AmpUMI, and UMIc on dataset SRR1543965. Error correction was performed using error-correction methods of BFC, Bcool, Care, Coral, Fiona, Lighter, Pollux, and RACER, respectively. CD-HIT-DUP, ParDRe, and Minirmd employed a mismatched number set to 2. The dashed line labelled 'Mismatch=0' represents results obtained by CD-HIT-DUP with a mismatch setting of 0.

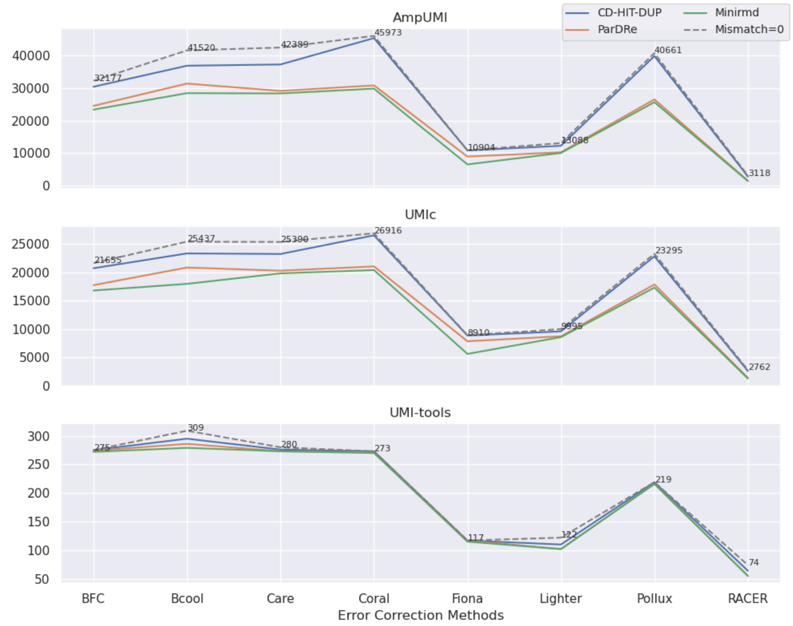

Figure S42: Line charts comparing the overlapped read numbers in the deduplicated read set by the PCR-deduplication methods of CD-HIT-DUP, ParDRe, and Minirmd on the error-corrected dataset SRR1543966, with each UMI-based PCR-deduplication methods of UMI-tools, AmpUMI, and UMIc on dataset SRR1543966. Error correction was performed using error-correction methods of BFC, Bcool, Care, Coral, Fiona, Lighter, Pollux, and RACER, respectively. CD-HIT-DUP, ParDRe, and Minirmd employed a mismatched number set to 2. The dashed line labelled 'Mismatch=0' represents results obtained by CD-HIT-DUP with a mismatch setting of 0.

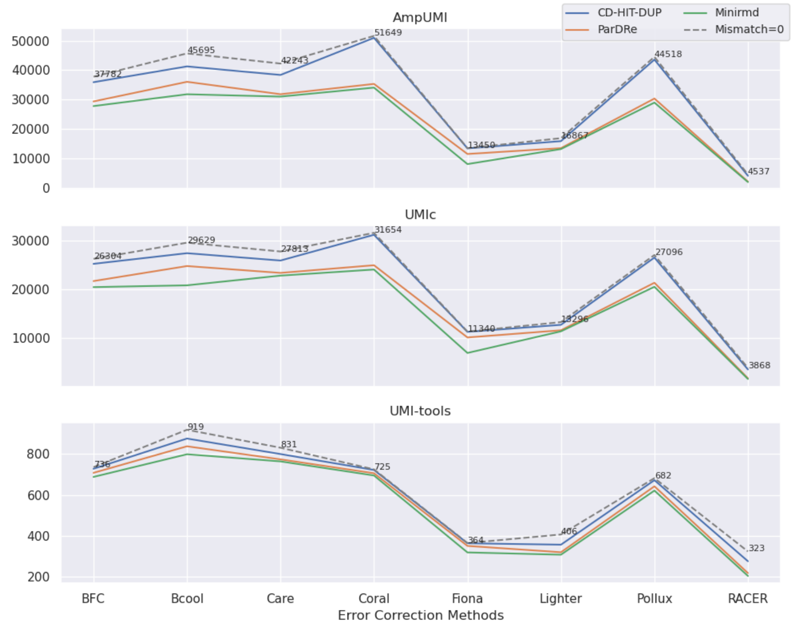

Figure S43: Line charts comparing the overlapped read numbers in the deduplicated read set by the PCR-deduplication methods of CD-HIT-DUP, ParDRe, and Minirmd on the error-corrected dataset SRR1543967, with each UMI-based PCR-deduplication methods of UMI-tools, AmpUMI, and UMIc on dataset SRR1543967. Error correction was performed using error-correction methods of BFC, Bcool, Care, Coral, Fiona, Lighter, Pollux, and RACER, respectively. CD-HIT-DUP, ParDRe, and Minirmd employed a mismatched number set to 2. The dashed line labelled 'Mismatch=0' represents results obtained by CD-HIT-DUP with a mismatch setting of 0.

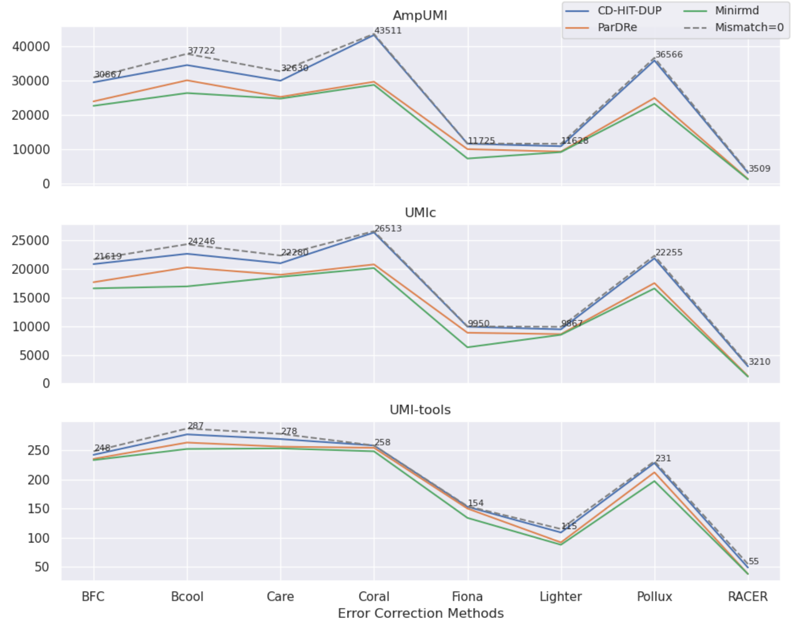

Figure S44: Line charts comparing the overlapped read numbers in the deduplicated read set by the PCR-deduplication methods of CD-HIT-DUP, ParDRe, and Minirmd on the error-corrected dataset SRR1543968, with each UMI-based PCR-deduplication methods of UMI-tools, AmpUMI, and UMIc on dataset SRR1543968. Error correction was performed using error-correction methods of BFC, Bcool, Care, Coral, Fiona, Lighter, Pollux, and RACER, respectively. CD-HIT-DUP, ParDRe, and Minirmd employed a mismatched number set to 2. The dashed line labelled ‘Mismatch=0’ represents results obtained by CD-HIT-DUP with a mismatch setting of 0.

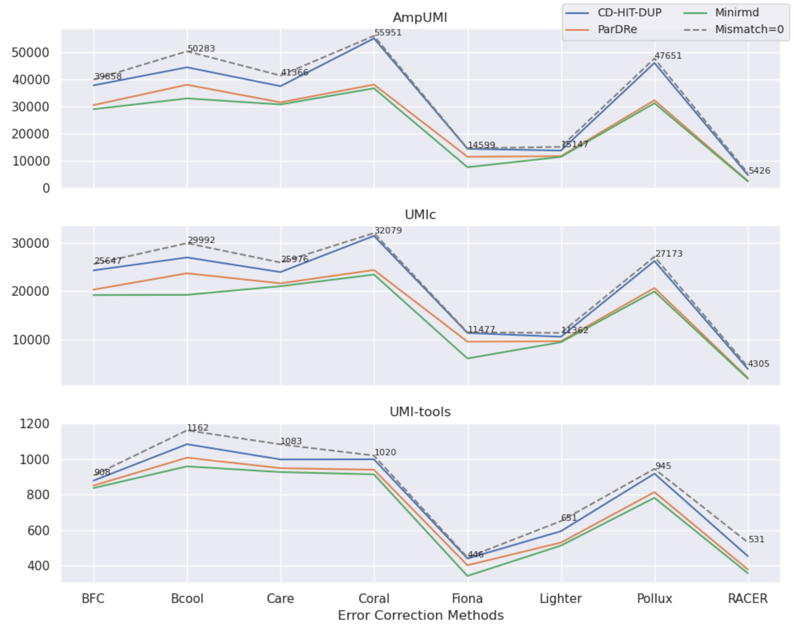

Figure S45: Line charts comparing the overlapped read numbers in the deduplicated read set by the PCR-deduplication methods of CD-HIT-DUP, ParDRe, and Minirmd on the error-corrected dataset SRR1543969, with each UMI-based PCR-deduplication methods of UMI-tools, AmpUMI, and UMIc on dataset SRR1543969. Error correction was performed using error-correction methods of BFC, Bcool, Care, Coral, Fiona, Lighter, Pollux, and RACER, respectively. CD-HIT-DUP, ParDRe, and Minirmd employed a mismatched number set to 2. The dashed line labelled ‘Mismatch=0’ represents results obtained by CD-HIT-DUP with a mismatch setting of 0.

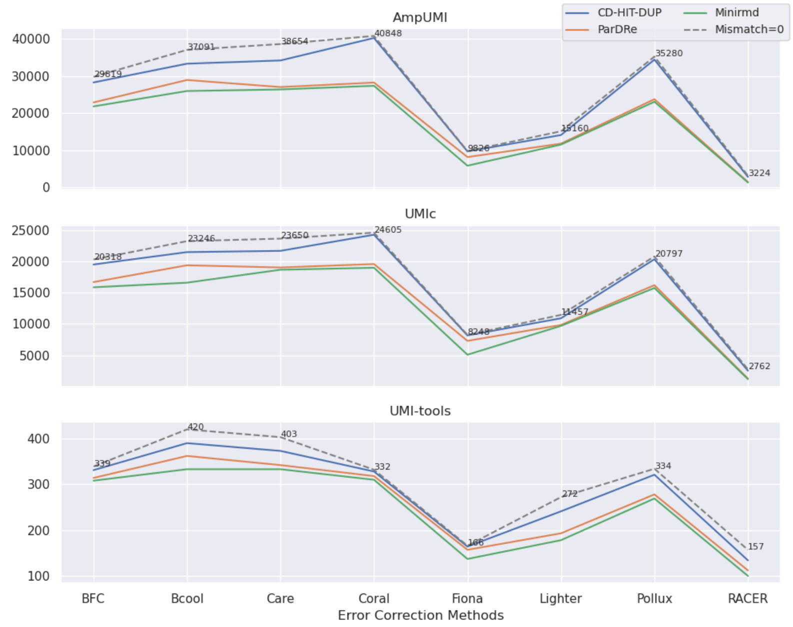

Figure S46: Line charts comparing the overlapped read numbers in the deduplicated read set by the PCR-deduplication methods of CD-HIT-DUP, ParDRe, and Minirmd on the error-corrected dataset SRR1543970, with each UMI-based PCR-deduplication methods of UMI-tools, AmpUMI, and UMIc on dataset SRR1543970. Error correction was performed using error-correction methods of BFC, Bcool, Care, Coral, Fiona, Lighter, Pollux, and RACER, respectively. CD-HIT-DUP, ParDRe, and Minirmd employed a mismatched number set to 2. The dashed line labelled 'Mismatch=0' represents results obtained by CD-HIT-DUP with a mismatch setting of 0.

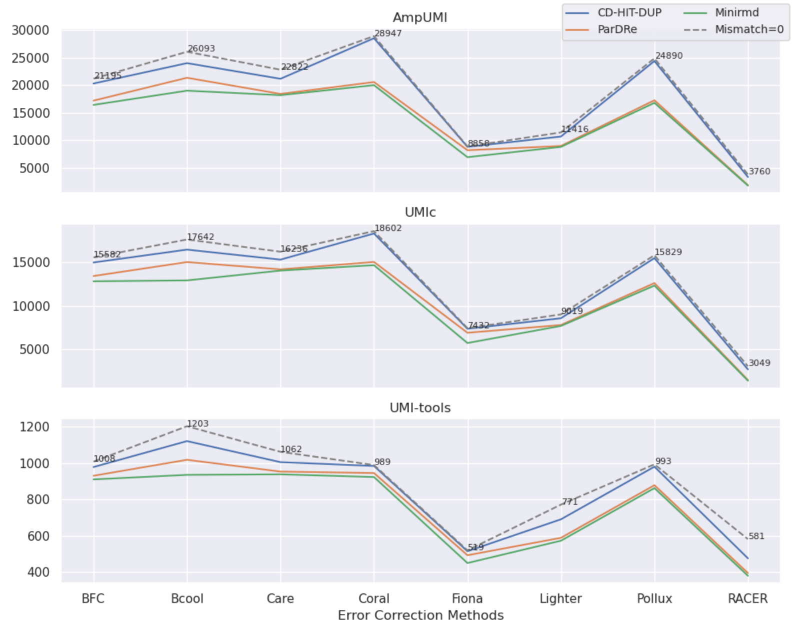

Figure S47: Line charts comparing the overlapped read numbers in the deduplicated read set by the PCR-deduplication methods of CD-HIT-DUP, ParDRe, and Minirmd on the error-corrected dataset SRR1543971, with each UMI-based PCR-deduplication methods of UMI-tools, AmpUMI, and UMIc on dataset SRR1543971. Error correction was performed using error-correction methods of BFC, Bcool, Care, Coral, Fiona, Lighter, Pollux, and RACER, respectively. CD-HIT-DUP, ParDRe, and Minirmd employed a mismatched number set to 2. The dashed line labelled 'Mismatch=0' represents results obtained by CD-HIT-DUP with a mismatch setting of 0.

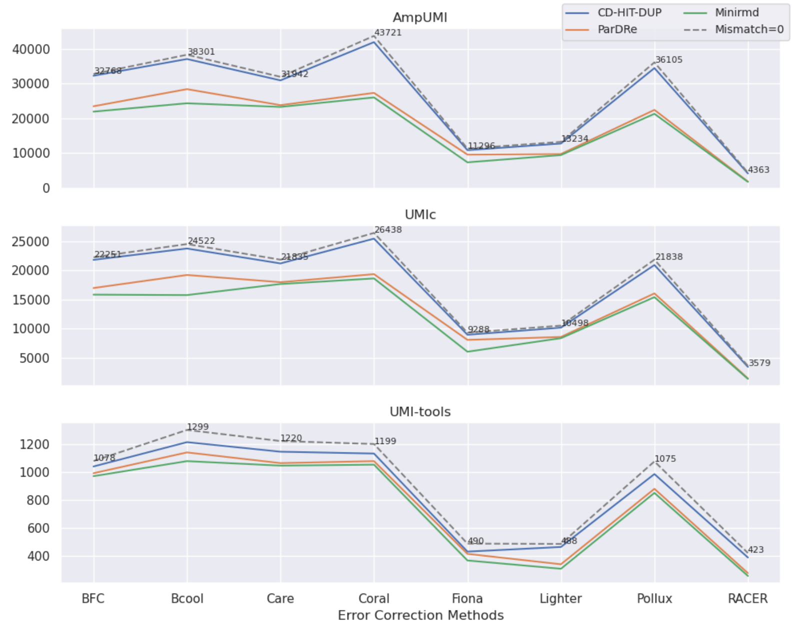

Figure S48: Line charts comparing the overlapped read numbers in the deduplicated read set by the PCR-deduplication methods of CD-HIT-DUP, ParDRe, and Minirmd on the error-corrected dataset SRR1543965, with each UMI-based PCR-deduplication methods of UMI-tools, AmpUMI, and UMIc on dataset SRR1543965. Error correction was performed using error-correction methods of BFC, Bcool, Care, Coral, Fiona, Lighter, Pollux, and RACER, respectively. CD-HIT-DUP, ParDRe, and Minirmd employed a mismatched number set to 3. The dashed line labelled ‘Mismatch=0’ represents results obtained by CD-HIT-DUP with a mismatch setting of 0.

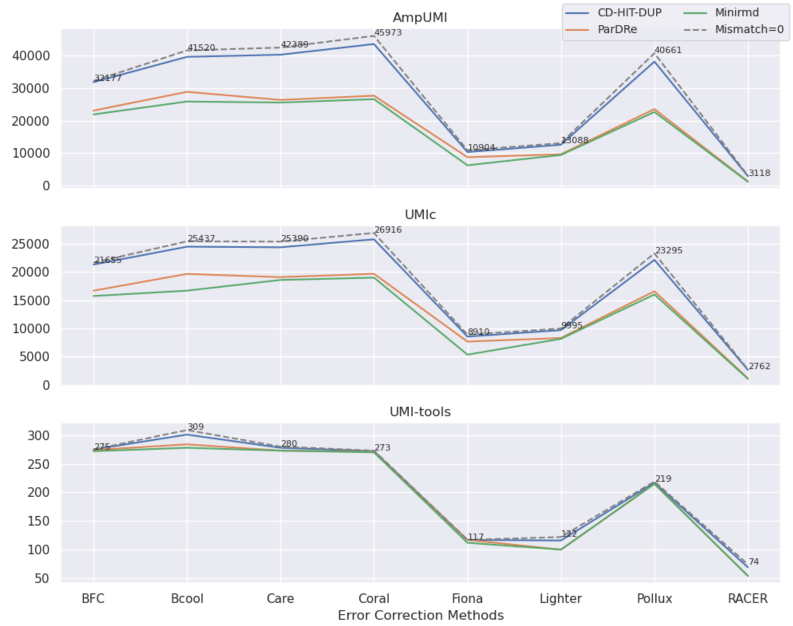

Figure S49: Line charts comparing the overlapped read numbers in the deduplicated read set by the PCR-deduplication methods of CD-HIT-DUP, ParDRe, and Minirmd on the error-corrected dataset SRR1543966, with each UMI-based PCR-deduplication methods of UMI-tools, AmpUMI, and UMIc on dataset SRR1543966. Error correction was performed using error-correction methods of BFC, Bcool, Care, Coral, Fiona, Lighter, Pollux, and RACER, respectively. CD-HIT-DUP, ParDRe, and Minirmd employed a mismatched number set to 3. The dashed line labelled ‘Mismatch=0’ represents results obtained by CD-HIT-DUP with a mismatch setting of 0.

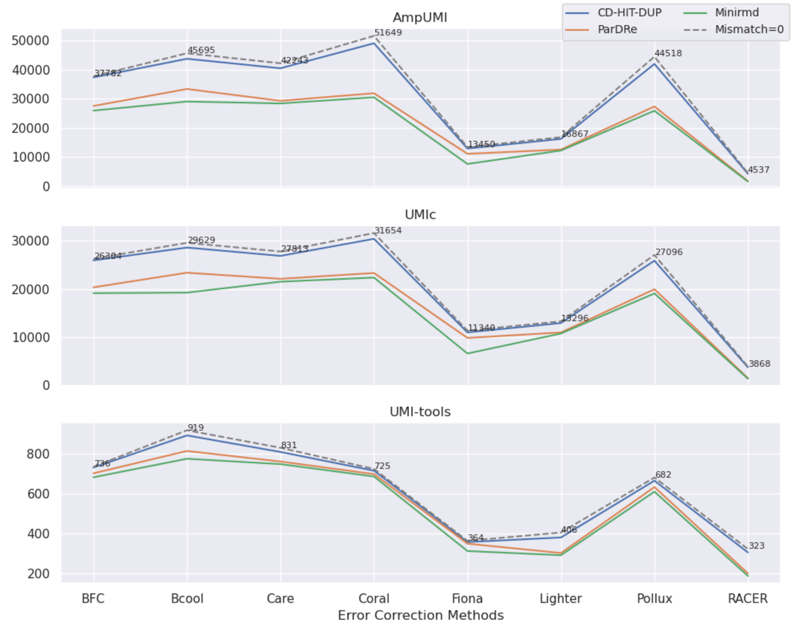

Figure S50: Line charts comparing the overlapped read numbers in the deduplicated read set by the PCR-deduplication methods of CD-HIT-DUP, ParDRe, and Minirmd on the error-corrected dataset SRR1543967, with each UMI-based PCR-deduplication methods of UMI-tools, AmpUMI, and UMIc on dataset SRR1543967. Error correction was performed using error-correction methods of BFC, Bcool, Care, Coral, Fiona, Lighter, Pollux, and RACER, respectively. CD-HIT-DUP, ParDRe, and Minirmd employed a mismatched number set to 3. The dashed line labelled ‘Mismatch=0’ represents results obtained by CD-HIT-DUP with a mismatch setting of 0.

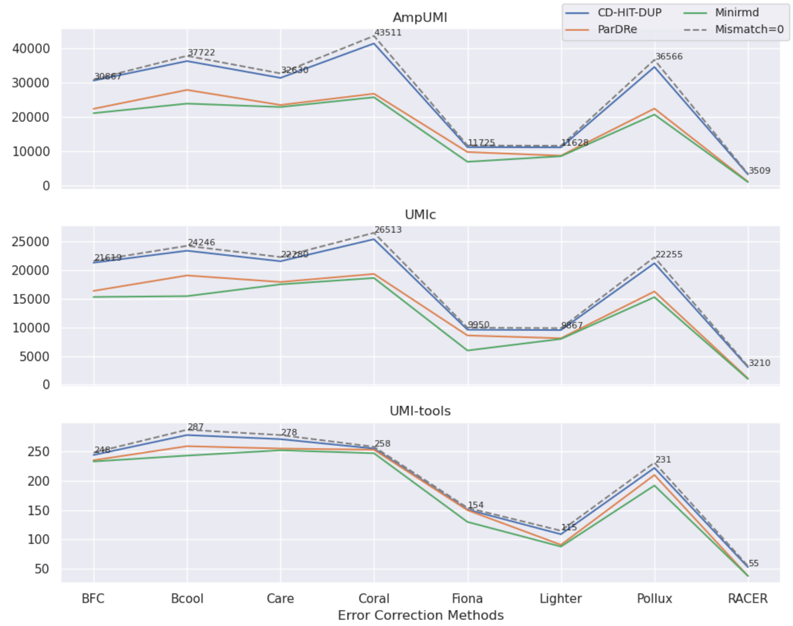

Figure S51: Line charts comparing the overlapped read numbers in the deduplicated read set by the PCR-deduplication methods of CD-HIT-DUP, ParDRe, and Minirmd on the error-corrected dataset SRR1543968, with each UMI-based PCR-deduplication methods of UMI-tools, AmpUMI, and UMIc on dataset SRR1543968. Error correction was performed using error-correction methods of BFC, Bcool, Care, Coral, Fiona, Lighter, Pollux, and RACER, respectively. CD-HIT-DUP, ParDRe, and Minirmd employed a mismatched number set to 3. The dashed line labelled ‘Mismatch=0’ represents results obtained by CD-HIT-DUP with a mismatch setting of 0.

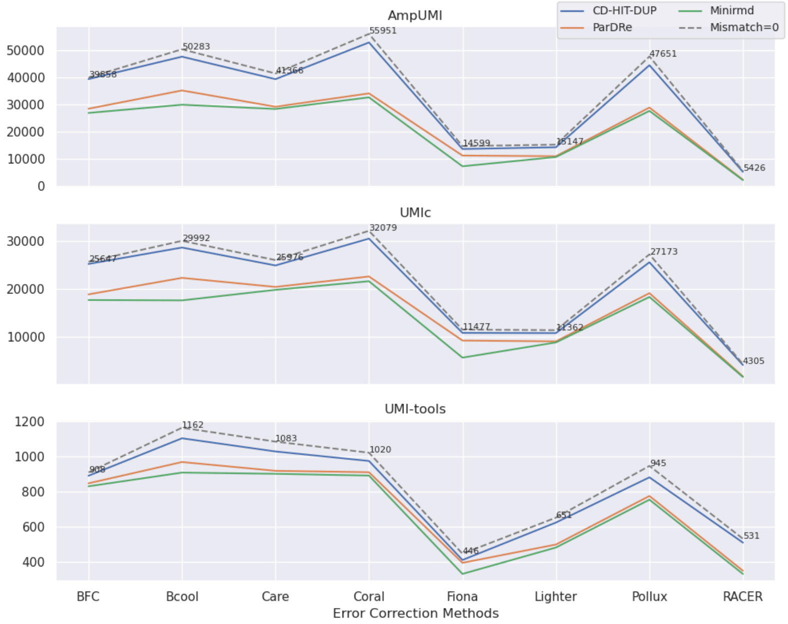

Figure S52: Line charts comparing the overlapped read numbers in the deduplicated read set by the PCR-deduplication methods of CD-HIT-DUP, ParDRe, and Minirmd on the error-corrected dataset SRR1543969, with each UMI-based PCR-deduplication methods of UMI-tools, AmpUMI, and UMIc on dataset SRR1543969. Error correction was performed using error-correction methods of BFC, Bcool, Care, Coral, Fiona, Lighter, Pollux, and RACER, respectively. CD-HIT-DUP, ParDRe, and Minirmd employed a mismatched number set to 3. The dashed line labelled 'Mismatch=0' represents results obtained by CD-HIT-DUP with a mismatch setting of 0.

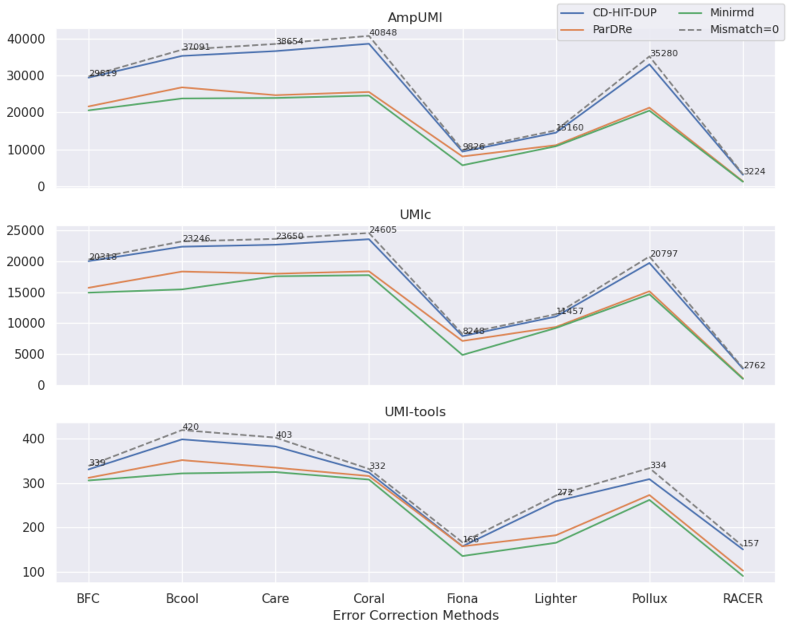

Figure S53: Line charts comparing the overlapped read numbers in the deduplicated read set by the PCR-deduplication methods of CD-HIT-DUP, ParDRe, and Minirmd on the error-corrected dataset SRR1543970, with each UMI-based PCR-deduplication methods of UMI-tools, AmpUMI, and UMIc on dataset SRR1543970. Error correction was performed using error-correction methods of BFC, Bcool, Care, Coral, Fiona, Lighter, Pollux, and RACER, respectively. CD-HIT-DUP, ParDRe, and Minirmd employed a mismatched number set to 3. The dashed line labelled 'Mismatch=0' represents results obtained by CD-HIT-DUP with a mismatch setting of 0.

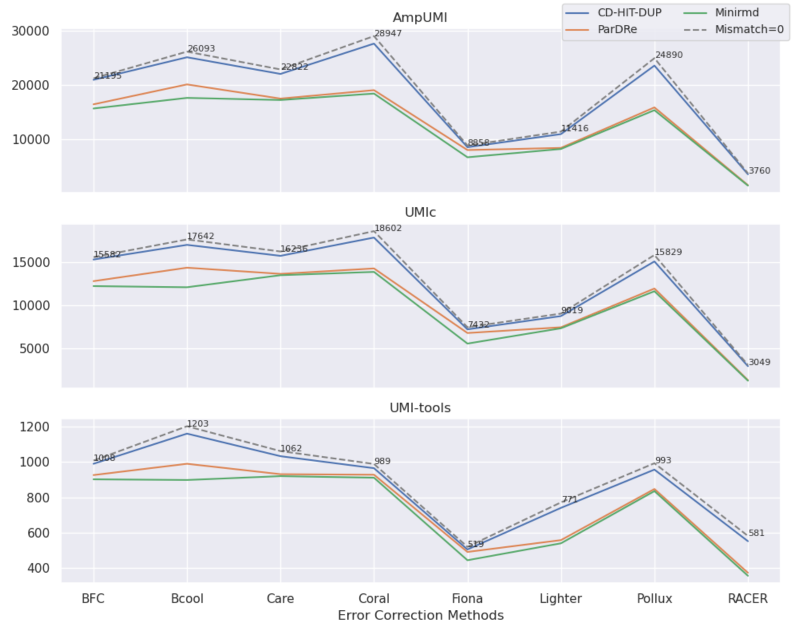

Figure S54: Line charts comparing the overlapped read numbers in the deduplicated read set by the PCR-deduplication methods of CD-HIT-DUP, ParDRe, and Minirmd on the error-corrected dataset SRR1543971, with each UMI-based PCR-deduplication methods of UMI-tools, AmpUMI, and UMIc on dataset SRR1543971. Error correction was performed using error-correction methods of BFC, Bcool, Care, Coral, Fiona, Lighter, Pollux, and RACER, respectively. CD-HIT-DUP, ParDRe, and Minirmd employed a mismatched number set to 3. The dashed line labelled 'Mismatch=0' represents results obtained by CD-HIT-DUP with a mismatch setting of 0.

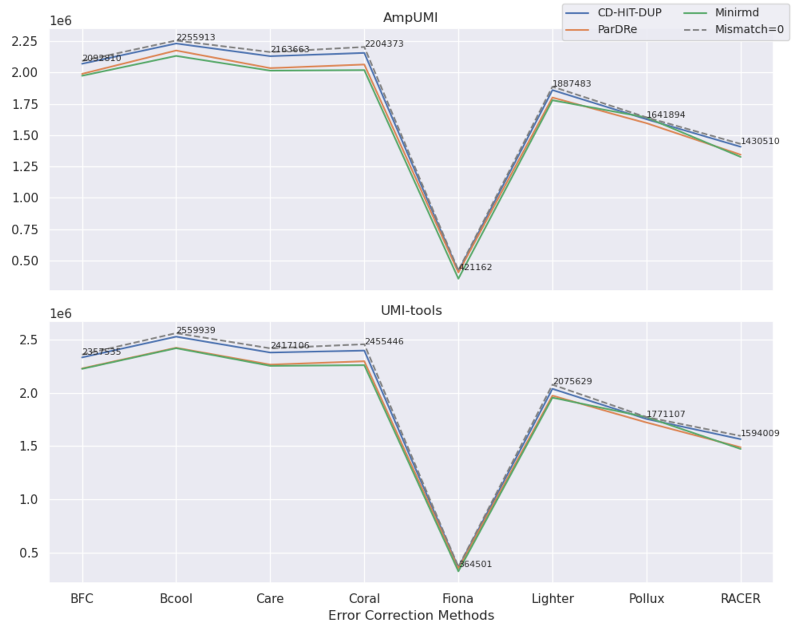

Figure S55: Line charts comparing the overlapped read numbers in the deduplicated read set by the PCR-deduplication methods of CD-HIT-DUP, ParDRe, and Minirmd on the error-corrected dataset SRR28313972, with each UMI-based PCR-deduplication methods of UMI-tools and AmpUMI on dataset SRR28313972. Error correction was performed using error-correction methods of BFC, Bcool, Care, Coral, Fiona, Lighter, Pollux, and RACER, respectively. CD-HIT-DUP, ParDRe, and Minirmd employed a mismatched number set to 1. The dashed line labelled 'Mismatch=0' represents results obtained by CD-HIT-DUP with a mismatch setting of 0.

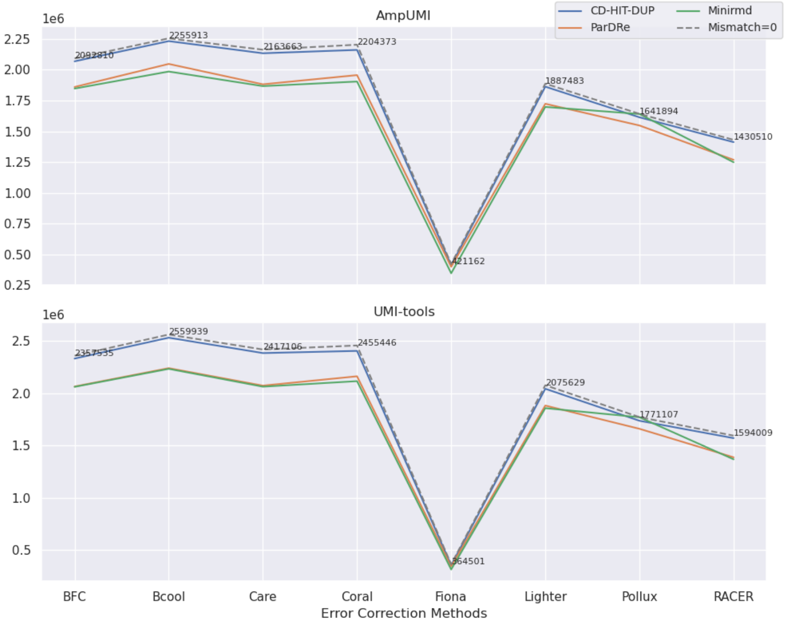

Figure S56: Line charts comparing the overlapped read numbers in the deduplicated read set by the PCR-deduplication methods of CD-HIT-DUP, ParDRe, and Minirmd on the error-corrected dataset SRR28313972, with each UMI-based PCR-deduplication methods of UMI-tools and AmpUMI on dataset SRR28313972. Error correction was performed using error-correction methods of BFC, Bcool, Care, Coral, Fiona, Lighter, Pollux, and RACER, respectively. CD-HIT-DUP, ParDRe, and Minirmd employed a mismatched number set to 2. The dashed line labelled 'Mismatch=0' represents results obtained by CD-HIT-DUP with a mismatch setting of 0.

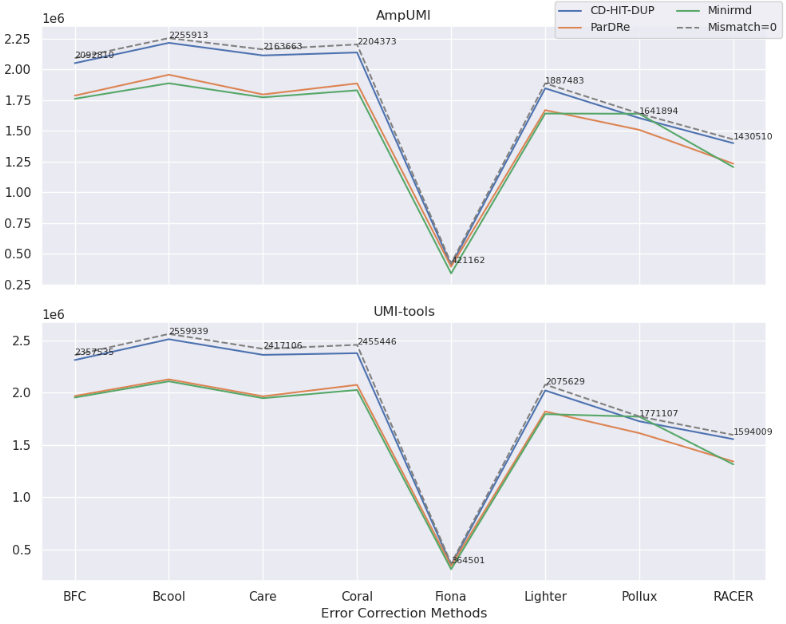

Figure S57: Line charts comparing the overlapped read numbers in the deduplicated read set by the PCR-deduplication methods of CD-HIT-DUP, ParDRe, and Minirmd on the error-corrected dataset SRR28313972, with each UMI-based PCR-deduplication methods of UMI-tools and AmpUMI on dataset SRR28313972. Error correction was performed using error-correction methods of BFC, Bcool, Care, Coral, Fiona, Lighter, Pollux, and RACER, respectively. CD-HIT-DUP, ParDRe, and Minirmd employed a mismatched number set to 3. The dashed line labelled 'Mismatch=0' represents results obtained by CD-HIT-DUP with a mismatch setting of 0.

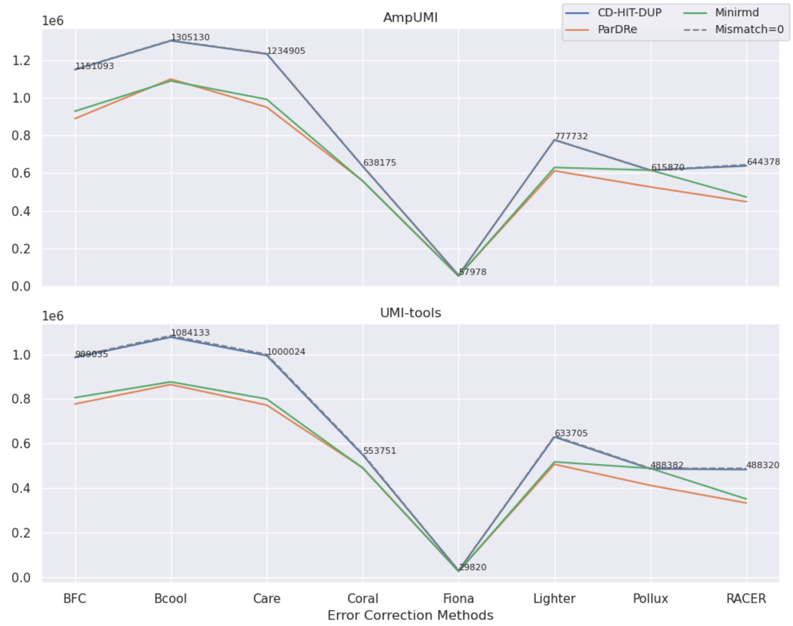

Figure S58: Line charts comparing the overlapped read numbers in the deduplicated read set by the PCR-deduplication methods of CD-HIT-DUP, ParDRe, and Minirmd on the error-corrected dataset SRR28313990, with each UMI-based PCR-deduplication methods of UMI-tools and AmpUMI on dataset SRR28313990. Error correction was performed using error-correction methods of BFC, Bcool, Care, Coral, Fiona, Lighter, Pollux, and RACER, respectively. CD-HIT-DUP, ParDRe, and Minirmd employed a mismatched number set to 1. The dashed line labelled 'Mismatch=0' represents results obtained by CD-HIT-DUP with a mismatch setting of 0.

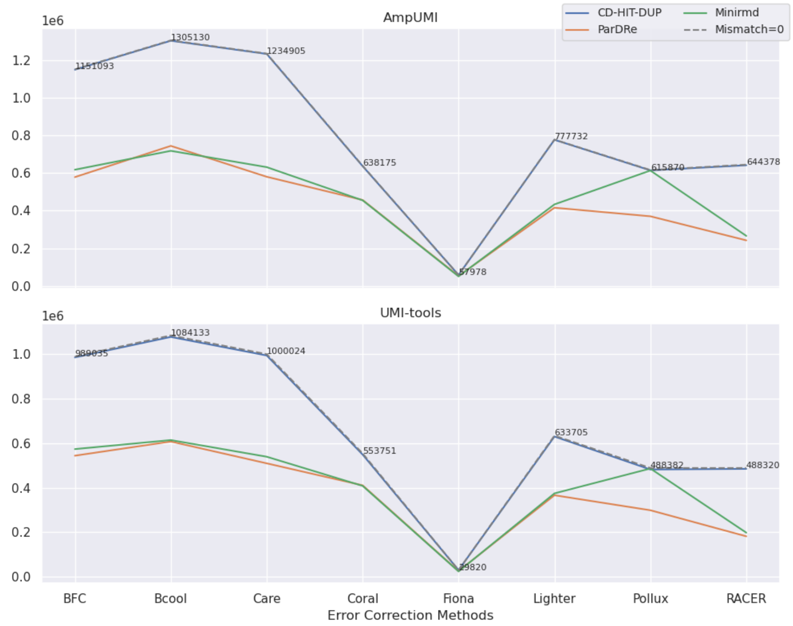

Figure S59: Line charts comparing the overlapped read numbers in the deduplicated read set by the PCR-deduplication methods of CD-HIT-DUP, ParDRe, and Minirmd on the error-corrected dataset SRR28313990, with each UMI-based PCR-deduplication methods of UMI-tools and AmpUMI on dataset SRR28313990. Error correction was performed using error-correction methods of BFC, Bcool, Care, Coral, Fiona, Lighter, Pollux, and RACER, respectively. CD-HIT-DUP, ParDRe, and Minirmd employed a mismatched number set to 2. The dashed line labelled 'Mismatch=0' represents results obtained by CD-HIT-DUP with a mismatch setting of 0.

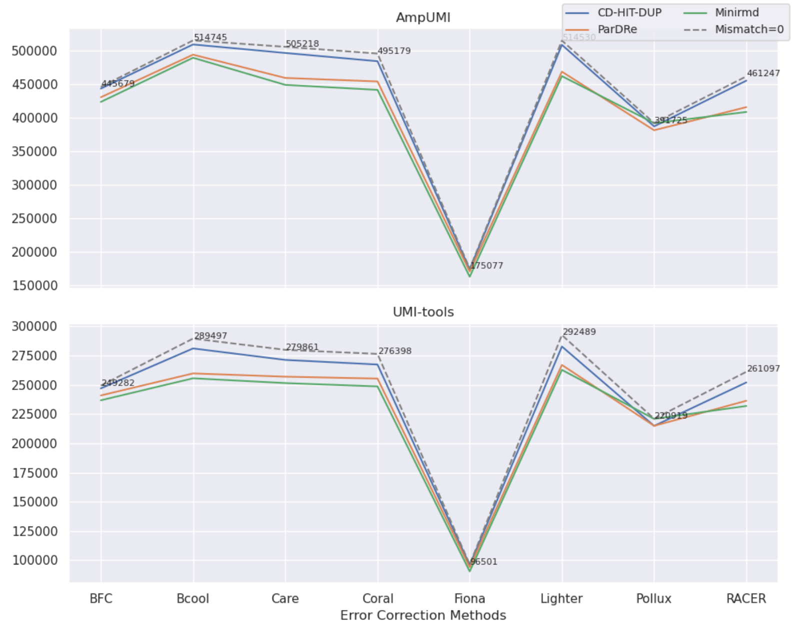

Figure S60: Line charts comparing the overlapped read numbers in the deduplicated read set by the PCR-deduplication methods of CD-HIT-DUP, ParDRe, and Minirmd on the error-corrected dataset SRR28314008, with each UMI-based PCR-deduplication methods of UMI-tools and AmpUMI on dataset SRR28314008. Error correction was performed using error-correction methods of BFC, Bcool, Care, Coral, Fiona, Lighter, Pollux, and RACER, respectively. CD-HIT-DUP, ParDRe, and Minirmd employed a mismatched number set to 1. The dashed line labelled 'Mismatch=0' represents results obtained by CD-HIT-DUP with a mismatch setting of 0.

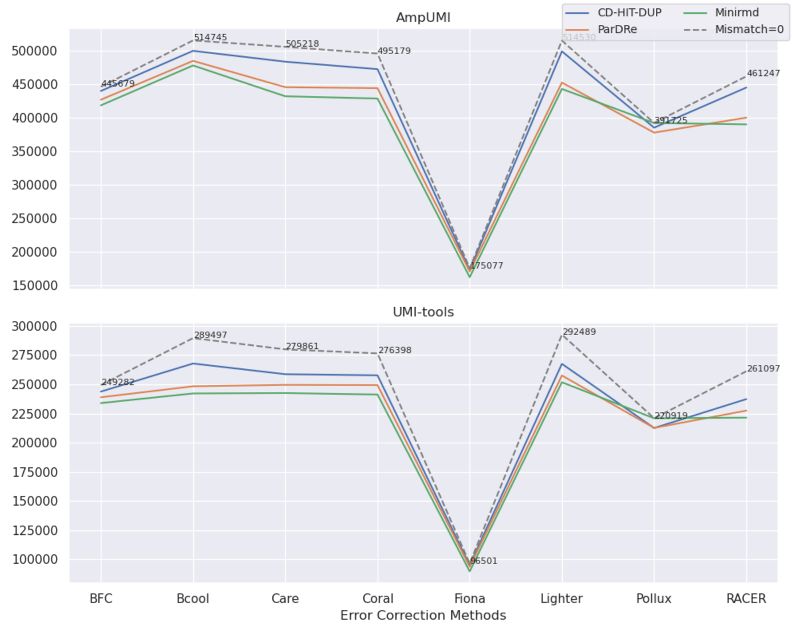

Figure S61: Line charts comparing the overlapped read numbers in the deduplicated read set by the PCR-deduplication methods of CD-HIT-DUP, ParDRe, and Minirmd on the error-corrected dataset SRR28314008, with each UMI-based PCR-deduplication methods of UMI-tools and AmpUMI on dataset SRR28314008. Error correction was performed using error-correction methods of BFC, Bcool, Care, Coral, Fiona, Lighter, Pollux, and RACER, respectively. CD-HIT-DUP, ParDRe, and Minirmd employed a mismatched number set to 2. The dashed line labelled 'Mismatch=0' represents results obtained by CD-HIT-DUP with a mismatch setting of 0.

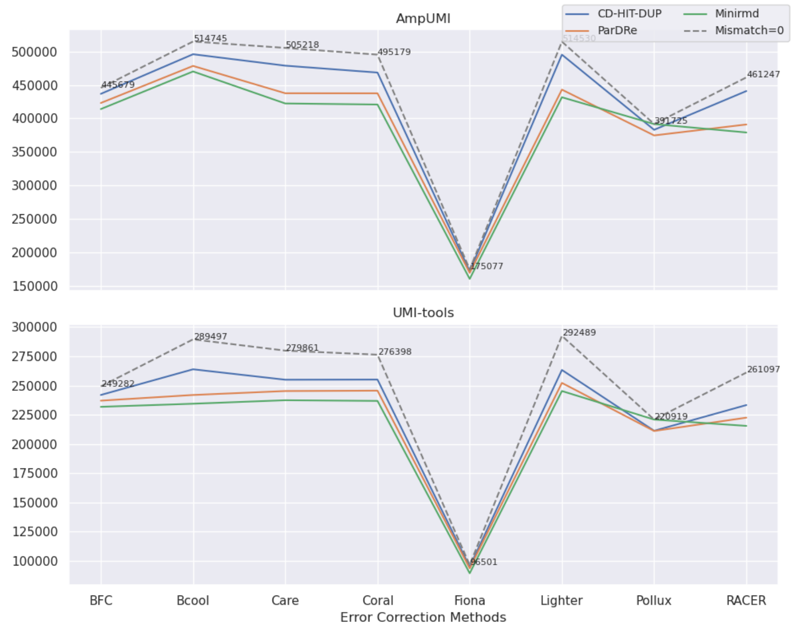

Figure S62: Line charts comparing the overlapped read numbers in the deduplicated read set by the PCR-deduplication methods of CD-HIT-DUP, ParDRe, and Minirmd on the error-corrected dataset SRR28314008, with each UMI-based PCR-deduplication methods of UMI-tools and AmpUMI on dataset SRR28314008. Error correction was performed using error-correction methods of BFC, Bcool, Care, Coral, Fiona, Lighter, Pollux, and RACER, respectively. CD-HIT-DUP, ParDRe, and Minirmd employed a mismatched number set to 3. The dashed line labelled ‘Mismatch=0’ represents results obtained by CD-HIT-DUP with a mismatch setting of 0.

Table S1: Unique reads number comparison after PCR-deduplication by the methods of UMI-tools, AmpUMI, Calib, UMIc, NGSReadsTreatment, Nubeam-dedup, BioSeqZip, fastp, FastUniq, pRESTO, CD-HIT-DUP, ParDRe and Minirmd.

Dataset	Original	UMI-tools	AmpUMI	Calib	UMIc	NGSReadsTreatment	Nubeam-dedup	BioSeqZip	fastp	FastUniq
SRR1543964	312070	38589	50458	/	36998	312070	312070	312070	312065	/
SRR1543965	273707	35439	48156	/	31629	273707	273707	273707	273698	/
SRR1543966	305104	30498	52157	/	31961	305104	305104	305104	305097	/
SRR1543967	336566	36718	57444	/	37253	336566	336566	336566	336559	/
SRR1543968	277589	30517	47395	/	30688	277589	277589	277589	277586	/
SRR1543969	375335	40227	63017	/	43897	375335	375335	375335	375324	/
SRR1543970	290058	28620	46942	/	29538	290058	290058	290058	290053	/
SRR1543971	182485	24245	33352	/	22995	182485	182485	182485	182481	/
SRR28313972	5504307	5034462	2412798	/	/	5504307	5498421	5504307	5468005	/
SRR28313990	2669507	1232133	1316953	/	/	2669507	2669402	2669507	2640309	/
SRR28314008	1696037	703947	598588	/	/	1696037	1693794	1696037	1669773	/
SRR11207257	7938261	/	421653	6401766	/	7938261	7819073	7938261	10953857	7938261
	7660776	/	331566	6410184	/	7660776	7482502	7660776	11962077	7660776

Dataset	pRESTO[a]	CD-HIT-DUP[a]	ParDRe[a]	Minirmd[a]
SRR1543964	{312069;312070;312070;312070}	{312070;295103;272744;293079}	{312070;229011;197629;180695}	{312070;221440;189201;171298}
SRR1543965	{273705;273706;273706;273706}	{273707;258877;239262;256510}	{273707;201155;174111;160366}	{273707;193622;166483;152038}
SRR1543966	{305104;305104;305104;305104}	{305104;285269;257973;282279}	{305104;225727;196468;180785}	{305104;219728;190308;174061}
SRR1543967	{336566;336566;336566;336566}	{336566;313498;282806;309886}	{336566;244839;212383;195150}	{336566;238483;205400;187461}
SRR1543968	{277587;277589;277589;277589}	{277589;257322;230970;253360}	{277589;198677;169718;153986}	{277589;193068;163597;147266}
SRR1543969	{375335;375335;375335;375335}	{375335;349495;314533;345917}	{375335;272897;232328;209753}	{375335;266857;224950;201107}
SRR1543970	{290057;290057;290058;290058}	{290058;270046;242548;267170}	{290058;209248;179333;163441}	{290058;202770;172885;156358}
SRR1543971	{182484;182485;182485;182485}	{182485;170008;153520;167025}	{182485;133523;116273;107141}	{182485;129762;112676;103228}
SRR28313972	{5484192;5496068;5496072;5496074}	{5504307;5364504;5391771;5313098}	{5504307;5074083;4689876;4451895}	{5504307;5002268;4604781;4349086}
SRR28313990	{2655692;2661654;2661661;2661662}	{2669507;2564944;2575765;2502712}	{2669507;2012208;1371720;1001412}	{2669507;2042730;1401790;1014054}
SRR28314008	{1694962;1695980;1695993;1695994}	{1696037;1559453;1343804;1283796}	{1696037;1166751;972934;880270}	{1696037;1107100;887314;777288}
SRR11207257	{7938261;7938261;7938261;7938261}	{7938261;7938261;7938261;7938261}	{7938261;6390910;5581983;5249419}	{7938261;6548964;5750100;5291977}
	{7656046;7656184;7656243;7656256}	{7660776;6589492;7138851;6549694}	{7660776;5825000;4525245;3957210}	{7660776;5991473;4847956;4242540}

[a] These methods allow mismatches when PCR-deduplication and their values in {} are obtained by setting mismatch numbers as {0, 1, 2, 3}.
The symbol '/' signifies that this method does not support a single/pair-end data set for PCR-deduplication, or that it cannot produce results when executed on the corresponding data set.

Table S2: Summary of changes in unique reads, corrected reads, and erroneously introduced new reads after error correction using the error-correction methods of BFC, Bcool, Care, Coral, Fiona, Lighter, Pollux and RACER on the datasets SRR1543965-SRR1543969.

**Datasets**	**Methods**	**The number of unique reads**			**Corrected**	**Total**	**Corrected Percentage**	**New Reads Number**
		**before correction**	**after correction**	**decreased by**	**reads number**			
SRR1543965	Bcool	273707	225375	17.66%	77398	952554	8.13%	13430
	BFC		272329	0.50%	96237		10.10%	89155
	Care		191284	30.11%	95473		10.02%	8707
	Coral		247341	9.63%	37472		3.93%	4534
	Fiona		169730	37.99%	327099		34.34%	107473
	Lighter		174343	36.30%	433875		45.55%	118246
	Pollux		237835	13.11%	132427		13.90%	31953
	RACER		250580	8.45%	825232		86.63%	230539
SRR1543966	Bcool	305104	260389	14.66%	71497	1225804	5.83%	16316
	BFC		302654	0.80%	120505		9.83%	103804
	Care		267511	12.32%	45436		3.71%	6206
	Coral		271573	10.99%	42835		3.49%	3057
	Fiona		191718	37.16%	291728		23.80%	122048
	Lighter		193137	36.70%	508163		41.46%	128164
	Pollux		271472	11.02%	149290		12.18%	28107
	RACER		257834	15.49%	1114938		90.96%	247740
SRR1543967	Bcool	336566	280936	16.53%	92368	1404275	6.58%	17989
	BFC		335031	0.46%	129612		9.23%	116100
	Care		266289	20.88%	80193		5.71%	6978
	Coral		303146	9.93%	44945		3.20%	5196
	Fiona		211267	37.23%	344803		24.55%	137048
	Lighter		217844	35.27%	509769		36.30%	128122
	Pollux		296749	11.83%	173284		12.34%	29323
	RACER		308299	8.40%	1245191		88.67%	289427
SRR1543968	Bcool	277589	227312	18.11%	95326	1132736	8.42%	13847
	BFC		275656	0.70%	114891		10.14%	105263
	Care		198186	28.60%	88941		7.85%	6611
	Coral		251775	9.30%	35155		3.10%	2468
	Fiona		169764	38.84%	299686		26.46%	105442
	Lighter		162886	41.32%	515265		45.49%	113154
	Pollux		240844	13.24%	151317		13.36%	24506
	RACER		254251	8.41%	1045490		92.30%	243957
SRR1543969	Bcool	375335	325515	13.27%	92941	1338638	6.94%	30150
	BFC		373174	0.58%	158448		11.84%	143477
	Care		263721	29.74%	128400		9.59%	12058
	Coral		331403	11.70%	60184		4.50%	5044
	Fiona		234396	37.55%	377712		28.22%	152277
	Lighter		224814	40.10%	583947		43.62%	141116
	Pollux		319236	14.95%	195977		14.64%	31433
	RACER		315614	15.91%	1199132		89.58%	291860

Table S3: Summary of changes in unique reads, corrected reads, and erroneously introduced new reads after error correction using the error-correction methods of BFC, Bcool, Care, Coral, Fiona, Lighter, Pollux and RACER on the datasets SRR1543970-SRR1543971, SRR28313990 and SRR28314008.

Datasets	Methods	The number of unique reads			Corrected	Total	Corrected	New Reads
		before correction	after correction	decreased by	reads number		Percentage	Number
SRR1543970	Bcool	290058	238083	17.92%	80100	1219666	6.57%	14812
	BFC		287510	0.88%	118777		9.74%	105239
	Care		252010	13.12%	46465		3.81%	6768
	Coral		250633	13.59%	48603		3.98%	3098
	Fiona		171523	40.87%	292715		24.00%	118053
	Lighter		186026	35.87%	430315		35.28%	95734
	Pollux		252119	13.08%	134531		11.03%	28292
	RACER		257492	11.23%	1106449		90.72%	245119
SRR1543971	Bcool	182485	146958	19.47%	50130	746724	6.71%	7379
	BFC		181088	0.77%	76835		10.29%	70518
	Care		129353	29.12%	61209		8.20%	5329
	Coral		157737	13.56%	34687		4.65%	4064
	Fiona		111720	38.78%	231785		31.04%	65538
	Lighter		125096	31.45%	265978		35.62%	76783
	Pollux		158233	13.29%	94785		12.69%	19978
	RACER		189741	-3.98%	637304		85.35%	173945
SRR28313990	Bcool	2669507	2587568	3.07%	205105	5943780	3.45%	4599
	Care		2499211	6.38%	320028		5.38%	141238
	Coral		1614311	39.53%	1572630		26.46%	303701
	Fiona		1691600	36.63%	4054483		68.21%	1613490
	Lighter		2457358	7.95%	1439218		24.21%	852568
	Pollux		1668321	37.50%	1811727		30.48%	407231
	RACER		2953113	-10.62%	2191865		36.88%	1723226
	BFC		2678966	-0.35%	699713		11.77%	603516
SRR28314008	Bcool	1696037	1272751	24.96%	673892	7563556	8.91%	24329
	Care		1249364	26.34%	603932		7.98%	106565
	Coral		1179827	30.44%	743128		9.83%	132303
	Fiona		774328	54.34%	3325931		43.97%	547459
	Lighter		1324479	21.91%	653864		8.64%	46386
	Pollux		826139	51.29%	1435655		18.98%	238401
	RACER		1417912	16.40%	948832		12.54%	261460
	BFC		1678715	1.02%	1149440		15.20%	964572
